# Long-term neurologic outcomes of COVID-19

**DOI:** 10.1038/s41591-022-02001-z

**Published:** 2022-09-22

**Authors:** Evan Xu, Yan Xie, Ziyad Al-Aly

**Affiliations:** 1 Clinical Epidemiology Center, Research and Development Service, VA St. Louis Health Care System, St. Louis, MO USA; 2 Veterans Research and Education Foundation of St. Louis, St. Louis, MO USA; 3 Department of Epidemiology and Biostatistics, College for Public Health and Social Justice, Saint Louis University, St. Louis, MO USA; 4 Department of Medicine, Washington University School of Medicine, St. Louis, MO USA; 5 Nephrology Section, Medicine Service, VA St. Louis Health Care System, St. Louis, MO USA; 6 Institute for Public Health, Washington University in St. Louis, St. Louis, MO USA

**Keywords:** SARS-CoV-2, Neurological disorders, Viral infection, Viral infection

## Abstract

The neurologic manifestations of acute COVID-19 are well characterized, but a comprehensive evaluation of postacute neurologic sequelae at 1 year has not been undertaken. Here we use the national healthcare databases of the US Department of Veterans Affairs to build a cohort of 154,068 individuals with COVID-19, 5,638,795 contemporary controls and 5,859,621 historical controls; we use inverse probability weighting to balance the cohorts, and estimate risks and burdens of incident neurologic disorders at 12 months following acute SARS-CoV-2 infection. Our results show that in the postacute phase of COVID-19, there was increased risk of an array of incident neurologic sequelae including ischemic and hemorrhagic stroke, cognition and memory disorders, peripheral nervous system disorders, episodic disorders (for example, migraine and seizures), extrapyramidal and movement disorders, mental health disorders, musculoskeletal disorders, sensory disorders, Guillain–Barré syndrome, and encephalitis or encephalopathy. We estimated that the hazard ratio of any neurologic sequela was 1.42 (95% confidence intervals 1.38, 1.47) and burden 70.69 (95% confidence intervals 63.54, 78.01) per 1,000 persons at 12 months. The risks and burdens were elevated even in people who did not require hospitalization during acute COVID-19. Limitations include a cohort comprising mostly White males. Taken together, our results provide evidence of increased risk of long-term neurologic disorders in people who had COVID-19.

## Abstract (web summary)

Individuals with COVID-19 are at an increased risk for an array of neurologic disorders at 12 months, even in those who were not hospitalized during the acute phase of the infection.

## Main

Long COVID—the umbrella term describing the constellation of postacute sequelae following infection with SARS-CoV-2—can involve a broad array of extrapulmonary organ dysfunction^1^ including several structural neurologic abnormalities^2^. To date, most studies examining postacute COVID-19 clinical neurologic disorders were limited to people who were hospitalized during the acute phase of COVID-19, and all studies had follow-up duration of less than 6 months with a narrow selection of neurologic outcomes^3–8^. A comprehensive evaluation of postacute COVID-19 neurologic outcomes at 12 months is needed but has not yet been undertaken. Studies of postacute COVID-19 neurologic outcomes across the care-setting spectrum of the acute phase of the disease (nonhospitalized, hospitalized and admitted to intensive care) are also not yet available. Addressing this knowledge gap is important in helping guide postacute COVID-19 care strategies and healthcare system capacity planning.

Here we leverage the breadth and depth of the US Department of Veterans Affairs national healthcare databases to build a cohort of 154,068 people who survived the first 30 days of COVID-19 and two control groups: a contemporary cohort consisting of 5,638,795 users of the US Department of Veterans Health Care System (VHA) with no evidence of SARS-CoV-2 infection, and a historical cohort (predating the global COVID-19 pandemic) consisting of 5,859,621 VHA users during 2017. We employed a longitudinal observational study design and used inverse probability weighting to balance the cohorts, and estimated the risks and burdens at 12 months of a set of prespecified neurologic outcomes in the overall cohort and by care setting of the acute phase of COVID-19 (nonhospitalized, hospitalized and admitted to intensive care).

## Results

**Fig. 1:**
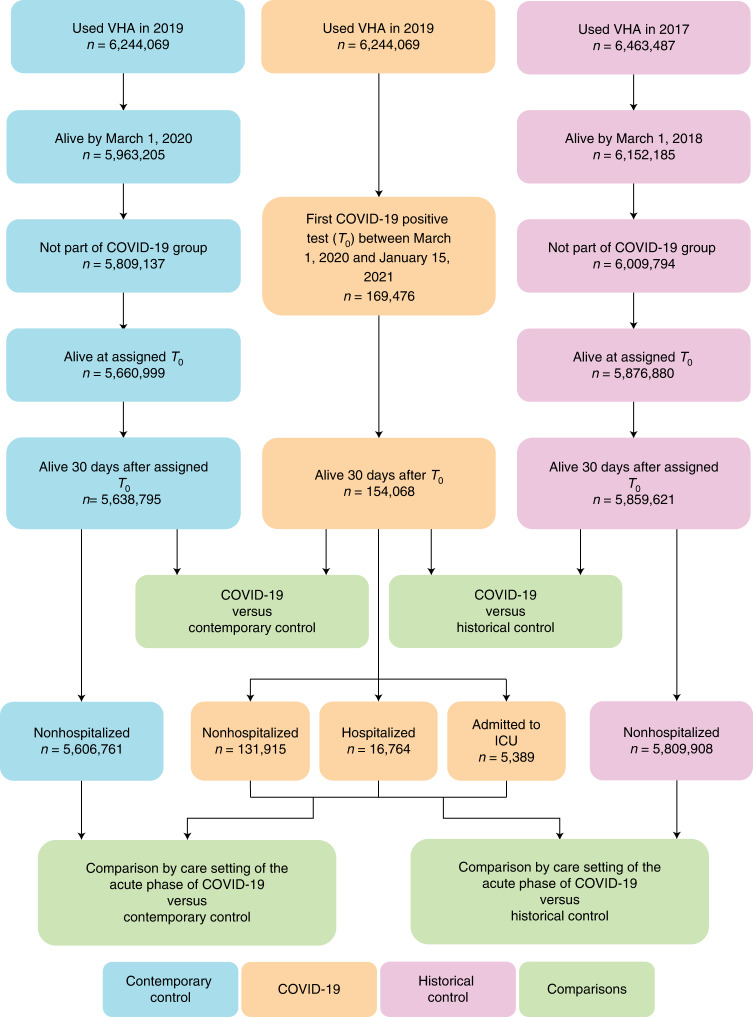
Cohort construction flowchart. Cohort construction for COVID-19 group (blue), contemporary control group (orange) and historical control group (pink). Comparisons between groups are presented in green.

There were 154,068, 5,638,795 and 5,859,621 participants in the COVID-19, the contemporary control and the historical control groups, respectively (Fig. 1). Median follow-up time in the COVID-19, contemporary control and historical control groups was 408 (interquartile range: 378–500), 409 (379–505) and 409 (379–504) days, respectively. The COVID-19, contemporary control and historical control groups had 185,399, 6,808,464 and 7,071,123 person-years of follow up, respectively; altogether corresponding to 14,064,985 person-years of follow up.

The demographic and health characteristics of the COVID-19, the contemporary control and historical control groups before and after weighting are presented in Supplementary Tables 1 and 2, respectively.

### Incident neurologic outcomes in COVID-19 versus contemporary control

**Extended Data Fig. 1:**
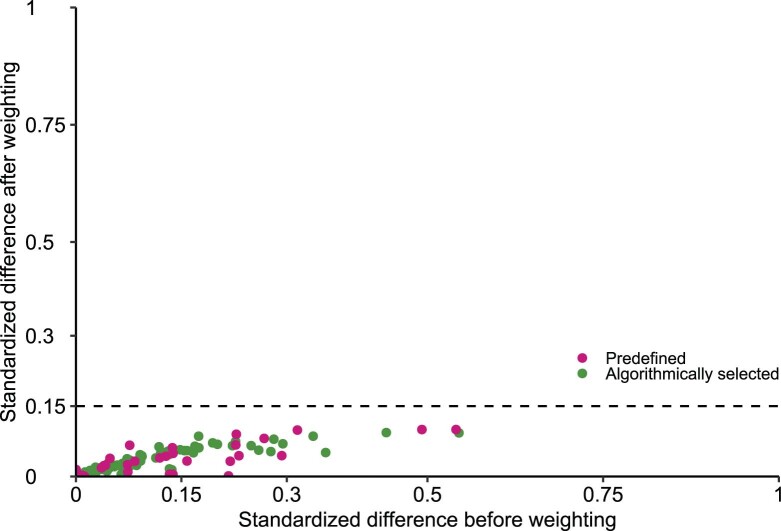
Standardized mean difference of predefined and algorithmically selected high-dimensional variables. Standardized mean difference between COVID-19 and contemporary control.

We used the inverse probability weighting method to balance the COVID-19 and the contemporary control groups; examination of standardized mean differences of demographic and health characteristics after weighting suggested good balance (Extended Data Fig. 1).

**Fig. 2:**
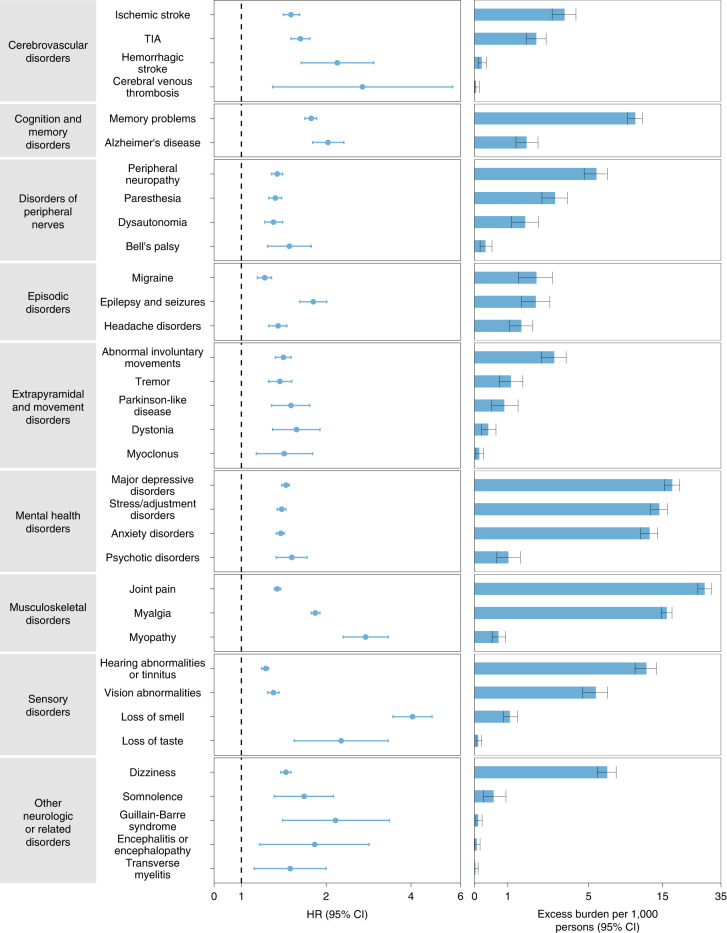
Risks and 12-month burdens of incident postacute COVID-19 neurologic outcomes compared with the contemporary control cohort. Outcomes were ascertained 30 days after the COVID-19-positive test until the end of follow up. COVID-19 cohort (*n* = 154,068) and contemporary control cohort (*n* = 5,638,795). Adjusted HRs (dots) and 95% (error bars) CIs are presented, as are estimated excess burdens (bars) and 95% CIs (error bars). Burdens are presented per 1,000 persons at 12 months of follow up. The dashed line marks a HR of 1.00; lower limits of 95% CIs with values greater than 1.00 indicate significantly increased risk.

**Fig. 3:**
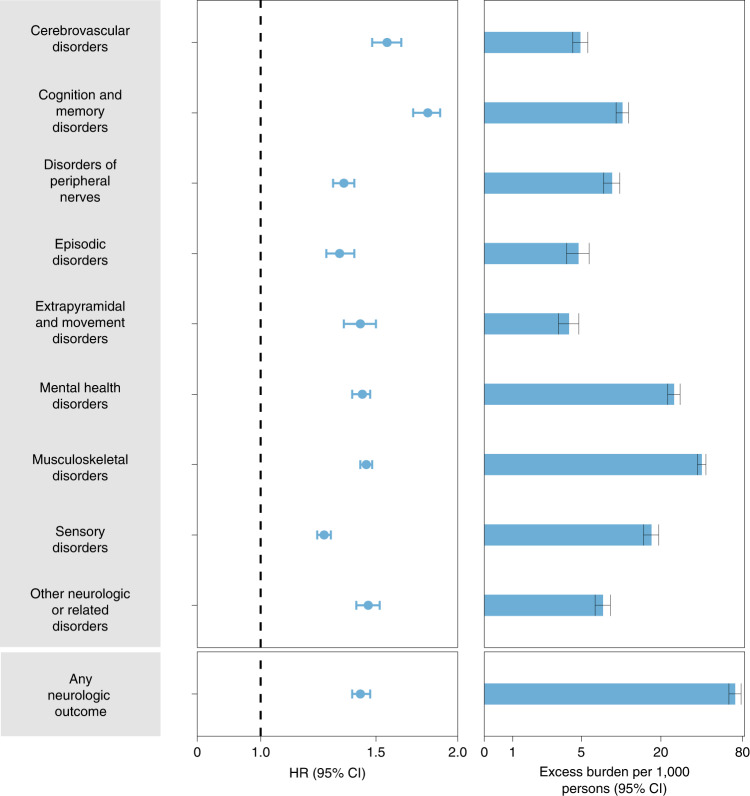
Risks and 12-month burdens of incident postacute COVID-19 composite neurologic outcomes compared with the contemporary control cohort. Composite outcomes consisted of cerebrovascular disorders (ischemic stroke, TIA, hemorrhagic stroke and cerebral venous thrombosis), cognition and memory (memory problems and Alzheimer’s disease), disorders of the peripheral nerves (peripheral neuropathy, paresthesia, dysautonomia and Bell’s palsy), episodic disorders (migraine, epilepsy and seizures and headache disorders), extrapyramidal and movement disorders (abnormal involuntary movements, tremor, Parkinson-like disease, dystonia, myoclonus), mental health disorders (major depressive disorders, stress and adjustment disorders, anxiety disorders, and psychotic disorders), musculoskeletal disorders (joint pain, myalgia and myopathy), sensory disorders (hearing abnormalities or tinnitus, vision abnormalities, loss of smell and loss of taste), other neurologic or related disorders (dizziness, somnolence, Guillain–Barré syndrome, encephalitis or encephalopathy and transverse myelitis) and any neurologic outcome (incident occurrence of any neurologic outcome studied). Outcomes were ascertained 30 days after the COVID-19-positive test until the end of follow up. The COVID-19 cohort had *n* = 154,068 and the contemporary control cohort had *n* = 5,638,795. Adjusted HRs (dots) and 95% (error bars) CIs are presented, as are estimated excess burdens (bars) and 95% CIs (error bars). Burdens are presented per 1,000 persons at 12 months of follow up. The dashed line marks a HR of 1.00; lower limits of 95% CIs with values greater than 1.00 indicate significantly increased risk.

We estimated the risks of a set of prespecified neurologic outcomes in COVID-19 versus the contemporary control group; we also estimated the adjusted excess burden of neurologic outcomes due to COVID-19 per 1,000 persons at 12 months on the basis of the difference between the estimated incidence rate in the COVID-19 and contemporary control groups. Risks and burdens of individual neurologic outcomes are provided in Fig. 2 and Supplementary Table 3 and are discussed below. Risks and burdens of the composite endpoints are provided in Fig. 3 and Supplementary Table 3.

#### Cerebrovascular disorders

People who survived the first 30 days of COVID-19 exhibited increased risk of ischemic stroke (HR 1.50 (1.41, 1.61); burden 3.40 (2.75, 4.09) per 1,000 persons at 12 months; for all HRs and burdens, parenthetical ranges refer to 95% confidence intervals (CIs)), transient ischemic attacks (TIAs) (HR 1.62 (1.50, 1.75); burden 2.03 (1.64, 2.46)), hemorrhagic stroke (HR 2.19 (1.63, 2.95); burden 0.21 (0.11, 0.35)) and cerebral venous thrombosis (HR 2.69 (1.29, 5.62); burden 0.05 (0.01, 0.14)). The risk and burden of a composite of these cerebrovascular outcomes were 1.56 (1.48, 1.64) and 4.92 (4.26, 5.62), respectively.

#### Cognition and memory

There were increased risks of memory problems (HR 1.77 (1.68, 1.85); burden 10.07 (9.00, 11.20)) and Alzheimer’s disease (HR 2.03 (1.79, 2.31); burden 1.65 (1.27, 2.10)). The risk and burden of a composite of these cognition and memory outcomes were 1.80 (1.71, 1.88) and 10.35 (9.27, 11.47), respectively.

#### Disorders of peripheral nerves

These included peripheral neuropathy (HR 1.34 (1.28, 1.40); burden 5.64 (4.67, 6.65)), paresthesia (HR 1.32 (1.25, 1.39); burden 2.89 (2.27, 3.55)), dysautonomia (HR 1.30 (1.21, 1.40); burden 1.60 (1.12, 2.12)) and Bell’s palsy (HR 1.48 (1.24, 1.77)); burden 0.32 (0.16, 0.51)). The respective risk and burden of a composite of these disorders of peripheral nerves were 1.34 (1.29, 1.39) and 8.64 (7.44, 9.87).

#### Episodic disorders

Episodic disorders included migraine (HR 1.21 (1.14, 1.28); burden 2.04 (1.36, 2.76)), epilepsy and seizures (HR 1.80 (1.61, 2.01); burden 2.01 (1.47, 2.63)) and headache disorders (HR 1.35 (1.25, 1.45); burden 1.46 (1.06, 1.89)). The risk and burden of a composite of these episodic disorders were 1.32 (1.26, 1.39) and 4.75 (3.79, 5.76), respectively.

#### Extrapyramidal and movement disorders

These included abnormal involuntary movements (HR 1.41 (1.32, 1.50); burden 2.85 (2.24, 3.49)), tremor (HR 1.37 (1.25, 1.51); burden 1.10 (0.73, 1.51)), Parkinson-like disease (HR 1.50 (1.28, 1.75); burden 0.89 (0.50, 1.34)), dystonia (HR 1.57 (1.29, 1.90); burden 0.40 (0.21, 0.63)) and myoclonus (HR 1.42 (1.13, 1.79); burden 0.14 (0.04, 0.26)). The respective risk and burden of a composite of these extrapyramidal and movement disorders were 1.42 (1.34, 1.50) and 3.98 (3.24, 4.77).

#### Mental health disorders

Mental health disorders included major depressive disorders (HR 1.44 (1.39, 1.48); burden 17.28 (15.43, 19.18)), stress and adjustment disorders (HR 1.39 (1.34, 1.44); burden 14.34 (12.66, 16.07)), anxiety disorders (HR 1.38 (1.33, 1.42); burden 12.44 (10.93, 13.99)) and psychotic disorders (HR 1.51 (1.33, 1.71); burden 1.02 (0.66, 1.43)). The respective risk and burden of a composite of these mental health disorders were 1.43 (1.38, 1.47) and 25.00 (22.40, 27.69).

#### Musculoskeletal disorders

Musculoskeletal disorders included joint pain (HR 1.34 (1.31, 1.38); burden 27.65 (25.01, 30.35)), myalgia (HR 1.83 (1.77, 1.90); burden 15.97 (14.75, 17.23)) and myopathy (HR 2.76 (2.30, 3.32); burden 0.71 (0.52, 0.93)). The risk and burden of a composite of these musculoskeletal disorders were 1.45 (1.42, 1.48) and 40.09 (37.22, 43.01), respectively.

#### Sensory disorders

Sensory disorders included hearing abnormalities or tinnitus (HR 1.22 (1.18, 1.25); burden 11.87 (10.05, 13.75)), vision abnormalities (HR 1.30 (1.24, 1.36); burden 5.59 (4.55, 6.68)), loss of smell (HR 4.05 (3.45, 4.75)); burden 1.07 (0.86, 1.32)) and loss of taste (HR 2.26 (1.54, 3.32); burden 0.11 (0.05, 0.21)). The respective risk and burden of a composite of these sensory disorders were 1.25 (1.22, 1.28) and 17.03 (14.85, 19.26).

#### Other neurologic or related disorders

These included dizziness (HR 1.44 (1.38, 1.50); burden 6.65 (5.72, 7.61)), somnolence (HR 1.67 (1.31, 2.12); burden 0.56 (0.26, 0.94)), Guillain–Barré syndrome (HR 2.16 (1.40, 3.35); burden 0.11 (0.04, 0.22)), encephalitis or encephalopathy (HR 1.82 (1.16, 2.84); burden 0.07 (0.01, 0.16) and transverse myelitis (HR 1.49 (1.11, 2.00); burden 0.03 (0.00, 0.11)). The respective risk and burden of a composite of these other neurologic or related disorders were 1.46 (1.40, 1.52) and 7.37 (6.41, 8.38), respectively.

#### Composite outcome of any neurologic disorder

We then examined the risk and burden of having any neurologic outcome (defined as the occurrence of any incident prespecified neurologic outcome included in this study). Compared with the contemporary control group, there was increased risk and burden of any neurologic outcome (HR 1.42 (1.38, 1.47); burden 70.69 (63.54, 78.01)), respectively.

#### Subgroup analyses

**Fig. 4:**
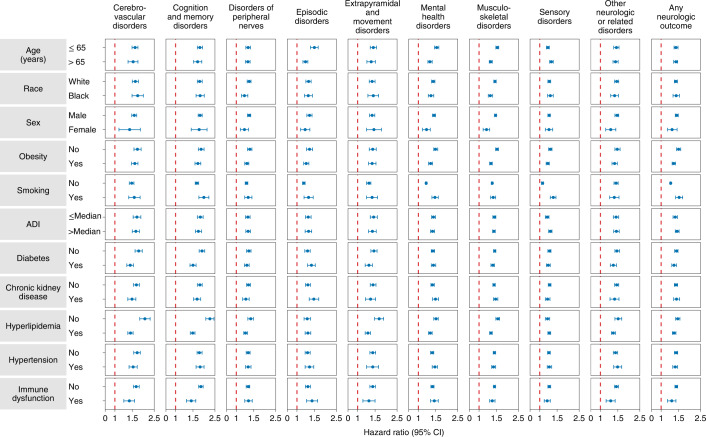
Subgroup analyses of the risks of incident postacute COVID-19 composite neurologic outcomes compared with the contemporary control cohort. Composite outcomes consisted of cerebrovascular disorders (ischemic stroke, TIA, hemorrhagic stroke and cerebral venous thrombosis), cognition and memory (memory problems and Alzheimer’s disease) disorders, disorders of the peripheral nerves (peripheral neuropathy, paresthesia, dysautonomia and Bell’s palsy), episodic disorders (migraine, epilepsy and seizures, and headache disorders), extrapyramidal and movement disorders (abnormal involuntary movements, tremor, Parkinson-like disease, dystonia, myoclonus), mental health disorders (major depressive disorders, stress and adjustment disorders, anxiety disorders, and psychotic disorders), musculoskeletal disorders (joint pain, myalgia and myopathy), sensory disorders (hearing abnormalities or tinnitus, vision abnormalities, loss of smell and loss of taste), other neurologic or related disorders (dizziness, somnolence, Guillain–Barré syndrome, encephalitis or encephalopathy and transverse myelitis) and any neurologic outcome (incident occurrence of any neurologic outcome studied). Outcomes were ascertained 30 days after the COVID-19-positive test until the end of follow up. COVID-19 cohort (*n* = 154,068) and contemporary control cohort (*n* = 5,638,795). Adjusted HRs (dots) and 95% (error bars) CIs are presented. The dashed line marks a HR of 1.00; lower limits of 95% CIs with values greater than 1.00 indicate significantly increased risk.

The risks of incident composite neurologic outcomes were evident in all subgroups based on age, race, sex, obesity, smoking, area deprivation index (ADI), diabetes, chronic kidney disease, hyperlipidemia, hypertension and immune dysfunction (Fig. 4 and Supplementary Table 4). Because of the relatively smaller size, there was greater variance (and larger CIs) in the female cohort compared with the male cohort.

**Extended Data Fig. 2:**
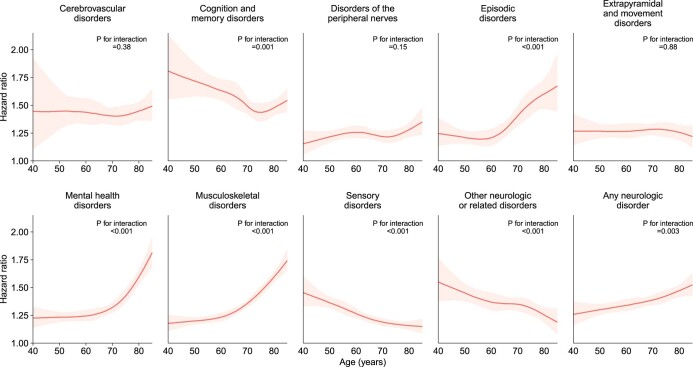
The risks of incident postacute COVID-19 composite neurologic outcomes across age compared with the contemporary control cohort. Composite outcomes consisted of cerebrovascular disorders (ischemic stroke, TIA, hemorrhagic stroke, and cerebral venous thrombosis), cognition and memory (memory problems and Alzheimer’s disease), disorders of the peripheral nerves (peripheral neuropathy, paresthesia, dysautonomia, and Bell’s palsy), episodic disorders (migraine, epilepsy and seizures, and headache disorders), extrapyramidal and movement disorders (abnormal involuntary movements, tremor, Parkinson-like disease, dystonia, myoclonus), mental health disorders (major depressive disorders, stress and adjustment disorders, anxiety disorders, and psychotic disorders), musculoskeletal disorders (joint pain, myalgia, and myopathy), sensory disorders (Hearing abnormalities or tinnitus, vision abnormalities, loss of smell, and loss of taste), other neurologic or related disorders (dizziness, somnolence, Guillain-Barré syndrome, encephalitis or encephalopathy and transverse myelitis), and any neurologic outcome (incident occurrence of any neurologic outcome studied). Outcomes were ascertained 30d after the COVID-19-positive test until the end of follow up. COVID-19 cohort (n = 154,068) and contemporary control cohort (n = 5,638,795). Age was transformed into restricted cubic spline function for the analyses. P value was based on 2-sided Wald Chi-Squared test on interaction between age and exposure, without multiple comparisons adjustment. A P value of <0.05 suggests that age modifies the association between COVID-19 and the neurologic outcome. The P value for cerebrovascular disorders=0.38, cognition and memory disorders=0.001, disorders of the peripheral nerves=0.15, episodic disorders<0.001, extrapyramidal and movement disorders=0.88, mental health disorders<0.001, musculoskeletal disorders<0.001, sensory disorders<0.001, other neurologic or related disorders<0.001, and any neurologic disorder=0.003.

Analyses of risk across age as a continuous variable suggest that the risks of incident composite neurologic outcomes were evident across the age range in this cohort. Interaction analyses between age and exposure suggested that the risks of episodic disorders, mental health disorders, musculoskeletal disorders and any neurologic disorder increased as age increased (*P* for interaction <0.001, <0.001 and 0.003, respectively), and risks of cognition and memory disorders, sensory disorders and other neurologic or related disorders decreased as age increased (*P* for interaction 0.001, <0.001, <0.001, respectively) (Extended Data Fig. 2).

### Incident neurologic disorders in COVID-19 versus contemporary controls by care setting of the acute infection

**Extended Data Fig. 3:**
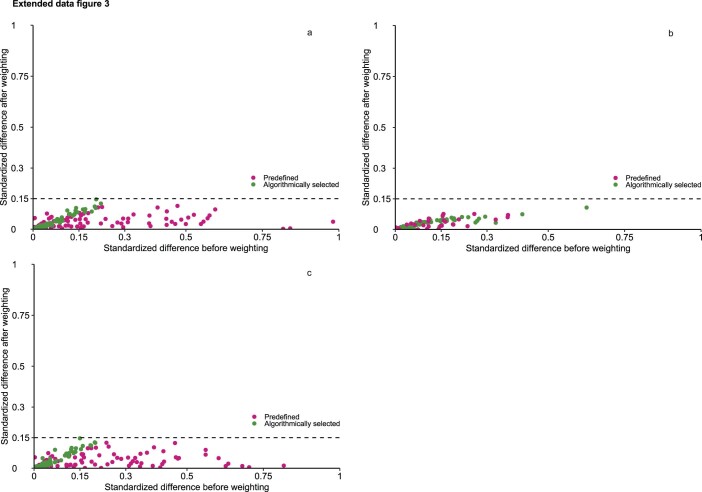
Standardized mean difference of predefined and algorithmically selected high-dimensional variables. (a) by care setting of the acute infection between COVID-19 and contemporary control; (b) between COVID-19 and historical control; (c) by care setting of the acute infection between COVID-19 and historical control.

We then examined the risks and burdens of neurologic outcomes in mutually exclusive groups by the care setting of the acute infection (whether people were nonhospitalized (*n* = 131,915), hospitalized (*n* = 16,764) or admitted to intensive care (*n* = 5,389) during the acute phase of COVID-19). The demographic and health characteristics of these three groups before and after weighting are presented in Supplementary Tables 5 and 6, respectively. Assessment of standardized mean differences after application of inverse weighting suggested that covariates were well balanced (Extended Data Fig. 3a).

**Fig. 5:**
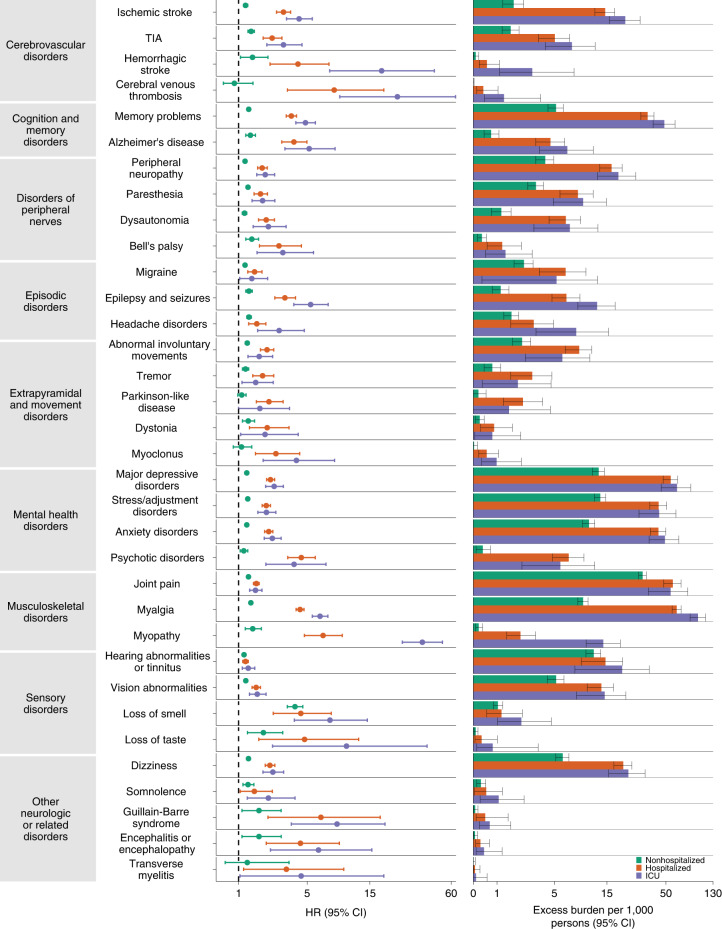
Risks and 12-month burdens of incident postacute COVID-19 neurologic outcomes compared with the contemporary control cohort by care setting of the acute infection. Risks and burdens were assessed at 12 months in mutually exclusive groups comprising nonhospitalized individuals with COVID-19 (green), individuals hospitalized for COVID-19 (orange) and individuals admitted to intensive care for COVID-19 during the acute phase (first 30 days) of COVID-19 (purple). Outcomes were ascertained 30 days after the COVID-19-positive test until the end of follow up. The contemporary control cohort served as the referent category. Within the COVID-19 cohort, nonhospitalized (*n* = 131,915), hospitalized (*n* = 16,764), admitted to intensive care (*n* = 5,389) and contemporary control cohort (*n* = 5,606,761). Adjusted HRs (dots) and 95% (error bars) CIs are presented, as are estimated excess burdens (bars) and 95% CIs (error bars). Burdens are presented per 1,000 persons at 12 months of follow up. ICU, intensive care unit. The dashed line marks a HR of 1.00; lower limits of 95% CIs with values greater than 1.00 indicate significantly increased risk.

**Fig. 6:**
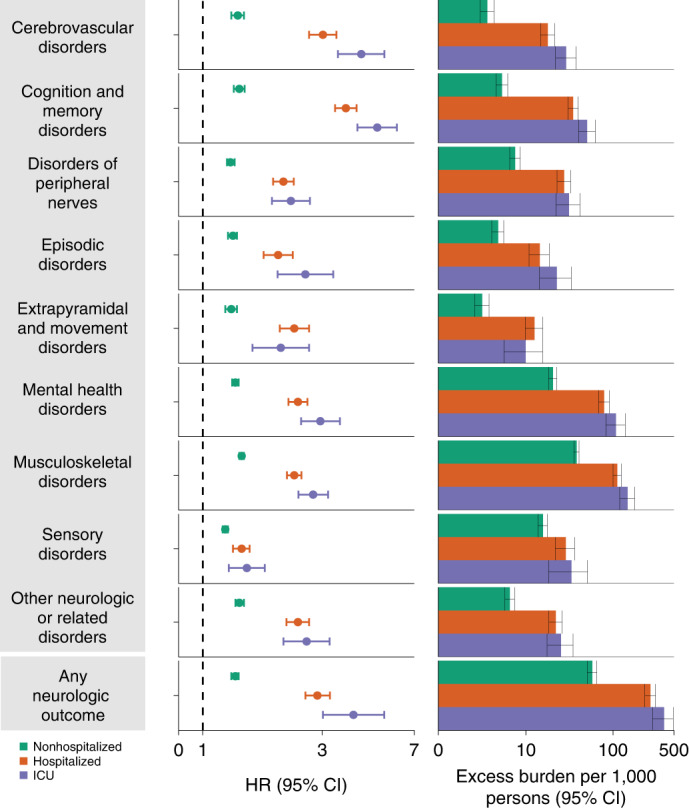
Risks and 12-month burdens of incident postacute COVID-19 composite neurologic outcomes compared with the contemporary control cohort by care setting of the acute infection. Risks and burdens were assessed at 12 months in mutually exclusive groups comprising nonhospitalized individuals with COVID-19 (green), individuals hospitalized for COVID-19 (orange) and individuals admitted to intensive care for COVID-19 during the acute phase (first 30 days) of COVID-19 (purple). Composite outcomes consisted of cerebrovascular disorders (ischemic stroke, TIA, hemorrhagic stroke and cerebral venous thrombosis), cognition and memory disorders (memory problems and Alzheimer’s disease), disorders of the peripheral nerves (peripheral neuropathy, paresthesia, dysautonomia and Bell’s palsy), episodic disorders (migraine, epilepsy and seizures, and headache disorders), extrapyramidal and movement disorders (abnormal involuntary movements, tremor, Parkinson-like disease, dystonia, myoclonus), mental health disorders (major depressive disorders, stress and adjustment disorders, anxiety disorders, and psychotic disorders), musculoskeletal disorders (joint pain, myalgia and myopathy), sensory disorders (hearing abnormalities or tinnitus, vision abnormalities, loss of smell and loss of taste), other neurologic or related disorders (dizziness, somnolence, Guillain–Barré syndrome, encephalitis or encephalopathy and transverse myelitis) and any neurologic outcome (incident occurrence of any neurologic outcome studied). Outcomes were ascertained 30 days after the COVID-19-positive test until the end of follow up. The contemporary control cohort served as the referent category. Within the COVID-19 cohort were the nonhospitalized (*n* = 131,915), hospitalized (*n* = 16,764), those admitted to intensive care (*n* = 5,389) and contemporary control cohort (*n* = 5,606,761). Adjusted HRs (dots) and 95% (error bars) CIs are presented, as are estimated excess burdens (bars) and 95% CIs (error bars). Burdens are presented per 1,000 persons at 12 months of follow up. The dashed line marks a HR of 1.00; lower limits of 95% CIs with values greater than 1.00 indicate significantly increased risk.

Compared with the contemporary control group, the risks and burdens of the prespecified neurologic outcomes were evident even among those who were not hospitalized during the acute phase of COVID-19 and increased according to the severity of the acute infection from nonhospitalized to hospitalized to those admitted to intensive care (Fig. 5 and Supplementary Table 7); results for the composite outcomes are shown in Fig. 6 and Supplementary Table 7.

### Incident neurologic disorders in COVID-19 versus historical controls

**Extended Data Fig. 4:**
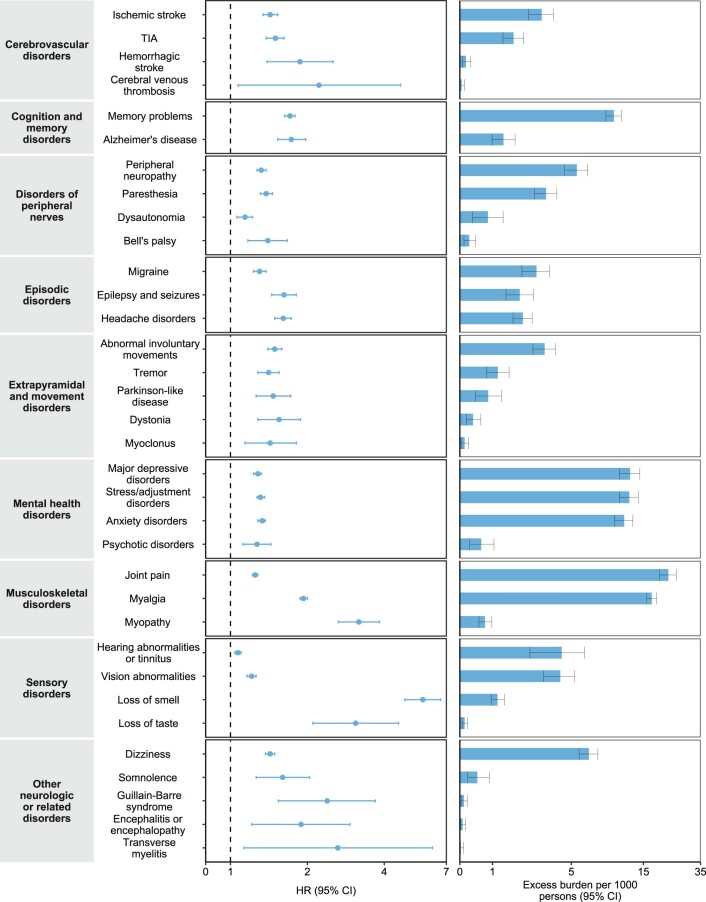
Risks and 12-month burdens of incident postacute COVID-19 neurologic outcomes compared with the historical control cohort. Outcomes were ascertained 30 d after the COVID-19-positive test until the end of follow-up. COVID-19 cohort (n = 154,068) and historical control cohort (n = 5,859,621). Adjusted HRs (dots) and 95% (error bars) CIs are presented, as are estimated excess burdens (bars) and 95% confidence intervals (error bars). Burdens are presented per 1000 persons at 12 months of follow up. The dashed line marks a HR of 1.00; lower limits of 95% CIs with values greater than 1.00 indicate significantly increased risk.

**Extended Data Fig. 5:**
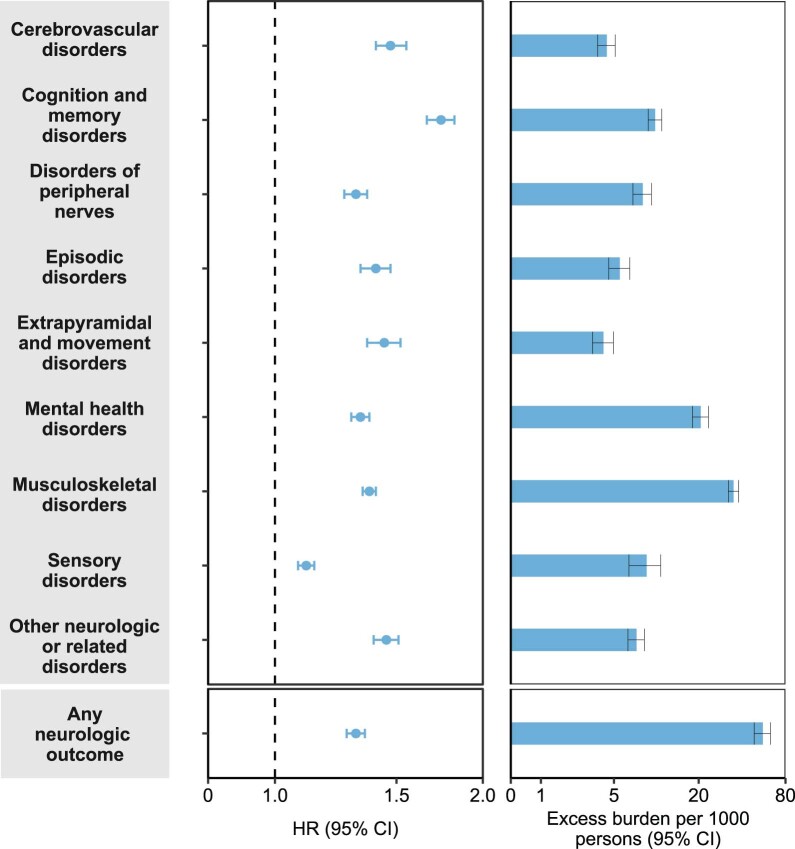
Risks and 12-month burdens of incident postacute COVID-19 composite neurologic outcomes compared with the historical control cohort. Composite outcomes consisted of cerebrovascular disorders (ischemic stroke, TIA, hemorrhagic stroke, and cerebral venous thrombosis), cognition and memory (memory problems and Alzheimer’s disease), disorders of the peripheral nerves (peripheral neuropathy, paresthesia, dysautonomia, and Bell’s palsy), episodic disorders (migraine, epilepsy and seizures, and headache disorders), extrapyramidal and movement disorders (abnormal involuntary movements, tremor, Parkinson-like disease, dystonia, myoclonus), mental health disorders (major depressive disorders, stress and adjustment disorders, anxiety disorders, and psychotic disorders), musculoskeletal disorders (joint pain, myalgia, and myopathy), sensory disorders (Hearing abnormalities or tinnitus, vision abnormalities, loss of smell, and loss of taste), other neurologic or related disorders (dizziness, somnolence, Guillain-Barré syndrome, encephalitis or encephalopathy and transverse myelitis), and any neurologic outcome (incident occurrence of any neurologic outcome studied). Outcomes were ascertained 30d after the COVID-19-positive test until the end of follow up. COVID-19 cohort (n = 154,068) and historical control cohort (n = 5,859,621). Adjusted HRs (dots) and 95% (error bars) CIs are presented, as are estimated excess burdens (bars) and 95% confidence intervals (error bars). Burdens are presented per 1000 persons at 12 months of follow up. The dashed line marks a HR of 1.00; lower limits of 95% CIs with values greater than 1.00 indicate significantly increased risk.

**Extended Data Fig. 6:**
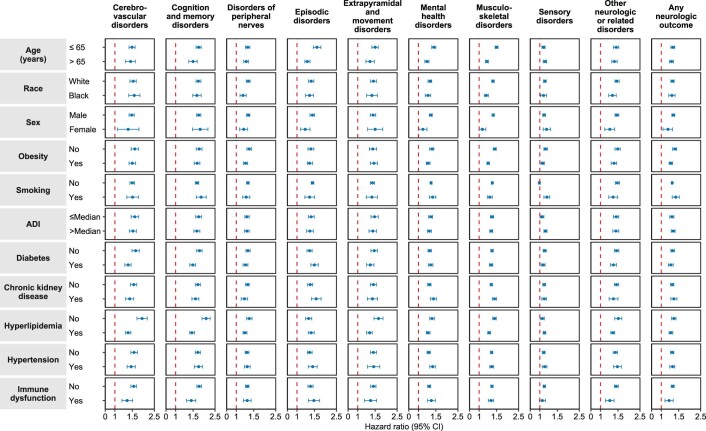
Subgroup analyses of the risks of incident postacute COVID-19 composite neurologic outcomes compared with the historical control cohort. Composite outcomes consisted of cerebrovascular disorders (ischemic stroke, TIA, hemorrhagic stroke, and cerebral venous thrombosis), cognition and memory (memory problems and Alzheimer’s disease), disorders of the peripheral nerves (peripheral neuropathy, paresthesia, dysautonomia, and Bell’s palsy), episodic disorders (migraine, epilepsy and seizures, and headache disorders), extrapyramidal and movement disorders (abnormal involuntary movements, tremor, Parkinson-like disease, dystonia, myoclonus), mental health disorders (major depressive disorders, stress and adjustment disorders, anxiety disorders, and psychotic disorders), musculoskeletal disorders (joint pain, myalgia, and myopathy), sensory disorders (Hearing abnormalities or tinnitus, vision abnormalities, loss of smell, and loss of taste), other neurologic or related disorders (dizziness, somnolence, Guillain-Barré syndrome, encephalitis or encephalopathy and transverse myelitis), and any neurologic outcome (incident occurrence of any neurologic outcome studied). Outcomes were ascertained 30d after the COVID-19-positive test until the end of follow up. COVID-19 cohort (n = 154,068) and historical control cohort (n = 5,859,621). Adjusted HRs (dots) and 95% (error bars) CIs are presented. The dashed line marks a HR of 1.00; lower limits of 95% CIs with values greater than 1.00 indicate significantly increased risk.

**Extended Data Fig. 7:**
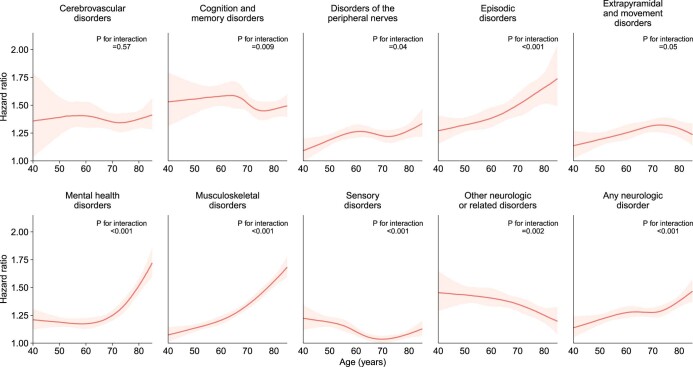
The risks of incident postacute COVID-19 composite neurologic outcomes across age compared with the historical control cohort. Composite outcomes consisted of cerebrovascular disorders (ischemic stroke, TIA, hemorrhagic stroke, and cerebral venous thrombosis), cognition and memory (memory problems and Alzheimer’s disease), disorders of the peripheral nerves (peripheral neuropathy, paresthesia, dysautonomia, and bell’s palsy), episodic disorders (migraine, epilepsy and seizures, and headache disorders), extrapyramidal and movement disorders (abnormal involuntary movements, tremor, Parkinson-like disease, dystonia, myoclonus), mental health disorders (major depressive disorders, stress and adjustment disorders, anxiety disorders, and psychotic disorders), musculoskeletal disorders (joint pain, myalgia, and myopathy), sensory disorders (Hearing abnormalities or tinnitus, vision abnormalities, loss of smell, and loss of taste), other neurologic or related disorders (dizziness, somnolence, Guillain-Barré syndrome, encephalitis or encephalopathy and transverse myelitis), and any neurologic outcome (incident occurrence of any neurologic outcome studied). Outcomes were ascertained 30d after the COVID-19-positive test until the end of follow up. COVID-19 cohort (n = 154,068) and historical control cohort (n = 5,859,621). Age was transformed into restricted cubic spline function for the analyses. P value was based on 2 sided Wald Chi-Squared test on interaction between age and exposure, without multiple comparisons adjustment. A P value of <0.05 suggests that age modifies the association between COVID-19 and the neurologic outcome. The P value for cerebrovascular disorders=0.57, cognition and memory disorders=0.009, disorders of the peripheral nerves=0.04, episodic disorders<0.001, extrapyramidal and movement disorders=0.05, mental health disorders<0.001, musculoskeletal disorders<0.001, sensory disorders<0.001, other neurologic or related disorders=0.002, and any neurologic disorder<0.001.

**Extended Data Fig. 8:**
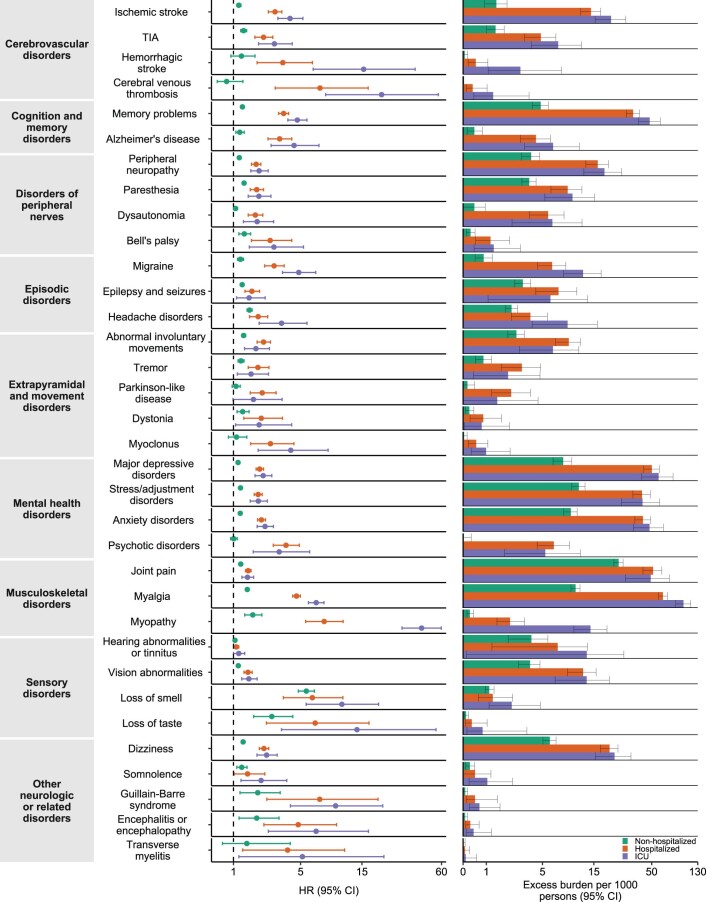
Risks and 12-month burdens of incident postacute COVID-19 neurologic outcomes by care setting of the acute infection compared with the historical control cohort. Risks and burdens were assessed at 12 months in mutually exclusive groups comprising nonhospitalized individuals with COVID-19 (green), individuals hospitalized for COVID-19 (orange) and individuals admitted to intensive care for COVID-19 during the acute phase (first 30 d) of COVID-19 (purple). Outcomes were ascertained 30 d after the COVID-19-positive test until the end of follow up. The historical control cohort served as the referent category. Within the COVID-19 cohort, nonhospitalized (n = 131,915), hospitalized (n = 16,764), admitted to intensive care (n = 5,389) and historical control cohort (n = 5,809,908). Adjusted HRs (dots) and 95% (error bars) CIs are presented, as are estimated excess burdens (bars) and 95% confidence intervals (error bars). Burdens are presented per 1000 persons at 12 months of follow up. The dashed line marks a HR of 1.00; lower limits of 95% CIs with values greater than 1.00 indicate significantly increased risk.

**Extended Data Fig. 9:**
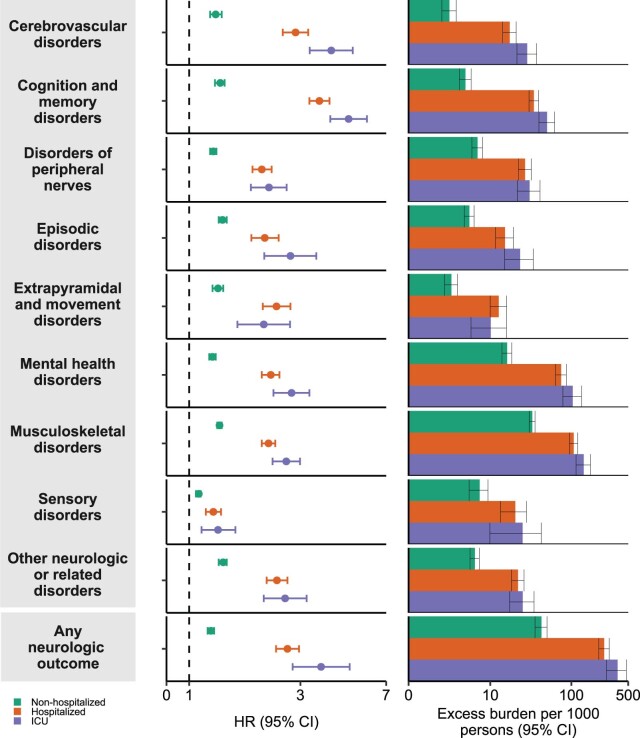
Risks and 12-month burdens of incident postacute COVID-19 composite neurologic outcomes by care setting of the acute infection compared with the historical control cohort. Risks and burdens were assessed at 12 months in mutually exclusive groups comprising nonhospitalized individuals with COVID-19 (green), individuals hospitalized for COVID-19 (orange) and individuals admitted to intensive care for COVID-19 during the acute phase (first 30 d) of COVID-19 (purple). Composite outcomes consisted of cerebrovascular disorders (ischemic stroke, TIA, hemorrhagic stroke, and cerebral venous thrombosis), cognition and memory (memory problems and Alzheimer’s disease), disorders of the peripheral nerves (peripheral neuropathy, paresthesia, dysautonomia, and Bell’s palsy), episodic disorders (migraine, epilepsy and seizures, and headache disorders), extrapyramidal and movement disorders (abnormal involuntary movements, tremor, Parkinson-like disease, dystonia, myoclonus), mental health disorders (major depressive disorders, stress and adjustment disorders, anxiety disorders, and psychotic disorders), musculoskeletal disorders (joint pain, myalgia, and myopathy), sensory disorders (Hearing abnormalities or tinnitus, vision abnormalities, loss of smell, and loss of taste), other neurologic or related disorders (dizziness, somnolence, Guillain-Barré syndrome, encephalitis or encephalopathy and transverse myelitis), and any neurologic outcome (incident occurrence of any neurologic outcome studied). Outcomes were ascertained 30 d after the COVID-19-positive test until the end of follow up. The historical control cohort served as the referent category. Within the COVID-19 cohort, nonhospitalized (n = 131,915), hospitalized (n = 16,764), admitted to intensive care (n = 5,389) and historical control cohort (n = 5,809,908). Adjusted HRs (dots) and 95% (error bars) CIs are presented, as are estimated excess burdens (bars) and 95% confidence intervals (error bars). Burdens are presented per 1000 persons at 12 months of follow up. The dashed line marks a HR of 1.00; lower limits of 95% CIs with values greater than 1.00 indicate significantly increased risk.

To test the robustness of study results, we evaluated the associations between COVID-19 and the prespecified neurologic outcomes in analyses considering a historical control group (from an era predating the pandemic) as the referent category; the demographic and health characteristics before and after weighting are presented in Supplementary Tables 1, 2, 8 and 9, examination of standardized mean differences suggested that covariates were balanced after application of inverse weighting (Extended Data Fig. 3b,c). The results showed increased risks and associated burdens of the prespecified outcomes in comparisons of COVID-19 versus the overall historical control group (Extended Data Figs. 4 and 5 and Supplementary Table 10), in subgroup analyses and by age as continuous variable (Extended Data Fig. 6 and 7 and Supplementary Table 11) and by care setting of the acute phase of the disease (Extended Data Figs 8 and 9 and Supplementary Table 12). Both the direction and magnitude of risks were consistent with analyses using the contemporary control as the referent category.

### Sensitivity analyses

We investigated the robustness of our results in multiple sensitivity analyses. We tested the association between COVID-19 and all the composite outcomes in sensitivity analyses involving comparisons between COVID-19 versus the contemporary control and—separately—COVID-19 versus the historical control, and additionally COVID-19 by care setting of the acute phase of the infection versus both controls. (1) We tested the results in models specified to include only predefined covariates (that is without inclusion of any algorithmically selected high-dimensional covariates) to build the inverse probability weighting; (2) we employed the doubly robust method through application of both weighting and covariate adjustment in the survival models (instead of the inverse weighting method used in primary analyses) as an alternative approach to examine the associations between COVID-19 and the risk of the prespecified neurologic outcomes. The results from the sensitivity analyses were consistent with those generated using the primary approach (Supplementary Tables 13a,b and14a,b).

### Positive- and negative-outcome controls

To verify whether our approach would reproduce established knowledge, we tested fatigue as a positive outcome control. The results suggested that COVID-19 was associated with increased risk of fatigue in comparison with the contemporary control and the historical control (Supplementary Table 15).

To test for potential presence of spurious biases, we subjected our analytic approach to the examination of a battery of three negative-outcome controls where no prior knowledge suggests an association is expected. The results showed no statistically significant association between COVID-19 and any of the negative-outcome controls in comparison with the contemporary and the historical control groups—these results were consistent with pretest expectations (Supplementary Table 15).

### Negative-exposure controls

To further test the rigor of our approach, we examined the associations between a pair of negative-exposure controls and each of our prespecified outcomes. We hypothesized that receipt of influenza vaccination in odd- versus even-numbered calendar days between 1 March 2020 and 15 January 2021 would be associated with similar risks of each of the prespecified neurologic outcomes evaluated in this analysis. We therefore tested the associations between receipt of the influenza vaccine in even- (*n* = 571,291) versus odd- (*n* = 605,453) numbered calendar days and each of the prespecified neurologic outcomes. We used the same data sources, cohort design, analytic approach (including covariate specification and weighting method) and the same set of prespecified outcomes. Consistent with our pretest expectations, the results showed that receipt of influenza vaccination in odd-numbered calendar days versus even-numbered calendar days was not significantly associated with any of the prespecified neurologic outcomes (Supplementary Table 16).

## Discussion

In this study involving 154,068 people who had COVID-19, 5,638,795 contemporary controls and 5,859,621 historical controls, which altogether correspond to 14,064,985 person-years of follow up, we show that beyond the first 30 days of infection, people with COVID-19 are at increased risk of an array of neurologic disorders spanning several disease categories including stroke (both ischemic and hemorrhagic), cognition and memory disorders, peripheral nervous system disorders, episodic disorders, extrapyramidal and movement disorders, mental health disorders, musculoskeletal disorders, sensory disorders and other disorders including Guillain–Barré syndrome, and encephalitis or encephalopathy. The risks and burdens were evident in subgroups based on age, race, sex, obesity, smoking, ADI, diabetes, chronic kidney disease, hyperlipidemia, hypertension or immune dysfunction. The risks were evident even in people who did not need hospitalization during the acute phase of the infection and increased according to the care setting of the acute phase of the disease from nonhospitalized to hospitalized to admitted to intensive care. The findings were consistent in comparisons involving the contemporary control group and the historical control group. The results were robust to challenge in sensitivity analyses; the application of negative-exposure and negative-outcome controls yielded results consistent with prior expectations. Altogether, our results show that the risks and burdens of neurologic disorders in the COVID-19 group at 12 months are substantial. The long-term consequences of SARS-CoV-2 infection should be taken into account in devising policies for managing the ongoing pandemic, and developing exit strategies for a postpandemic era. Health systems should consider these findings in capacity planning and in designing clinical care pathways to address the care needs of people who survive the acute phase of COVID-19.

More than 2 years into the COVID-19 global pandemic, it is abundantly clear that infection with SARS-CoV-2 may result in a broad array of long-term disorders^9–14^. Our report adds to this growing body of evidence by providing a comprehensive account of the neurologic consequences of COVID-19 at 12 months. Given the colossal scale of the pandemic, and even though the absolute numbers reported in this work are small, these may translate into a large number of affected individuals around the world—and this will likely contribute to a rise in the burden of neurologic diseases. This places more emphasis on the continued need for multipronged primary prevention strategies through nonpharmaceutical interventions (for example, masking) and vaccines to reduce—to the extent possible—the risk of contracting SARS-CoV-2. There is also an urgent need to develop long-term sustainable strategies to prevent mass infection with SARS-CoV-2 and to determine whether and how these long-term neurologic (and other) complications could be prevented or otherwise mitigated in people who are already infected with SARS-CoV-2.

Governments and health systems should take into account the findings that SARS-CoV-2 leads to long-term neurologic (and other serious) consequences when devising policy for continued management of this pandemic and developing plans for a postpandemic world. Some of the neurologic disorders reported here are serious chronic conditions that will impact some people for a lifetime. These conditions require early identification and care to reduce the risk of further downstream adverse outcomes. The added burden of new (incident) neurologic disease (and other incident long-term disorders) that result as a consequence of infection with SARS-CoV-2 will likely have profound ramifications not only on patients’ quality of life and life expectancy but also on health systems and economic productivity; these also risk widening inequities^15^. It is imperative that we recognize the enormous challenges posed by Long Covid and all its downstream long-term consequences. Meeting these challenges requires urgent and coordinated—but so far absent—global, national and regional response strategies^16,17^.

Our estimates of the risk of cerebrovascular disorders are generally consistent with our prior report (which was focused on investigating cardiovascular outcomes and included cerebrovascular disorders); minor differences in estimates of risk and burden are likely due to updated analytic approach and the longer follow up time (generally 60 more days of follow up in this current study)^18^.

Our analyses by age as a continuous variable reveal two key findings. (1) Regardless of age and across the age spectrum, people with COVID-19 had a higher risk of all the neurologic outcomes examined in this analysis. (2) Our interaction analyses suggest that the effect of COVID-19 on risk of memory and cognitive disorders, sensory disorders and other neurologic disorders (including Guillain–Barré syndrome and encephalitis or encephalopathy) is stronger in younger adults; the effects of these disorders on younger lives are profound and cannot be overstated; urgent attention is needed to better understand these long-term effects and the means to mitigate them. Equally troubling is the stronger effect of COVID-19 on mental health disorders, musculoskeletal disorders and episodic disorders in older adults, highlighting their vulnerability to these disorders following SARS-CoV-2 infection.

Several mechanisms have been proposed to explain the postacute sequelae of COVID-19; these include persistence of the virus, RNA fragments or viral proteins leading to continued activation of the immune system and chronic inflammation; other mechanisms may involve autoimmunity, microbiome dysbiosis and organ injury during the acute phase that may result in postacute manifestations^19–25^. The neurologic manifestations of Long Covid are hypothesized to be driven by neuro-inflammation with trafficking of immune cells (T cells and natural killer cells), cytokines and antibodies to the brain parenchyma resulting in activation of microglia and astrocytes, disturbances in synaptic signaling of upper-layer excitatory neurons, impaired neurogenesis and neuroblast formation, loss of oligodendrocytes and reduced myelinated axon density^22,23,26^. Other mechanisms may involve endothelial cell injury, complement activation and complement-mediated coagulopathy and microangiopathy leading to microbleeds or microclots^27–29^. Evidence from brain lysates of people with COVID-19 (compared with noninfected controls) demonstrates upregulation of transforming-growth-factor-beta signaling, hyperphosphorylation and posttranslational modification of receptor and channel proteins typically linked to Alzheimer’s disease^30^. Direct invasion of the virus into the central nervous system has also been proposed as a putative hypothetical mechanism of neuronal injury^22^. Evidence also suggests significant structural brain changes in the postacute phase of COVID-19; analyses of neuro-imaging data pre- and 4 to 5 months postinfection with SARS-CoV-2 reveal significant longitudinal effects—even in mild cases—including reduction in gray-matter thickness, increased activity of markers of tissue damage and reduction in global brain size^2^. Because of the broad nature of the neurologic sequelae of SARS-CoV-2, various—and not necessarily mutually exclusive—mechanisms may be at play for different neurologic disorders; these mechanisms may accelerate progression of pre-existing subclinical disease or result in de novo disease^31^.

This study has several strengths. We leveraged the breadth and depth of the national healthcare databases of the US Department of Veterans Affairs to build a large cohort of 154,068 people who had COVID-19 and more than 11 million people in the control group. We investigated a comprehensive list of prespecified neurologic outcomes. We used both predefined (based on established knowledge) and—in recognition of our incomplete and evolving knowledge of COVID-19—an expanded set of 100 algorithmically selected covariates in several data domains including diagnostic codes, prescription records and laboratory test results to balance the exposure groups and estimate the risk and burden of neurologic disorders at 12 months. We examined the associations in clinically important subgroups and across the spectrum of care during the acute phase of COVID-19 (nonhospitalized, hospitalized and admitted to intensive care). We investigated these associations in COVID-19 versus a contemporary cohort exposed to the broader contextual changes brought on by the pandemic, and a historical cohort from an era undisturbed by the pandemic. We subjected our analyses to the scrutiny of multiple sensitivity analyses and successfully demonstrated testing of negative-exposure and outcome controls. Finally, we provide two measures of risk: (1) hazard ratios on the relative scale; and (2) excess burden on absolute scale. The latter also incorporates the contribution of baseline risk and is useful to understand and contextualize the broader impact of the relative risk on the population.

This study has several limitations. The demographic characteristics of the study population (majority White and male) may limit generalizability of findings. Although we adjusted—through weighting—for predefined and algorithmically selected covariates, and although we used validated definitions for outcomes, and our results were robust to challenge in sensitivity analyses and survived the scrutinous application of negative controls, we cannot completely rule out misclassification bias or residual confounding. Our contemporary control included people who had no evidence of SARS-CoV-2 infection; it is possible that some people had an infection but were not tested for it; these people will have been enrolled in the control group; and if present in large numbers, this may bias the results toward the null and lead to underestimation of risk. While results from inverse probability weighting may be sensitive to different specifications of the weighting processes^32–35^, we triangulated several approaches to model specification in our sensitivity analyses and all yielded consistent results. Because we aimed to examine outcomes at 12 months, our cohorts were enrolled before 15 January 2021 (before SARS-CoV-2 vaccines were widely available in the US), and less than 1% of people in the COVID-19 group and contemporary control group were vaccinated before *T*_0_. Our subgroup analyses were designed to estimate the risk of outcomes in each subgroup, the strength of the association for any specific outcome may not be necessarily comparable across subgroups. Finally, the pandemic remains a highly dynamic global event; as new variants of SARS-CoV-2 emerge, as vaccine uptake improves, as therapeutics for acute COVID-19 (monoclonal antibodies, antiviral agents) become more available, it is possible that the epidemiology of the long-term sequelae of SARS-CoV-2 infection (including long-term neurologic sequelae) may also change over time^36^.

In conclusion, our report provides a comprehensive analysis of neurologic outcomes at 12 months. We show increased risk of an array of neurologic disorders spanning several neurologic disease categories including stroke (both ischemic and hemorrhagic), cognition and memory disorders, peripheral nervous system disorders, episodic disorders, extrapyramidal and movement disorders, mental health disorders, musculoskeletal disorders, sensory disorders, and other disorders including Guillain–Barré syndrome, and encephalitis or encephalopathy. The risks were evident in all examined subgroups and were evident even in people who were not hospitalized during the acute phase of the disease. Altogether, the findings call for attention to the long-term neurologic consequences of SARS-CoV-2 infection. Both healthcare system planning, and more broadly, public policy making, should take into account the long-term neurologic (and other) consequences of infection with SARS-CoV-2.

## Methods

### Ethics statement

This study was approved by the institutional review board of the VA St. Louis Health Care System, which granted a waiver of informed consent (protocol number 1606333).

### Setting

This study was conducted using the electronic healthcare databases of the US Department of Veterans Affairs. The VHA—a branch of the US Department of Veterans Affairs—operates the largest nationally integrated healthcare system within the US consisting of 1,255 healthcare facilities (including 170 VA healthcare systems and 1,074 outpatient sites). All veterans enrolled in the VHA have access to a comprehensive medical benefit package, including preventative and health maintenance, outpatient care, inpatient hospital care, prescriptions, mental healthcare, home healthcare, primary care, specialty care, geriatric and extended care, medical equipment and prosthetics. VA electronic healthcare databases are updated daily.

### Cohort

A flowchart of cohort construction is provided in Fig. 1. Veterans who were users of the VHA in 2019 (*n* = 6,244,069) and had a positive COVID-19 test between 1 March 2020 and 15 January 2021 were selected for the COVID-19 cohort (*n* = 169,476). To facilitate the examination of postacute COVID-19 outcomes, we further selected those who were alive 30 days after the positive test result from the COVID-19 cohort (*n* = 154,068). The date of the first COVID-19 positive test served as *T*_0_ and marked the start of follow up; follow up ended on 31 December 2021.

We then constructed a contemporary control group consisting of veterans who were users of the VHA in 2019 (*n* = 6,244,069). Those who were alive by 1 March 2020 (*n* = 5,963,205) and were not already part of the COVID-19 cohort were selected for the contemporary control cohort (*n* = 5,809,137). The start of follow up of participants in the contemporary control cohort was randomly assigned following the same distribution of the date of a positive COVID-19 test result in the COVID-19 group so that the proportion of participants with a start of follow up on a certain date was the same in both groups; this ensures a similar distribution of follow-up time between the COVID-19 and contemporary control cohorts. At the start of follow up, 5,660,999 participants were alive. Those alive after 30 days after the start of follow up (*n* = 5,638,795) were selected as the contemporary control cohort. Follow up ended on 31 December 2021.

We also constructed a historical control group composed of 6,463,487 participants who were users of the VHA in 2017. From the 6,152,185 participants who were alive on 1 March 2018, 6,009,794 participants who were not already part of the COVID-19 group were enrolled into the historical control group. We randomly assigned *T*_0_ in the historical control group using the follow-up distribution of *T*_0_ in the COVID-19 group minus 2 years (730 days); this ensured a similar distribution of follow-up time between the COVID-19 and historical control cohorts. Overall, 5,876,880 participants in the historical control group were alive at *T*_0_; the final historical control group consisted of 5,859,621 participants that were alive 30 days after *T*_0_. End of follow up for the historical control group was set as 31 December 2019.

### Data sources

This study used electronic health records from the VA Corporate Data Warehouse (CDW). The CDW Patient domain provided patient demographic information. Outpatient clinical information was collected from the CDW Outpatient Encounters domain; clinical information during hospitalization was obtained from the CDW Inpatient Encounters domain. The CDW Outpatient Pharmacy and CDW Barcode Medication Administration domains provided information about medication prescriptions and fillings. Laboratory test information was gathered from the CDW Laboratory Results domain, and the COVID-19 Shared Data Resource provided information relevant to COVID-19. We also used the ADI, a summary measure of income, education, employment and housing, as a composite variable of contextual factors present at a participant’s residential location^37^.

### Prespecified outcomes

The prespecified outcomes were selected based on our earlier work on the systematic characterization of Long Covid^1,10,14^ and evidence from previous literature^38–44^. Each neurologic outcome was defined, based on the corresponding *International Classification of Diseases, 10th revision* (ICD10) diagnostic codes^1,9–12,38–44^. Codes are available on GitHub. Individual outcomes were also aggregated into a related composite outcome (for example, cerebrovascular disorders consisted of an aggregation of ischemic stroke, hemorrhagic stroke, cerebral venous thrombosis and TIAs). Additionally, we specified the composite of any neurologic outcome defined as the first incident occurrence of any of the predefined neurologic outcomes examined in this study. Incident individual and composite neurologic outcomes during the postacute phase of COVID-19 were assessed during the follow-up period between the 30 days after *T*_0_ until the end of follow up in those without any history of the specified outcome in the year before *T*_0_.

### Covariates

In recognition that our knowledge of COVID-19 is evolving and incomplete, we used a dual-pronged approach to identify covariates: (1) we selected covariates based on previous knowledge^1,3,5–13,22,36,45–49^; (2) we used an algorithmic approach to identify covariates in several data domains including diagnoses, medications and laboratory test results. Both predefined and algorithmically selected covariates were used in the modeling and were assessed in the year before *T*_0_.

Predefined covariates included age, race (White, Black and other), sex, ADI, body mass index, smoking status (current, former and never) and measures of healthcare utilization (number of outpatient and inpatient encounters as well as long-term care utilization)^1,36^. Several comorbidities were also selected as predefined variables, including cancer, chronic kidney disease, chronic lung disease, diabetes and hypertension. Additional covariates included estimated glomerular filtration rate and systolic and diastolic pressure. Continuous variables were transformed into restricted cubic spline function to account for potential nonlinear relationships.

Our predefined covariates were also supplemented by algorithmically selected covariates from high-dimensional data domains including diagnoses, medications and laboratory test results^50^. All information used for algorithmically selected covariates was collected within 1 year before the exposure. This was achieved by gathering all patient encounter, prescription and laboratory data and categorizing the information into 540 diagnostic groups, 543 medication types and 62 laboratory test abnormalities. We selected variables from these data domains (diagnoses, medications and laboratory test results) which occurred in at least 100 participants within each of the exposure groups—this was done in recognition that variables that are exceedingly rare (occur in less than 100 participants in these large cohorts) may not materially influence the examined associations. We then estimated the univariate relative risk between each variable and the exposure. The top 100 variables with the highest relative risk were selected^51^. This algorithmic selection process for high-dimensional covariates was conducted independently for each outcome-specific cohort.

### Statistical analyses

Baseline characteristics in the COVID-19, contemporary and historical control groups and standardized mean differences were described.

We estimated the risk of each incident neurologic outcome by first building a subcohort of participants without a history of the outcome of interest (for example, the risk of incident stroke was estimated within a subcohort of participants without history of stroke in the year before cohort enrollment). For each subcohort, multinomial logistic regression was built to estimate the probability of a participant belonging to the observed group (COVID-19, contemporary control and historical control group) conditional on all predefined covariates listed in the covariate section and algorithmically selected high-dimensional variables denoted by *L* (ref. ^52^). The estimated probability (*P*(group = observed group|*L*)) was used as the propensity score to calculate the inverse probability weight for average treatment effect within the cohort. The stabilized inverse probability weight was computed as *P*(group = observed group)/*P*(group = observed group|*L*), where *L* is the covariates, *P*(group = observed group) is the group proportion within the cohort and served as the stabilization factor^53^. To further reduce the influence of extreme weights, the stabilized weights were truncated at 30 (refs. ^32,33,35^). Less than 0.001% of the stabilized weights were greater than 30 and were truncated. After application of weighting, covariate balance was assessed by standardized mean differences.

We then used cause-specific hazard models where death was considered as a competing risk to estimate hazard ratios of incident neurologic outcomes between the COVID-19 and contemporary cohorts and the COVID-19 and historical cohorts after application of inverse probability weights. The burdens per 1,000 participants at 12 months of follow up in the COVID-19 and control groups were estimated based on the survival probability at 12 months within each group; excess burdens were computed based on the difference of the estimated burdens between COVID-19 and control groups. Additionally, we conducted analyses in subgroups based on participant age, race, sex, obesity, smoking, ADI, diabetes, chronic kidney disease, hyperlipidemia, hypertension and immune dysfunction statuses. To further understand the association between COVID-19 and incident neurologic outcomes across age, we conducted spline analyses, where age was treated as restricted cubic spline with knots placed at the 10th, 35th, 65th and 90th percentiles. We also performed interaction analyses between age and COVID-19 exposure to examine whether age modified the association between COVID-19 and outcomes.

The association between COVID-19 and risks of postacute neurologic outcomes was evaluated in mutually exclusive groups based on participants’ care setting during the acute phase of COVID-19 infection (that is, whether participants were nonhospitalized, hospitalized or admitted into the intensive care unit during the first 30 days of infection). Using the approach outlined in the previous paragraph, inverse probability weights were estimated for each care setting group. Cause-specific hazard models with inverse probability weighting were applied, and HRs, burdens and excess burdens were calculated.

To further test the robustness of our study design, we conducted multiple sensitivity analyses. (1) We modified our covariate selection by restricting covariate inclusion to only predefined variables when constructing the inverse probability weight (that is, we did not include any algorithmically selected covariates). (2) Alternatively, we applied a doubly robust approach, in which associations were estimated by applying both covariates adjustment and the inverse probability weights to survival models^14^.

To examine whether our approach would reproduce known associations, we tested the outcome of fatigue—a signature sequela of Long Covid—as a positive outcome control. To further test the rigor of our approach, we tested a battery of negative-outcome controls, for which no prior evidence supports the existence of a causal relationship between COVID-19 exposure and any of these negative-outcome controls^53^. We also tested a pair of negative-exposure controls. We hypothesized that exposure to the influenza vaccine in odd-numbered or even-numbered calendar days between 1 March 2020 and 15 January 2021 would be associated with similar risks of all the neurologic outcomes examined in our analyses. Successful application of these negative outcomes and negative-exposure controls might reduce concern about the presence of spurious biases related to study design, covariate selection, analytic approach, outcome ascertainment, residual confounding and other potential sources of latent biases^53^.

Robust sandwich variance estimators were used to provide an estimation of variance when applying weightings. In all analyses, evidence of statistical significance was considered when a 95% CI excluded unity. All analyses were conducted using SAS Enterprise Guide v.8.2 (SAS Institute), and visualization of results was accomplished using R v.4.04.

### Reporting summary

Further information on research design is available in the Nature Research Reporting Summary linked to this article.

## Online content

Any methods, additional references, Nature Research reporting summaries, source data, extended data, supplementary information, acknowledgments, peer review information; details of author contributions and competing interests; and statements of data and code availability are available at 10.1038/s41591-022-02001-z.

## Supplementary information

Supplementary Information Supplementary Table of Contents and Tables 1–16.

Reporting Summary

## Data Availability

The data that support the findings of this study are available from the US Department of Veterans Affairs. VA data are made freely available to researchers behind the VA firewall with an approved VA study protocol. For more information, please visit https://www.virec.research.va.gov or contact the VA Information Resource Center (VIReC) at VIReC@va.gov.

## Extended data

are available for this paper at 10.1038/s41591-022-02001-z.

## Code availability

The analytic code is available at https://github.com/yxie618/longNeurologic.
