# Supplementary material for: Long-term neurologic outcomes of COVID-19
Source: Nat Med. 2022 Sep 22;28(11):2406–15. doi: 10.1038/s41591-022-02001-z (PMC9671811; doi:10.1038/s41591-022-02001-z)
Supplement: Supplementary file 1 — Supplementary Table of Contents and Tables 1–16. [file 41591_2022_2001_MOESM1_ESM.pdf]

nature portfolio

https://doi.org/10.1038/s41591-022-02001-z

Supplementary information

Long-term neurologic outcomes of COVID-19

In the format provided by the authors and unedited

Long-term Neurologic Outcomes of COVID-19

Table of Contents	Page
**SUPPLEMENTARY TABLES**	
Supplementary Table 1. Demographic and health characteristics of COVID-19, contemporary and historical cohorts before weighting	3-4
Supplementary Table 2. Demographic and health characteristics of COVID-19, contemporary and historical cohorts after weighting	5-6
Supplementary Table 3. Risks and 12-month burdens of post-acute COVID-19 neurologic outcomes compared to contemporary control	7-8
Supplementary Table 4. Subgroup analyses of the risks of incident post-acute COVID-19 composite neurologic outcomes compared to contemporary control	9-10
Supplementary Table 5. Demographic and health characteristics of the COVID-19 and contemporary cohorts by care setting of the acute infection before weighting	11-12
Supplementary Table 6. Demographic and health characteristics of the COVID-19 and contemporary cohorts by care setting of the acute infection after weighting	13-14
Supplementary Table 7. Risks and 12-month burdens of post-acute COVID-19 neurologic outcomes by care setting of the acute infection compared to contemporary control	15-19
Supplementary Table 8. Demographic and health characteristics of the COVID-19 and historical cohorts by care setting of the acute infection before weighting	20-21
Supplementary Table 9. Demographic and health characteristics of the COVID-19 and historical cohorts by care setting of the acute infection after weighting	22-23
Supplementary Table 10. Risks and 12-month burdens of post-acute COVID-19 neurologic outcomes compared to historical control	24-25
Supplementary Table 11. Subgroup analyses of the risks of incident post-acute COVID-19 composite neurologic outcomes compared to historical control	26-27
Supplementary Table 12. Risks and 12-month burdens of post-acute COVID-19 neurologic outcomes by care setting of the acute infection compared to historical control	28-32
Supplementary Table 13a. Sensitivity analysis for composite outcomes compared to the contemporary control	33
Supplementary Table 13b. Sensitivity analysis for composite outcomes compared to the historical control	34

Supplementary Table 14a. Sensitivity analysis for composite outcomes by care setting of the acute infection compared to the contemporary control	35
Supplementary Table 14b. Sensitivity analysis for composite outcomes by care setting of the acute infection compared to the historical control	36
Supplementary Table 15. Positive and negative outcome controls	37
Supplementary Table 16. Negative exposure control: risks and 12-month burdens of neurologic outcomes of those vaccinated for influenza on even-numbered days compared to those vaccinated on odd-numbered days	38-39

Supplementary Table 1. Demographic and health characteristics of COVID-19, contemporary and historical cohorts before weighting

Baseline Characteristics	COVID-19 (N=154,068)	Contemporary control (N=5,638,795)	Historical control (N=5,859,621)	Absolute standardized difference[†]	
				COVID-19 and Contemporary control	COVID-19 and Historical control
**Age, mean (std), yr**	61.42 (15.64)	63.46 (16.23)	62.89 (16.48)	0.13	0.09
**Race, no. (%)**					
White	109,226 (70.89)	4,329,617 (76.78)	4,532,627 (77.35)	0.13	0.15
Black	37,159 (24.12)	1,042,397 (18.49)	1,051,655 (17.95)	0.14	0.15
Other	7,683 (4.99)	266,781 (4.73)	275,339 (4.70)	0.01	0.01
**Sex, no. (%)**					
Male	137,179 (89.04)	5,092,461 (90.31)	5,308,943 (90.60)	0.04	0.05
Female	16,889 (10.96)	546,334 (9.69)	550,678 (9.40)	0.04	0.05
**BMI category, no. (%), kg/m^2**					
Underweight/Normal	21,456 (13.93)	1,062,237 (18.84)	1,123,533 (19.17)	0.13	0.14
Overweight	49,702 (32.26)	2,190,851 (38.85)	2,332,370 (39.80)	0.14	0.16
Obese	82,910 (53.81)	2,385,707 (42.31)	2,403,718 (41.02)	0.23	0.26
**Smoking status, no. (%)**					
Never	69,208 (44.92)	2,397,935 (42.53)	3,084,196 (52.63)	0.05	0.15
Former	61,229 (39.74)	2,032,158 (36.04)	1,441,928 (24.61)	0.08	0.33
Current	23,631 (15.34)	1,208,702 (21.44)	1,333,497 (22.76)	0.16	0.19
**Area Deprivation Index[a], mean (std)**	55.35 (18.57)	54.67 (19.03)	54.65 (19.12)	0.04	0.04
Clinical Characteristics					
**Outpatient encounter*, no. (%)**					
Zero	4,174 (2.71)	591,209 (10.48)	551,102 (9.41)	0.32	0.28
One	26,585 (17.26)	1,853,280 (32.87)	1,868,338 (31.89)	0.37	0.34
Two or more	123,309 (80.04)	3,194,306 (56.65)	3,440,181 (58.71)	0.52	0.48
**Long-term care, no. (%)**	6,115 (3.97)	33,593 (0.60)	47,598 (0.81)	0.23	0.21
**eGFR, mean (std), ml/min/1.73m^2**	77.75 (22.50)	78.59 (20.08)	79.32 (19.95)	0.04	0.07

Baseline Characteristics	COVID-19 (N=154,068)	Contemporary control (N=5,638,795)	Historical control (N=5,859,621)	Absolute standardized difference[†]	
				COVID-19 and Contemporary control	COVID-19 and Historical control
**Systolic blood pressure, mean (std), mmHg**	132.65 (11.74)	132.65 (12.29)	132.63 (12.63)	0.00	0.00
**Diastolic blood pressure, mean (std), mmHg**	78.32 (7.41)	77.78 (7.54)	77.53 (7.91)	0.07	0.10
**Cancer, no. (%)**	12,100 (7.85)	324,306 (5.75)	341,895 (5.83)	0.08	0.08
**Chronic kidney disease, no. (%)**	30,054 (19.51)	939,892 (16.67)	901,162 (15.38)	0.07	0.11
**Chronic lung disease, no. (%)**	22,614 (14.68)	573,194 (10.17)	646,148 (11.03)	0.14	0.11
**Diabetes mellitus type 2, no. (%)**	47,566 (30.87)	1,200,771 (21.29)	1,322,647 (22.57)	0.22	0.19
**Hyperlipidemia, no. (%)**	90,874 (58.98)	2,578,731 (45.73)	2,981,020 (50.87)	0.27	0.16
**Hypertension, no. (%)**	41,142 (26.70)	1,484,451 (26.33)	1,539,549 (26.27)	0.01	0.01

[†]. Standardized difference less than 0.15 is considered good balance.
[a].Area Deprivation Index is a measure of socioeconomic disadvantage, with a range from low to high disadvantage of 0 to 100.
*. Data collected within one year of cohort enrollment
std, standard deviation; BMI, body mass index; eGFR, estimated glomerular filtration rate.

Supplementary Table 2. Demographic and health characteristics of COVID-19, contemporary and historical cohorts after weighting

Baseline Characteristics	COVID-19 (N=154,068)	Contemporary control (N=5,638,795)	Historical control (N=5,859,621)	Absolute standardized difference†	
				COVID-19 and Contemporary control	COVID-19 and Historical control
**Age, mean (std), yr**	63.02 (16.13)	63.14 (16.28)	63.19 (16.38)	0.01	0.01
**Race, no. (%)**					
White	117,674 (76.38)	4,338,771 (76.95)	4,509,799 (76.96)	0.01	0.01
Black	29,070 (18.87)	1,032,463 (18.31)	1,071,666 (18.29)	0.01	0.01
Other	7,324 (4.75)	267,561 (4.75)	278,156 (4.75)	0.00	0.00
**Sex, no. (%)**					
Male	138,108 (89.64)	5,095,892 (90.37)	5,296,277 (90.39)	0.02	0.02
Female	15,960 (10.36)	542,903 (9.63)	563,344 (9.61)	0.02	0.02
**BMI category, no. (%), kg/m^2**					
Underweight/Normal	29,757 (19.31)	1,073,683 (19.04)	1,104,890 (18.86)	0.01	0.01
Overweight	56,147 (36.44)	2,182,270 (38.70)	2,331,543 (39.79)	0.05	0.07
Obese	68,164 (44.24)	2,382,842 (42.26)	2,423,188 (41.35)	0.04	0.06
**Smoking status, no. (%)**					
Never	67,741 (43.97)	2,696,866 (47.83)	2,803,477 (47.84)	0.08	0.08
Former	49,240 (31.96)	1,704,946 (30.24)	1,770,426 (30.21)	0.04	0.04
Current	37,087 (24.07)	1,236,926 (21.94)	1,285,718 (21.94)	0.05	0.05
**Area Deprivation Index[a], mean (std)**	55.19 (19.00)	54.66 (19.08)	54.66 (19.07)	0.03	0.03
Clinical Characteristics					
**Outpatient encounter*, no. (%)**					
Zero	20,600 (13.37)	897,358 (15.91)	908,124 (15.50)	0.07	0.06
One	38,918 (25.26)	1,580,385 (28.03)	1,677,141 (28.62)	0.06	0.08
Two or more	94,548 (61.37)	3,161,052 (56.06)	3,274,356 (55.88)	0.11	0.11
**Long-term care, no. (%)**	2,431 (1.58)	44,039 (0.78)	45,646 (0.78)	0.07	0.07
**eGFR, mean (std), $ml/min/1.73m^2$**	78.87 (20.83)	78.94 (20.04)	78.90 (20.17)	0.00	0.00

Baseline Characteristics	COVID-19 (N=154,068)	Contemporary control (N=5,638,795)	Historical control (N=5,859,621)	Absolute standardized difference†	
				COVID-19 and Contemporary control	COVID-19 and Historical control
**Systolic blood pressure, mean (std), mmHg**	132.44 (12.51)	132.63 (12.41)	132.63 (12.46)	0.02	0.02
**Diastolic blood pressure, mean (std), mmHg**	77.55 (7.74)	77.66 (7.70)	77.65 (7.74)	0.01	0.01
**Cancer, no. (%)**	10,594 (6.88)	343,685 (6.10)	329,897 (5.63)	0.03	0.05
**Chronic kidney disease, no. (%)**	26,219 (17.02)	905,647 (16.06)	945,626 (16.14)	0.03	0.02
**Chronic lung disease, no. (%)**	20,295 (13.17)	603,295 (10.70)	628,444 (10.73)	0.08	0.08
**Diabetes mellitus type 2, no. (%)**	36,417 (23.64)	1,247,020 (22.12)	1,301,012 (22.20)	0.04	0.03
**Hyperlipidemia, no. (%)**	79,786 (51.79)	2,743,274 (48.65)	2,861,077 (48.83)	0.06	0.06
**Hypertension, no. (%)**	40,847 (26.51)	1,478,605 (26.22)	1,537,506 (26.24)	0.01	0.01

†. Standardized difference less than 0.15 is considered good balance.
[a].Area Deprivation Index is a measure of socioeconomic disadvantage, with a range from low to high disadvantage of 0 to 100.
*. Data collected within one year of cohort enrollment
std, standard deviation; BMI, body mass index; eGFR, estimated glomerular filtration rate.

Supplementary Table 3. Risks and 12-month burdens of post-acute COVID-19 neurologic outcomes compared to contemporary control

Outcome*	Hazard Ratio (95% CI)† COVID-19 vs Contemporary control	COVID-19 burden per 1000 persons at 12 months (95% CI) †	Contemporary control burden per 1000 persons at 12 months (95% CI) †	Absolute burden difference per 1000 persons at 12 months (95% CI)†
**Cerebrovascular disorders**	1.56 (1.48, 1.64)	13.82 (13.15, 14.52)	8.89 (8.82, 8.97)	4.92 (4.26, 5.62)
Ischemic stroke	1.50 (1.41, 1.61)	10.20 (9.55, 10.90)	6.80 (6.73, 6.87)	3.40 (2.75, 4.09)
TIA	1.62 (1.50, 1.75)	5.32 (4.92, 5.74)	3.28 (3.24, 3.33)	2.03 (1.64, 2.46)
Hemorrhagic stroke	2.19 (1.63, 2.95)	0.39 (0.29, 0.53)	0.18 (0.17, 0.19)	0.21 (0.11, 0.35)
Cerebral venous thrombosis	2.69 (1.29, 5.62)	0.08 (0.04, 0.18)	0.03 (0.03, 0.04)	0.05 (0.01, 0.14)
**Cognition and memory disorders**	1.80 (1.71, 1.88)	23.48 (22.40, 24.61)	13.13 (13.04, 13.23)	10.35 (9.27, 11.47)
Memory problems	1.77 (1.68, 1.85)	23.37 (22.29, 24.50)	13.30 (13.21, 13.39)	10.07 (9.00, 11.20)
Alzheimer’s disease	2.03 (1.79, 2.31)	3.26 (2.87, 3.70)	1.60 (1.57, 1.63)	1.65 (1.27, 2.10)
**Disorders of peripheral nerves**	1.34 (1.29, 1.39)	34.58 (33.38, 35.81)	25.94 (25.81, 26.07)	8.64 (7.44, 9.87)
Peripheral neuropathy	1.34 (1.28, 1.40)	22.49 (21.52, 23.50)	16.85 (16.75, 16.96)	5.64 (4.67, 6.65)
Paresthesia	1.32 (1.25, 1.39)	11.98 (11.36, 12.64)	9.09 (9.01, 9.17)	2.89 (2.27, 3.55)
Dysautonomia	1.30 (1.21, 1.40)	6.96 (6.48, 7.48)	5.36 (5.31, 5.42)	1.60 (1.12, 2.12)
Bell’s palsy	1.48 (1.24, 1.77)	0.97 (0.82, 1.16)	0.66 (0.64, 0.68)	0.32 (0.16, 0.51)
**Episodic disorders**	1.32 (1.26, 1.39)	19.56 (18.60, 20.57)	14.81 (14.71, 14.91)	4.75 (3.79, 5.76)
Migraine disorders	1.21 (1.14, 1.28)	12.00 (11.32, 12.72)	9.96 (9.88, 10.04)	2.04 (1.36, 2.76)
Epilepsy and seizures	1.80 (1.61, 2.01)	5.25 (4.70, 5.86)	3.23 (3.19, 3.28)	2.01 (1.47, 2.63)
Headache disorders	1.35 (1.25, 1.45)	5.69 (5.29, 6.12)	4.23 (4.18, 4.29)	1.46 (1.06, 1.89)
**Extrapyramidal and movement disorders**	1.42 (1.34, 1.50)	13.64 (12.90, 14.43)	9.66 (9.58, 9.74)	3.98 (3.24, 4.77)
Abnormal involuntary movements	1.41 (1.32, 1.50)	9.81 (9.21, 10.45)	6.96 (6.90, 7.03)	2.85 (2.24, 3.49)
Tremor	1.37 (1.25, 1.51)	4.08 (3.71, 4.49)	2.98 (2.94, 3.02)	1.10 (0.73, 1.51)
Parkinson-like disease	1.50 (1.28, 1.75)	2.68 (2.29, 3.13)	1.79 (1.76, 1.83)	0.89 (0.50, 1.34)
Dystonia	1.57 (1.29, 1.90)	1.10 (0.91, 1.34)	0.70 (0.68, 0.72)	0.40 (0.21, 0.63)
Myoclonus	1.42 (1.13, 1.79)	0.47 (0.37, 0.59)	0.33 (0.31, 0.34)	0.14 (0.04, 0.26)
**Mental health disorders**	1.43 (1.38, 1.47)	86.25 (83.64, 88.94)	61.25 (61.01, 61.49)	25.00 (22.40, 27.69)
Major depressive disorders	1.44 (1.39, 1.48)	58.18 (56.33, 60.09)	40.91 (40.73, 41.08)	17.28 (15.43, 19.18)
Stress and adjustment disorders	1.39 (1.34, 1.44)	52.02 (50.35, 53.75)	37.68 (37.51, 37.86)	14.34 (12.66, 16.07)

Outcome*	Hazard Ratio (95% CI)† COVID-19 vs Contemporary control	COVID-19 burden per 1000 persons at 12 months (95% CI)†	Contemporary control burden per 1000 persons at 12 months (95% CI)†	Absolute burden difference per 1000 persons at 12 months (95% CI)†
Anxiety disorders	1.38 (1.33, 1.42)	46.29 (44.79, 47.85)	33.86 (33.70, 34.02)	12.44 (10.93, 13.99)
Psychotic disorders	1.51 (1.33, 1.71)	3.03 (2.68, 3.44)	2.01 (1.97, 2.05)	1.02 (0.66, 1.43)
**Musculoskeletal disorders**	1.45 (1.42, 1.48)	136.32 (133.45, 139.25)	96.23 (95.95, 96.52)	40.09 (37.22, 43.01)
Joint pain	1.34 (1.31, 1.38)	113.43 (110.79, 116.13)	85.78 (85.52, 86.05)	27.65 (25.01, 30.35)
Myalgia	1.83 (1.77, 1.90)	35.52 (34.30, 36.78)	19.54 (19.43, 19.66)	15.97 (14.75, 17.23)
Myopathy	2.76 (2.30, 3.32)	1.11 (0.92, 1.33)	0.40 (0.38, 0.42)	0.71 (0.52, 0.93)
**Sensory disorders**	1.25 (1.22, 1.28)	88.07 (85.90, 90.30)	71.05 (70.81, 71.29)	17.03 (14.85, 19.26)
Hearing abnormalities and tinnitus	1.22 (1.18, 1.25)	68.84 (67.02, 70.71)	56.97 (56.76, 57.18)	11.87 (10.05, 13.75)
Vision abnormalities	1.30 (1.24, 1.36)	24.65 (23.60, 25.73)	19.05 (18.94, 19.17)	5.59 (4.55, 6.68)
Loss of smell	4.05 (3.45, 4.75)	1.42 (1.21, 1.67)	0.35 (0.34, 0.37)	1.07 (0.86, 1.32)
Loss of taste	2.26 (1.54, 3.32)	0.20 (0.14, 0.30)	0.09 (0.08, 0.10)	0.11 (0.05, 0.21)
**Other neurologic or related disorders**	1.46 (1.40, 1.52)	23.68 (22.71, 24.68)	16.31 (16.20, 16.41)	7.37 (6.41, 8.38)
Dizziness	1.44 (1.38, 1.50)	22.06 (21.14, 23.02)	15.41 (15.31, 15.51)	6.65 (5.72, 7.61)
Somnolence	1.67 (1.31, 2.12)	1.40 (1.10, 1.78)	0.84 (0.81, 0.86)	0.56 (0.26, 0.94)
Guillain-Barre syndrome	2.16 (1.40, 3.35)	0.20 (0.13, 0.31)	0.09 (0.08, 0.10)	0.11 (0.04, 0.22)
Encephalitis or encephalopathy	1.82 (1.16, 2.84)	0.16 (0.10, 0.25)	0.09 (0.08, 0.10)	0.07 (0.01, 0.16)
Transverse myelitis	1.49 (1.11, 2.00)	0.05 (0.02, 0.13)	0.02 (0.02, 0.03)	0.03 (0.00, 0.11)
**Any neurologic outcome**	1.42 (1.38, 1.47)	265.88 (258.73, 273.20)	195.19 (194.61, 195.77)	70.69 (63.54, 78.01)

*. Outcomes were ascertained from day 30 after the initial positive COVID-19 test result until end of follow up
†. Adjustment through inverse probability weighting using predefined and algorithmically selected high-dimensional variables.
CI, confidence interval; TIA, transient ischemic attack.

Supplementary Table 4. Subgroup analyses of the risks of incident post-acute COVID-19 composite neurologic outcomes compared to contemporary control

Risk Factors	COVID-19 vs Contemporary Control – Hazard Ratio (95%CI)*									
	Cerebro-vascular disorders	Cognition and memory disorders	Disorders of peripheral nerves	Episodic disorders	Extra-pyramidal and movement disorders	Mental health disorders	Musculo-skeletal disorders	Sensory disorders	Other neurologic or related disorders	Any neurologic outcome
**Age**										
≤65	1.60 (1.49, 1.71)	1.76 (1.66, 1.87)	1.32 (1.25, 1.39)	1.50 (1.37, 1.64)	1.44 (1.34, 1.55)	1.53 (1.46, 1.61)	1.53 (1.48, 1.58)	1.21 (1.17, 1.25)	1.45 (1.36, 1.54)	1.41 (1.35, 1.48)
>65	1.53 (1.36, 1.71)	1.67 (1.52, 1.84)	1.31 (1.24, 1.38)	1.22 (1.15, 1.29)	1.37 (1.24, 1.51)	1.31 (1.25, 1.38)	1.31 (1.27, 1.36)	1.31 (1.26, 1.37)	1.42 (1.34, 1.51)	1.41 (1.34, 1.47)
**Race**										
White	1.61 (1.50, 1.73)	1.75 (1.66, 1.86)	1.35 (1.29, 1.41)	1.31 (1.23, 1.39)	1.40 (1.31, 1.50)	1.41 (1.36, 1.47)	1.45 (1.41, 1.49)	1.25 (1.21, 1.29)	1.46 (1.39, 1.53)	1.41 (1.36, 1.46)
Black	1.70 (1.49, 1.94)	1.77 (1.61, 1.95)	1.21 (1.13, 1.31)	1.30 (1.19, 1.43)	1.44 (1.27, 1.63)	1.34 (1.26, 1.43)	1.30 (1.24, 1.37)	1.28 (1.20, 1.37)	1.39 (1.27, 1.52)	1.42 (1.32, 1.53)
**Sex**										
Male	1.57 (1.48, 1.66)	1.77 (1.69, 1.86)	1.35 (1.30, 1.40)	1.34 (1.26, 1.42)	1.40 (1.32, 1.49)	1.44 (1.39, 1.49)	1.47 (1.43, 1.50)	1.25 (1.21, 1.28)	1.47 (1.40, 1.53)	1.44 (1.39, 1.49)
Female	1.41 (1.10, 1.81)	1.73 (1.44, 2.08)	1.21 (1.10, 1.33)	1.21 (1.09, 1.35)	1.46 (1.23, 1.74)	1.21 (1.10, 1.32)	1.19 (1.10, 1.28)	1.24 (1.14, 1.35)	1.27 (1.13, 1.43)	1.29 (1.16, 1.45)
**Obesity**										
No	1.69 (1.56, 1.84)	1.81 (1.70, 1.94)	1.37 (1.30, 1.44)	1.34 (1.25, 1.44)	1.42 (1.31, 1.54)	1.49 (1.43, 1.57)	1.52 (1.47, 1.57)	1.28 (1.23, 1.33)	1.48 (1.40, 1.58)	1.50 (1.43, 1.58)
Yes	1.59 (1.47, 1.71)	1.68 (1.57, 1.80)	1.28 (1.22, 1.34)	1.24 (1.17, 1.32)	1.40 (1.29, 1.53)	1.33 (1.27, 1.39)	1.32 (1.28, 1.36)	1.21 (1.16, 1.25)	1.39 (1.31, 1.48)	1.34 (1.29, 1.40)
**Smoking**										
No/former	1.48 (1.40, 1.56)	1.64 (1.58, 1.71)	1.27 (1.23, 1.31)	1.17 (1.14, 1.21)	1.30 (1.23, 1.36)	1.20 (1.18, 1.22)	1.36 (1.33, 1.38)	1.07 (1.04, 1.09)	1.44 (1.37, 1.51)	1.25 (1.23, 1.26)
Yes	1.57 (1.38, 1.80)	1.93 (1.72, 2.17)	1.32 (1.22, 1.44)	1.31 (1.18, 1.46)	1.40 (1.23, 1.59)	1.47 (1.37, 1.59)	1.39 (1.31, 1.47)	1.37 (1.28, 1.46)	1.38 (1.24, 1.54)	1.52 (1.40, 1.65)

Risk Factors	COVID-19 vs Contemporary Control – Hazard Ratio (95%CI)*									
	**Cerebro-vascular disorders**	**Cognition and memory disorders**	**Disorders of peripheral nerves**	**Episodic disorders**	**Extra-pyramidal and movement disorders**	**Mental health disorders**	**Musculo-skeletal disorders**	**Sensory disorders**	**Other neurologic or related disorders**	**Any neurologic outcome**
**ADI**										
≤Median	1.67 (1.53, 1.83)	1.78 (1.66, 1.90)	1.31 (1.24, 1.38)	1.30 (1.22, 1.39)	1.45 (1.34, 1.58)	1.42 (1.36, 1.49)	1.43 (1.38, 1.48)	1.19 (1.14, 1.24)	1.44 (1.35, 1.53)	1.39 (1.32, 1.46)
>Median	1.63 (1.50, 1.77)	1.70 (1.59, 1.83)	1.32 (1.25, 1.39)	1.31 (1.21, 1.41)	1.41 (1.29, 1.54)	1.39 (1.33, 1.46)	1.41 (1.37, 1.46)	1.28 (1.23, 1.33)	1.45 (1.36, 1.55)	1.45 (1.39, 1.52)
**Diabetes**										
No	1.74 (1.60, 1.89)	1.85 (1.73, 1.97)	1.34 (1.27, 1.41)	1.28 (1.20, 1.36)	1.46 (1.36, 1.57)	1.40 (1.35, 1.46)	1.44 (1.40, 1.48)	1.26 (1.21, 1.30)	1.47 (1.40, 1.56)	1.43 (1.37, 1.48)
Yes	1.43 (1.32, 1.54)	1.50 (1.40, 1.61)	1.28 (1.21, 1.35)	1.40 (1.28, 1.53)	1.30 (1.19, 1.41)	1.42 (1.35, 1.50)	1.38 (1.32, 1.43)	1.22 (1.17, 1.28)	1.35 (1.26, 1.45)	1.35 (1.28, 1.43)
**Chronic kidney disease**										
No	1.65 (1.54, 1.77)	1.76 (1.66, 1.86)	1.33 (1.27, 1.39)	1.29 (1.22, 1.37)	1.43 (1.34, 1.53)	1.39 (1.34, 1.45)	1.42 (1.38, 1.46)	1.26 (1.22, 1.30)	1.46 (1.39, 1.53)	1.41 (1.36, 1.47)
Yes	1.49 (1.35, 1.63)	1.65 (1.52, 1.79)	1.25 (1.16, 1.35)	1.48 (1.33, 1.66)	1.35 (1.20, 1.52)	1.49 (1.39, 1.60)	1.47 (1.40, 1.55)	1.20 (1.14, 1.27)	1.39 (1.25, 1.54)	1.43 (1.34, 1.54)
**Hyperlipidemia**										
No	2.01 (1.78, 2.27)	2.22 (2.02, 2.44)	1.40 (1.31, 1.49)	1.27 (1.18, 1.37)	1.65 (1.48, 1.83)	1.51 (1.43, 1.59)	1.55 (1.48, 1.61)	1.20 (1.14, 1.26)	1.51 (1.40, 1.64)	1.47 (1.40, 1.55)
Yes	1.44 (1.35, 1.53)	1.49 (1.41, 1.57)	1.24 (1.19, 1.30)	1.29 (1.21, 1.38)	1.27 (1.19, 1.35)	1.32 (1.27, 1.38)	1.32 (1.28, 1.36)	1.25 (1.21, 1.29)	1.35 (1.29, 1.42)	1.35 (1.30, 1.41)
**Hypertension**										
No	1.68 (1.55, 1.81)	1.74 (1.65, 1.85)	1.32 (1.26, 1.38)	1.28 (1.21, 1.36)	1.42 (1.33, 1.52)	1.38 (1.33, 1.43)	1.43 (1.39, 1.47)	1.24 (1.20, 1.28)	1.42 (1.35, 1.49)	1.42 (1.36, 1.47)
Yes	1.53 (1.39, 1.68)	1.77 (1.61, 1.94)	1.32 (1.24, 1.41)	1.34 (1.21, 1.48)	1.42 (1.23, 1.63)	1.47 (1.38, 1.56)	1.40 (1.34, 1.47)	1.25 (1.19, 1.32)	1.49 (1.36, 1.64)	1.39 (1.31, 1.47)
**Immune dysfunction**										
No	1.64 (1.54, 1.76)	1.80 (1.71, 1.90)	1.32 (1.26, 1.37)	1.29 (1.22, 1.37)	1.42 (1.33, 1.51)	1.39 (1.34, 1.44)	1.43 (1.40, 1.47)	1.25 (1.21, 1.29)	1.45 (1.39, 1.52)	1.42 (1.38, 1.47)
Yes	1.40 (1.23, 1.58)	1.44 (1.29, 1.60)	1.33 (1.22, 1.45)	1.42 (1.25, 1.62)	1.30 (1.13, 1.50)	1.45 (1.33, 1.59)	1.36 (1.28, 1.45)	1.19 (1.11, 1.28)	1.27 (1.14, 1.41)	1.28 (1.16, 1.41)

*. Adjustment through inverse probability weighting using predefined and algorithmically selected high-dimensional variables.
†. Obesity was defined based on baseline BMI>30kg/m2
‡. Chronic kidney disease was defined based on baseline outpatient estimated Glomerular Filtration Rate< 60 mL/min/1.73 m2
CI, confidence interval; ADI, area deprivation index.

Supplementary Table 5. Demographic and health characteristics of the COVID-19 and contemporary cohorts by care setting of the acute infection before weighting

Baseline Characteristics	Non-hospitalized (N=131,915)	Hospitalized (N=16,764)	Admitted to intensive care (N=5,389)	Contemporary control (N=5,606,761)	Absolute standardized difference		
					Non-hospitalized and Contemporary control	Hospitalized and Contemporary control	Admitted to intensive care and Contemporary control
**Age, mean (std), yr**	60.23 (15.72)	68.38 (13.38)	68.94 (12.03)	63.44 (16.24)	0.20	0.32	0.39
**Race, no. (%)**							
White	95,280 (72.23)	10,587 (63.15)	3,359 (62.33)	4,306,991 (76.82)	0.11	0.30	0.32
Black	30,211 (22.90)	5,229 (31.19)	1,719 (31.90)	1,034,479 (18.45)	0.11	0.30	0.31
Other	6,424 (4.87)	948 (5.66)	311 (5.77)	265,291 (4.73)	0.01	0.04	0.05
**Sex, no. (%)**							
Male	116,294 (88.16)	15,778 (94.12)	5,107 (94.77)	5,062,488 (90.29)	0.07	0.14	0.17
Female	15,621 (11.84)	986 (5.88)	282 (5.23)	544,273 (9.71)	0.07	0.14	0.17
**BMI category, no. (%), kg/m^2**							
Underweight/ Normal	17,442 (13.22)	3,132 (18.68)	882 (16.37)	1,054,225 (18.80)	0.15	0.00	0.06
Overweight	42,823 (32.46)	5,234 (31.22)	1,645 (30.53)	2,180,243 (38.89)	0.13	0.16	0.18
Obese	71,650 (54.32)	8,398 (50.10)	2,862 (53.11)	2,372,293 (42.31)	0.24	0.16	0.22
**Smoking status, no. (%)**							
Never	59,907 (45.41)	7,123 (42.49)	2,178 (40.42)	2,388,382 (42.60)	0.06	0.00	0.04
Former	52,008 (39.43)	6,849 (40.86)	2,372 (44.02)	2,020,489 (36.04)	0.07	0.10	0.16
Current	20,000 (15.16)	2,792 (16.66)	839 (15.57)	1,197,890 (21.37)	0.16	0.12	0.15
**Area Deprivation Index[a], mean (std)**	55.65 (18.54)	53.18 (18.70)	54.70 (18.60)	54.68 (19.03)	0.05	0.08	0.00
Clinical Characteristics							
**Outpatient encounter*, no. (%)**							
Zero	13,634 (10.34)	934 (5.57)	274 (5.08)	1,170,562 (20.88)	0.29	0.46	0.48
One	21,616 (16.39)	1,186 (7.08)	369 (6.85)	1,510,754 (26.95)	0.26	0.55	0.56
Two or more	96,665 (73.28)	14,644 (87.35)	4,746 (88.07)	2,925,445 (52.18)	0.45	0.83	0.85

Baseline Characteristics	Non-hospitalized (N=131,915)	Hospitalized (N=16,764)	Admitted to intensive care (N=5,389)	Contemporary control (N=5,606,761)	Absolute standardized difference		
					Non-hospitalized and Contemporary control	Hospitalized and Contemporary control	Admitted to intensive care and Contemporary control
**Long-term care, no. (%)**	3,891 (2.95)	1,727 (10.30)	497 (9.22)	30,972 (0.55)	0.18	0.44	0.41
**eGFR, mean (std), ml/min/1.73m^2**	79.47 (21.31)	68.27 (26.12)	65.07 (26.78)	78.63 (20.10)	0.04	0.44	0.57
**Systolic blood pressure, mean (std), mmHg**	132.35 (11.69)	134.38 (11.83)	134.56 (12.14)	132.64 (12.29)	0.02	0.14	0.16
**Diastolic blood pressure, mean (std), mmHg**	78.54 (7.37)	77.06 (7.46)	76.99 (7.73)	77.78 (7.54)	0.10	0.10	0.10
**Cancer, no. (%)**	8,988 (6.81)	2,336 (13.94)	776 (14.40)	318,651 (5.68)	0.05	0.28	0.29
**Chronic kidney disease, no. (%)**	21,971 (16.66)	5,946 (35.47)	2,137 (39.66)	930,384 (16.59)	0.00	0.44	0.53
**Chronic lung disease, no. (%)**	17,053 (12.93)	4,077 (24.32)	1,484 (27.54)	564,387 (10.07)	0.09	0.38	0.46
**Diabetes mellitus type 2, no. (%)**	37,476 (28.41)	7,468 (44.55)	2,622 (48.66)	1,188,545 (21.20)	0.17	0.51	0.60
**Hyperlipidemia, no. (%)**	75,207 (57.01)	11,722 (69.92)	3,945 (73.21)	2,557,756 (45.62)	0.23	0.51	0.59
**Hypertension, no. (%)**	34,140 (25.88)	5,292 (31.57)	1,710 (31.73)	1,474,746 (26.30)	0.01	0.12	0.12

[a]Area Deprivation Index is a measure of socioeconomic disadvantage, with a range from low to high disadvantage of 0 to 100.
*.Data collected within one year before cohort enrollment
std, standard deviation; BMI, body mass index; eGFR, estimated glomerular filtration rate.

Supplementary Table 6. Demographic and health characteristics of the COVID-19 and contemporary cohorts by care setting of the acute infection after weighting

Baseline Characteristics	Non-hospitalized (N=131,915)	Hospitalized (N=16,764)	Admitted to intensive care (N=5,389)	Contemporary control (N=5,606,761)	Absolute standardized difference		
					Non-hospitalized and Contemporary control	Hospitalized and Contemporary control	Admitted to intensive care and Contemporary control
**Age, mean (std), yr**	62.58 (16.31)	63.56 (15.40)	62.80 (16.14)	63.14 (16.28)	0.03	0.03	0.02
**Race, no. (%)**							
White	100,530 (76.21)	12,258 (73.12)	3,982 (73.89)	4,313,786 (76.94)	0.02	0.09	0.07
Black	25,010 (18.96)	3,644 (21.74)	1,063 (19.72)	1,026,990 (18.32)	0.02	0.09	0.04
Other	6,375 (4.83)	862 (5.14)	345 (6.40)	265,985 (4.74)	0.00	0.02	0.07
**Sex, no. (%)**							
Male	118,793 (90.05)	15,354 (91.59)	4,954 (91.94)	5,066,942 (90.37)	0.01	0.04	0.06
Female	13,122 (9.95)	1,410 (8.41)	435 (8.06)	539,819 (9.63)	0.01	0.04	0.06
**BMI category, no. (%), kg/m²**							
Underweight/ Normal	24,825 (18.82)	3,099 (18.49)	929 (17.23)	1,067,527 (19.04)	0.01	0.01	0.05
Overweight	48,640 (36.87)	5,767 (34.40)	1,899 (35.24)	2,169,929 (38.70)	0.04	0.09	0.07
Obese	58,450 (44.31)	7,898 (47.11)	2,561 (47.53)	2,369,305 (42.26)	0.04	0.10	0.11
**Smoking status, no. (%)**							
Never	60,136 (45.59)	7,609 (45.39)	2,487 (46.14)	2,681,770 (47.83)	0.04	0.05	0.03
Former	41,442 (31.42)	5,409 (32.27)	1,655 (30.70)	1,695,036 (30.23)	0.03	0.04	0.01
Current	30,335 (23.00)	3,746 (22.34)	1,248 (23.15)	1,230,011 (21.94)	0.03	0.01	0.03
**Area Deprivation Index[a], mean (std)**	54.86 (19.00)	54.45 (19.17)	55.73 (19.27)	54.66 (19.08)	0.01	0.01	0.06
Clinical Characteristics							
**Outpatient encounter*, no. (%)**							
Zero	20,688 (15.68)	2,528 (15.08)	787 (14.60)	892,036 (15.91)	0.01	0.02	0.04
One	35,930 (27.24)	4,056 (24.19)	1,582 (29.35)	1,571,295 (28.03)	0.02	0.09	0.03
Two or more	75,296 (57.08)	10,180 (60.73)	3,021 (56.05)	3,143,431 (56.07)	0.02	0.09	0.00

Baseline Characteristics	Non-hospitalized (N=131,915)	Hospitalized (N=16,764)	Admitted to intensive care (N=5,389)	Contemporary control (N=5,606,761)	Absolute standardized difference		
					Non-hospitalized and Contemporary control	Hospitalized and Contemporary control	Admitted to intensive care and Contemporary control
**Long-term care, no. (%)**	1,320 (1.00)	303 (1.81)	102 (1.89)	44,798 (0.80)	0.02	0.09	0.09
**eGFR, mean (std), ml/min/1.73m^2**	79.10 (20.43)	77.82 (21.57)	78.02 (21.44)	78.94 (20.05)	0.01	0.05	0.04
**Systolic blood pressure, mean (std), mmHg**	132.55 (12.48)	133.45 (12.50)	133.31 (12.30)	132.63 (12.41)	0.01	0.07	0.06
**Diastolic blood pressure, mean (std), mmHg**	77.71 (7.73)	78.11 (7.67)	78 (7.68)	77.66 (7.70)	0.01	0.06	0.04
**Cancer, no. (%)**	8,020 (6.08)	1,196 (7.14)	360 (6.68)	341,564 (6.09)	0.00	0.04	0.02
**Chronic kidney disease, no. (%)**	21,634 (16.40)	3,189 (19.02)	998 (18.52)	901,063 (16.07)	0.01	0.08	0.06
**Chronic lung disease, no. (%)**	14,520 (11.01)	2,069 (12.34)	628 (11.65)	600,596 (10.71)	0.01	0.05	0.03
**Diabetes mellitus type 2, no. (%)**	29,764 (22.56)	4,391 (26.19)	1,418 (26.32)	1,240,832 (22.13)	0.01	0.09	0.10
**Hyperlipidemia, no. (%)**	66,091 (50.10)	8,656 (51.64)	2,782 (51.62)	2,728,306 (48.66)	0.03	0.06	0.06
**Hypertension, no. (%)**	35,320 (26.78)	4,942 (29.48)	1,561 (28.97)	1,470,373 (26.23)	0.01	0.07	0.06

[a]Area Deprivation Index is a measure of socioeconomic disadvantage, with a range from low to high disadvantage of 0 to 100.
*.Data collected within one year before cohort enrollment
std, standard deviation; BMI, body mass index; eGFR, estimated glomerular filtration rate.

Supplementary Table 7. Risks and 12-month burdens of post-acute COVID-19 neurologic outcomes by care setting of the acute infection compared to contemporary control

Outcome*	Care setting†	Hazard ratio (95% CI)‡ COVID-19 vs Contemporary control	COVID-19 burden per 1000 persons at 12 months (95% CI)‡	Contemporary control burden per 1000 persons at 12 months (95% CI)‡	Burden difference per 1000 persons at 12 months (95% CI)‡
**Cerebrovascular**	Non-hospitalized	1.38 (1.30, 1.46)	12.37 (11.68, 13.10)	9.00 (8.93, 9.08)	3.37 (2.68, 4.10)
	Hospitalized	3.02 (2.66, 3.42)	26.92 (23.79, 30.46)		17.92 (14.79, 21.45)
	Admitted to intensive care	4.30 (3.47, 5.32)	38.13 (30.93, 46.96)		29.13 (21.93, 37.96)
Ischemic stroke	Non-hospitalized	1.27 (1.19, 1.36)	8.54 (7.98, 9.14)	6.71 (6.65, 6.78)	1.83 (1.27, 2.42)
	Hospitalized	3.18 (2.76, 3.66)	21.18 (18.43, 24.34)		14.47 (11.72, 17.63)
	Admitted to intensive care	4.30 (3.38, 5.48)	28.55 (22.48, 36.24)		21.84 (15.77, 29.53)
TIA	Non-hospitalized	1.50 (1.37, 1.64)	5.01 (4.59, 5.48)	3.35 (3.30, 3.39)	1.67 (1.24, 2.14)
	Hospitalized	2.51 (2.05, 3.08)	8.38 (6.84, 10.28)		5.04 (3.49, 6.93)
	Admitted to intensive care	3.18 (2.23, 4.54)	10.62 (7.45, 15.12)		7.27 (4.10, 11.77)
Hemorrhagic stroke	Non-hospitalized	1.56 (1.07, 2.29)	0.27 (0.19, 0.40)	0.17 (0.16, 0.18)	0.10 (0.01, 0.22)
	Hospitalized	4.21 (2.40, 7.38)	0.73 (0.42, 1.28)		0.56 (0.24, 1.11)
	Admitted to intensive care	18.34 (7.47, 45.01)	3.18 (1.30, 7.79)		3.01 (1.12, 7.62)
Cerebral venous thrombosis	Non-hospitalized	0.84 (0.45, 1.58)	0.05 (0.03, 0.09)	0.06 (0.05, 0.06)	-0.01 (-0.03, 0.03)
	Hospitalized	8.09 (3.44, 19.02)	0.47 (0.20, 1.10)		0.41 (0.14, 1.05)
	Admitted to intensive care	24.00 (8.92, 64.56)	1.39 (0.52, 3.74)		1.33 (0.46, 3.68)
**Cognition and memory**	Non-hospitalized	1.40 (1.33, 1.47)	18.36 (17.51, 19.24)	13.16 (13.07, 13.26)	5.19 (4.35, 6.08)
	Hospitalized	3.73 (3.38, 4.12)	48.21 (43.75, 53.11)		35.04 (30.58, 39.94)
	Admitted to intensive care	4.98 (4.15, 5.97)	63.81 (53.52, 76.00)		50.65 (40.36, 62.83)
Memory problems	Non-hospitalized	1.40 (1.34, 1.47)	18.18 (17.34, 19.06)	13.00 (12.90, 13.09)	5.18 (4.34, 6.06)
	Hospitalized	3.71 (3.36, 4.10)	47.39 (42.99, 52.23)		34.39 (29.99, 39.23)
	Admitted to intensive care	4.84 (4.04, 5.80)	61.34 (51.47, 73.03)		48.35 (38.47, 60.04)
Alzheimer’s disease	Non-hospitalized	1.47 (1.28, 1.69)	2.33 (2.03, 2.68)	1.58 (1.55, 1.61)	0.74 (0.44, 1.09)
	Hospitalized	3.90 (3.07, 4.97)	6.17 (4.85, 7.84)		4.58 (3.27, 6.26)
	Admitted to intensive care	5.18 (3.27, 8.22)	8.18 (5.17, 12.94)		6.60 (3.58, 11.36)

Outcome*	Care setting†	Hazard ratio (95% CI)‡ COVID-19 vs Contemporary control	COVID-19 burden per 1000 persons at 12 months (95% CI)‡	Contemporary control burden per 1000 persons at 12 months (95% CI)‡	Burden difference per 1000 persons at 12 months (95% CI)‡
**Disorders of peripheral nerves**	Non-hospitalized	1.29 (1.25, 1.34)	33.28 (32.23, 34.36)	25.81 (25.68, 25.94)	7.47 (6.43, 8.56)
	Hospitalized	2.10 (1.91, 2.31)	53.41 (48.66, 58.61)		27.60 (22.85, 32.80)
	Admitted to intensive care	2.25 (1.89, 2.68)	57.07 (48.11, 67.64)		31.27 (22.31, 41.84)
Peripheral neuropathy	Non-hospitalized	1.25 (1.20, 1.30)	20.82 (20.01, 21.66)	16.74 (16.63, 16.84)	4.08 (3.27, 4.92)
	Hospitalized	2.00 (1.79, 2.25)	33.26 (29.69, 37.25)		16.52 (12.95, 20.51)
	Admitted to intensive care	2.15 (1.75, 2.65)	35.72 (29.13, 43.76)		18.98 (12.39, 27.03)
Paresthesia	Non-hospitalized	1.37 (1.30, 1.44)	12.35 (11.75, 12.99)	9.06 (8.99, 9.14)	3.29 (2.69, 3.92)
	Hospitalized	1.92 (1.63, 2.26)	17.30 (14.70, 20.35)		8.24 (5.64, 11.29)
	Admitted to intensive care	2.02 (1.54, 2.66)	18.24 (13.92, 23.89)		9.18 (4.86, 14.83)
Dysautonomia	Non-hospitalized	1.23 (1.14, 1.32)	6.55 (6.10, 7.04)	5.35 (5.29, 5.41)	1.20 (0.75, 1.69)
	Hospitalized	2.20 (1.83, 2.64)	11.74 (9.79, 14.09)		6.39 (4.44, 8.74)
	Admitted to intensive care	2.31 (1.59, 3.35)	12.30 (8.48, 17.82)		6.95 (3.13, 12.47)
Bell's palsy	Non-hospitalized	1.53 (1.28, 1.83)	1.00 (0.84, 1.20)	0.65 (0.63, 0.67)	0.35 (0.18, 0.54)
	Hospitalized	2.90 (1.87, 4.49)	1.89 (1.22, 2.93)		1.24 (0.57, 2.28)
	Admitted to intensive care	3.14 (1.76, 5.60)	2.05 (1.15, 3.66)		1.40 (0.50, 3.01)
**Episodic disorders**	Non-hospitalized	1.32 (1.26, 1.37)	19.33 (18.55, 20.14)	14.69 (14.59, 14.79)	4.64 (3.85, 5.45)
	Hospitalized	2.00 (1.75, 2.29)	29.16 (25.54, 33.29)		14.47 (10.84, 18.60)
	Admitted to intensive care	2.57 (1.99, 3.32)	37.37 (29.05, 48.01)		22.68 (14.36, 33.32)
Migraine	Non-hospitalized	1.25 (1.19, 1.31)	12.36 (11.77, 12.98)	9.92 (9.84, 10.00)	2.44 (1.85, 3.07)
	Hospitalized	1.65 (1.36, 1.99)	16.28 (13.48, 19.66)		6.37 (3.56, 9.74)
	Admitted to intensive care	1.53 (1.04, 2.27)	15.15 (10.27, 22.32)		5.23 (0.35, 12.4)
Epilepsy and seizures	Non-hospitalized	1.41 (1.28, 1.55)	4.03 (3.65, 4.44)	2.86 (2.82, 2.90)	1.17 (0.79, 1.58)
	Hospitalized	3.27 (2.66, 4.02)	9.33 (7.60, 11.45)		6.47 (4.74, 8.59)
	Admitted to intensive care	5.32 (3.89, 7.28)	15.12 (11.08, 20.62)		12.26 (8.22, 17.76)
Headache disorders	Non-hospitalized	1.41 (1.32, 1.51)	5.90 (5.51, 6.31)	4.19 (4.14, 4.24)	1.71 (1.32, 2.12)
	Hospitalized	1.75 (1.40, 2.18)	7.31 (5.87, 9.10)		3.12 (1.68, 4.91)
	Admitted to intensive care	2.91 (1.79, 4.74)	12.15 (7.49, 19.70)		7.96 (3.29, 15.51)
**Extrapyramidal and movement disorders**	Non-hospitalized	1.30 (1.23, 1.37)	12.46 (11.81, 13.15)	9.59 (9.51, 9.67)	2.87 (2.22, 3.56)
	Hospitalized	2.32 (2.03, 2.66)	22.15 (19.37, 25.32)		12.56 (9.78, 15.73)
	Admitted to intensive care	2.05 (1.58, 2.66)	19.52 (15.07, 25.26)		9.93 (5.49, 15.67)

Outcome*	Care setting†	Hazard ratio (95% CI)‡ COVID-19 vs Contemporary control	COVID-19 burden per 1000 persons at 12 months (95% CI)‡	Contemporary control burden per 1000 persons at 12 months (95% CI)‡	Burden difference per 1000 persons at 12 months (95% CI)‡
Abnormal involuntary movements	Non-hospitalized	1.34 (1.26, 1.42)	9.23 (8.68, 9.81)	6.90 (6.84, 6.97)	2.32 (1.77, 2.91)
	Hospitalized	2.24 (1.92, 2.60)	15.37 (13.21, 17.88)		8.47 (6.31, 10.98)
	Admitted to intensive care	1.86 (1.37, 2.54)	12.83 (9.43, 17.46)		5.93 (2.53, 10.55)
Tremor	Non-hospitalized	1.27 (1.15, 1.40)	3.76 (3.41, 4.14)	2.96 (2.92, 3.01)	0.79 (0.44, 1.17)
	Hospitalized	2.02 (1.57, 2.60)	5.98 (4.65, 7.68)		3.01 (1.68, 4.72)
	Admitted to intensive care	1.70 (1.13, 2.57)	5.04 (3.33, 7.61)		2.07 (0.37, 4.64)
Parkinson-like disease	Non-hospitalized	1.12 (0.97, 1.29)	1.99 (1.72, 2.30)	1.78 (1.74, 1.81)	0.21 (-0.05, 0.52)
	Hospitalized	2.34 (1.73, 3.16)	4.16 (3.08, 5.61)		2.38 (1.30, 3.84)
	Admitted to intensive care	1.89 (1.00, 3.58)	3.36 (1.78, 6.35)		1.58 (0.00, 4.57)
Dystonia	Non-hospitalized	1.38 (1.15, 1.65)	0.96 (0.80, 1.15)	0.69 (0.67, 0.71)	0.26 (0.10, 0.45)
	Hospitalized	2.25 (1.43, 3.55)	1.56 (0.99, 2.46)		0.87 (0.30, 1.76)
	Admitted to intensive care	2.14 (1.08, 4.23)	1.48 (0.75, 2.93)		0.79 (0.06, 2.24)
Myoclonus	Non-hospitalized	1.11 (0.80, 1.53)	0.35 (0.25, 0.49)	0.32 (0.30, 0.33)	0.03 (-0.06, 0.17)
	Hospitalized	2.72 (1.69, 4.35)	0.86 (0.54, 1.38)		0.55 (0.22, 1.07)
	Admitted to intensive care	4.09 (2.05, 8.15)	1.30 (0.65, 2.59)		0.98 (0.34, 2.27)
**Mental health disorders**	Non-hospitalized	1.35 (1.31, 1.39)	81.40 (79.18, 83.68)	60.90 (60.66, 61.14)	20.50 (18.27, 22.78)
	Hospitalized	2.40 (2.20, 2.62)	140.16 (129.30, 151.85)		79.25 (68.39, 90.95)
	Admitted to intensive care	2.95 (2.47, 3.53)	169.34 (143.91, 198.71)		108.44 (83.01, 137.81)
Major depressive disorders	Non-hospitalized	1.32 (1.28, 1.36)	53.34 (51.81, 54.91)	40.67 (40.49, 40.85)	12.67 (11.14, 14.24)
	Hospitalized	2.42 (2.22, 2.65)	95.69 (87.91, 104.12)		55.03 (47.25, 63.46)
	Admitted to intensive care	2.62 (2.17, 3.17)	103.20 (86.10, 123.45)		62.53 (45.43, 82.79)
Stress and adjustment disorders	Non-hospitalized	1.36 (1.32, 1.40)	50.51 (49.01, 52.05)	37.41 (37.24, 37.59)	13.09 (11.60, 14.64)
	Hospitalized	2.20 (2.00, 2.42)	80.46 (73.41, 88.14)		43.04 (36.00, 50.73)
	Admitted to intensive care	2.21 (1.80, 2.72)	80.91 (66.26, 98.63)		43.50 (28.85, 61.21)
Anxiety disorders	Non-hospitalized	1.32 (1.28, 1.36)	43.93 (42.65, 45.26)	33.57 (33.41, 33.72)	10.37 (9.08, 11.69)
	Hospitalized	2.33 (2.12, 2.55)	76.42 (69.95, 83.46)		42.85 (36.39, 49.89)
	Admitted to intensive care	2.52 (2.10, 3.03)	82.48 (69.06, 98.35)		48.91 (35.50, 64.79)
Psychotic disorders	Non-hospitalized	1.20 (1.06, 1.36)	2.35 (2.07, 2.67)	1.96 (1.93, 2.00)	0.39 (0.11, 0.71)
	Hospitalized	4.48 (3.46, 5.79)	8.75 (6.77, 11.30)		6.79 (4.81, 9.34)
	Admitted to intensive care	3.91 (2.18, 7.00)	7.64 (4.27, 13.66)		5.68 (2.31, 11.70)

Outcome*	Care setting†	Hazard ratio (95% CI)‡ COVID-19 vs Contemporary control	COVID-19 burden per 1000 persons at 12 months (95% CI)‡	Contemporary control burden per 1000 persons at 12 months (95% CI)‡	Burden difference per 1000 persons at 12 months (95% CI)‡
**Musculoskeletal**	Non-hospitalized	1.43 (1.40, 1.46)	134.06 (131.44, 136.72)	95.77 (95.49, 96.05)	38.29 (35.67, 40.95)
	Hospitalized	2.32 (2.17, 2.48)	208.02 (196.09, 220.58)		112.25 (100.32, 124.81)
	Admitted to intensive care	2.76 (2.41, 3.17)	242.77 (215.43, 272.95)		147.00 (119.66, 177.18)
Joint pain	Non-hospitalized	1.39 (1.36, 1.42)	116.65 (114.26, 119.09)	85.58 (85.31, 85.84)	31.07 (28.68, 33.51)
	Hospitalized	1.73 (1.60, 1.86)	143.01 (133.27, 153.39)		57.43 (47.70, 67.81)
	Admitted to intensive care	1.69 (1.44, 1.99)	140.50 (120.53, 163.45)		54.92 (34.95, 77.87)
Myalgia	Non-hospitalized	1.49 (1.43, 1.54)	28.38 (27.42, 29.38)	19.20 (19.09, 19.31)	9.18 (8.22, 10.18)
	Hospitalized	4.38 (4.07, 4.71)	81.32 (75.79, 87.23)		62.12 (56.59, 68.03)
	Admitted to intensive care	6.30 (5.48, 7.24)	114.89 (100.71, 130.92)		95.69 (81.51, 111.73)
Myopathy	Non-hospitalized	1.57 (1.26, 1.96)	0.62 (0.50, 0.77)	0.39 (0.38, 0.41)	0.22 (0.10, 0.38)
	Hospitalized	6.65 (4.75, 9.32)	2.61 (1.86, 3.65)		2.22 (1.47, 3.26)
	Admitted to intensive care	36.72 (26.08, 51.68)	14.32 (10.20, 20.10)		13.93 (9.80, 19.71)
**Sensory disorders**	Non-hospitalized	1.23 (1.20, 1.26)	86.64 (84.68, 88.66)	70.91 (70.67, 71.15)	15.73 (13.76, 17.74)
	Hospitalized	1.43 (1.32, 1.54)	99.76 (92.72, 107.31)		28.85 (21.80, 36.40)
	Admitted to intensive care	1.50 (1.27, 1.77)	104.43 (89.19, 122.10)		33.52 (18.28, 51.19)
Hearing abnormalities and tinnitus	Non-hospitalized	1.21 (1.18, 1.24)	68.32 (66.61, 70.08)	56.86 (56.65, 57.07)	11.46 (9.75, 13.21)
	Hospitalized	1.27 (1.16, 1.38)	71.43 (65.72, 77.61)		14.56 (8.85, 20.74)
	Admitted to intensive care	1.38 (1.14, 1.66)	77.36 (64.60, 92.52)		20.50 (7.74, 35.66)
Vision abnormalities	Non-hospitalized	1.28 (1.23, 1.33)	24.17 (23.25, 25.12)	18.98 (18.87, 19.10)	5.19 (4.27, 6.14)
	Hospitalized	1.72 (1.54, 1.92)	32.38 (28.99, 36.15)		13.39 (10.01, 17.16)
	Admitted to intensive care	1.77 (1.43, 2.19)	33.32 (27.00, 41.07)		14.33 (8.02, 22.09)
Loss of smell	Non-hospitalized	3.98 (3.43, 4.61)	1.40 (1.20, 1.62)	0.35 (0.34, 0.37)	1.04 (0.85, 1.27)
	Hospitalized	4.44 (2.57, 7.68)	1.56 (0.90, 2.69)		1.21 (0.55, 2.34)
	Admitted to intensive care	7.51 (3.93, 14.35)	2.63 (1.38, 5.02)		2.28 (1.03, 4.67)
Loss of taste	Non-hospitalized	2.06 (1.35, 3.13)	0.18 (0.12, 0.28)	0.09 (0.08, 0.10)	0.10 (0.03, 0.19)
	Hospitalized	4.76 (1.84, 12.36)	0.43 (0.16, 1.11)		0.34 (0.07, 1.02)
	Admitted to intensive care	10.02 (2.53, 39.76)	0.90 (0.23, 3.56)		0.81 (0.14, 3.47)
**Other neurologic or related disorders**	Non-hospitalized	1.40 (1.35, 1.46)	22.64 (21.79, 23.53)	16.18 (16.08, 16.28)	6.46 (5.61, 7.35)
	Hospitalized	2.40 (2.16, 2.66)	38.33 (34.61, 42.44)		22.15 (18.43, 26.26)
	Admitted to intensive care	2.60 (2.10, 3.21)	41.51 (33.70, 51.08)		25.33 (17.51, 34.90)

Outcome*	Care setting†	Hazard ratio (95% CI)‡ COVID-19 vs Contemporary control	COVID-19 burden per 1000 persons at 12 months (95% CI)‡	Contemporary control burden per 1000 persons at 12 months (95% CI)‡	Burden difference per 1000 persons at 12 months (95% CI)‡
Dizziness	Non-hospitalized	1.39 (1.34, 1.45)	21.27 (20.45, 22.13)	15.30 (15.20, 15.40)	5.98 (5.15, 6.83)
	Hospitalized	2.39 (2.15, 2.66)	36.23 (32.61, 40.25)		20.94 (17.32, 24.95)
	Admitted to intensive care	2.55 (2.04, 3.19)	38.52 (30.90, 47.98)		23.23 (15.60, 32.68)
Somnolence	Non-hospitalized	1.37 (1.17, 1.62)	1.13 (0.96, 1.33)	0.82 (0.80, 0.85)	0.31 (0.14, 0.51)
	Hospitalized	1.64 (1.06, 2.52)	1.35 (0.88, 2.08)		0.53 (0.05, 1.25)
	Admitted to intensive care	2.31 (1.34, 3.98)	1.91 (1.11, 3.28)		1.08 (0.28, 2.45)
Guillain-Barre syndrome	Non-hospitalized	1.85 (1.13, 3.03)	0.17 (0.10, 0.28)	0.09 (0.08, 0.10)	0.08 (0.01, 0.19)
	Hospitalized	6.4 (2.29, 17.91)	0.58 (0.21, 1.63)		0.49 (0.12, 1.54)
	Admitted to intensive care	8.5 (3.71, 19.44)	0.77 (0.34, 1.77)		0.68 (0.25, 1.68)
Encephalitis or encephalopathy	Non-hospitalized	1.85 (1.13, 3.04)	0.16 (0.10, 0.26)	0.09 (0.08, 0.09)	0.07 (0.01, 0.17)
	Hospitalized	4.41 (2.20, 8.86)	0.38 (0.19, 0.75)		0.29 (0.10, 0.67)
	Admitted to intensive care	6.13 (2.42, 15.51)	0.52 (0.21, 1.32)		0.44 (0.12, 1.24)
Transverse myelitis	Non-hospitalized	1.34 (0.51, 3.55)	0.04 (0.02, 0.11)	0.03 (0.03, 0.04)	0.01 (-0.02, 0.08)
	Hospitalized	3.37 (1.19, 9.56)	0.11 (0.04, 0.30)		0.07 (0.01, 0.27)
	Admitted to intensive care	4.47 (1.05, 19.03)	0.14 (0.03, 0.60)		0.11 (0.00, 0.57)
**Any neurologic outcome**	Non-hospitalized	1.35 (1.30, 1.39)	252.70 (245.54, 260.04)	194.72 (194.14, 195.30)	57.98 (50.81, 65.31)
	Hospitalized	2.87 (2.57, 3.22)	463.47 (426.75, 501.78)		268.75 (232.03, 307.05)
	Admitted to intensive care	4.00 (3.02, 5.31)	579.94 (480.33, 683.14)		385.21 (285.61, 488.42)

*. Outcomes were ascertained from day 30 after the initial positive COVID-19 test result until end of follow up
†. Based on care received within the first 30 days after a positive COVID-19 test result.
‡. Adjustment through inverse probability weighting using predefined variables and algorithmically selected high-dimensional variables.
CI, confidence interval; TIA, transient ischemic attack.

Supplementary Table 8. Demographic and health characteristics of the COVID-19 and historical cohorts by care setting of the acute infection before weighting

Baseline Characteristics	Non-hospitalized (N=131,915)	Hospitalized (N=16,764)	Admitted to intensive care (N=5,389)	Historical control (N=5,809,908)	Absolute standardized difference		
					Non-hospitalized and Historical control	Hospitalized and Historical control	Admitted to intensive care and Historical control
**Age, mean (std), yr**	60.23 (15.72)	68.38 (13.38)	68.94 (12.03)	62.87 (16.49)	0.04	0.02	0.42
**Race, no. (%)**							
White	95,280 (72.23)	10,587 (63.15)	3,359 (62.33)	4,496,934 (77.40)	0.12	0.32	0.33
Black	30,211 (22.90)	5,229 (31.19)	1,719 (31.90)	1,039,979 (17.90)	0.12	0.31	0.33
Other	6,424 (4.87)	948 (5.66)	311 (5.77)	272,995 (4.70)	0.01	0.04	0.05
**Sex, no. (%)**							
Male	116,294 (88.16)	15,778 (94.12)	5,107 (94.77)	5,262,440 (90.58)	0.08	0.13	0.16
Female	15,621 (11.84)	986 (5.88)	282 (5.23)	547,468 (9.42)	0.08	0.13	0.16
**BMI category, no. (%), kg/m^2**							
Underweight/ Normal	17,442 (13.22)	3,132 (18.68)	882 (16.37)	1,110,846 (19.12)	0.16	0.01	0.07
Overweight	42,823 (32.46)	5,234 (31.22)	1,645 (30.53)	2,315,804 (39.86)	0.15	0.18	0.20
Obese	71,650 (54.32)	8,398 (50.10)	2,862 (53.11)	2,383,258 (41.02)	0.27	0.18	0.24
**Smoking status, no. (%)**							
Never	59,907 (45.41)	7,123 (42.49)	2,178 (40.42)	3,066,880 (52.79)	0.15	0.21	0.25
Former	52,008 (39.43)	6,849 (40.86)	2,372 (44.02)	1,428,183 (24.58)	0.32	0.35	0.42
Current	20,000 (15.16)	2,792 (16.66)	839 (15.57)	1,314,845 (22.63)	0.19	0.15	0.18
**Area Deprivation Index[a], mean (std)**	55.65 (18.54)	53.18 (18.70)	54.7 (18.60)	54.65 (19.11)	0.05	0.08	0.00
Clinical Characteristics							
**Outpatient encounter*, no. (%)**							
Zero	13,634 (10.34)	934 (5.57)	274 (5.08)	677,435 (11.66)	0.04	0.22	0.24
One	21,616 (16.39)	1,186 (7.08)	369 (6.85)	1,764,349 (30.37)	0.33	0.63	0.63
Two or more	96,665 (73.28)	14,644 (87.35)	4,746 (88.07)	3,368,124 (57.97)	0.33	0.70	0.72

Baseline Characteristics	Non-hospitalized (N=131,915)	Hospitalized (N=16,764)	Admitted to intensive care (N=5,389)	Historical control (N=5,809,908)	Absolute standardized difference		
					Non-hospitalized and Historical control	Hospitalized and Historical control	Admitted to intensive care and Historical control
**Long-term care, no. (%)**	3,891 (2.95)	1,727 (10.30)	497 (9.22)	42,621 (0.73)	0.17	0.43	0.40
**eGFR, mean (std), ml/min/1.73m^2**	79.47 (21.31)	68.27 (26.12)	65.07 (26.78)	79.36 (19.88)	0.01	0.48	0.61
**Systolic blood pressure, mean (std), mmHg**	132.35 (11.69)	134.38 (11.83)	134.56 (12.14)	132.63 (12.63)	0.02	0.14	0.16
**Diastolic blood pressure, mean (std), mmHg**	78.54 (7.37)	77.06 (7.46)	76.99 (7.73)	77.54 (7.91)	0.13	0.06	0.07
**Cancer, no. (%)**	8,988 (6.81)	2,336 (13.94)	776 (14.40)	333,568 (5.74)	0.04	0.28	0.29
**Chronic kidney disease, no. (%)**	21,971 (16.66)	5,946 (35.47)	2,137 (39.66)	888,014 (15.28)	0.04	0.48	0.57
**Chronic lung disease, no. (%)**	17,053 (12.93)	4,077 (24.32)	1,484 (27.54)	631,949 (10.88)	0.06	0.36	0.43
**Diabetes mellitus type 2, no. (%)**	37,476 (28.41)	7,468 (44.55)	2,622 (48.66)	1,303,681 (22.44)	0.14	0.48	0.57
**Hyperlipidemia, no. (%)**	75,207 (57.01)	11,722 (69.92)	3,945 (73.21)	2,946,998 (50.72)	0.13	0.40	0.48
**Hypertension, no. (%)**	34,140 (25.88)	5,292 (31.57)	1,710 (31.73)	1,525,419 (26.26)	0.01	0.12	0.12

[a]Area Deprivation Index is a measure of socioeconomic disadvantage, with a range from low to high disadvantage of 0 to 100.
*.Data collected within one year before cohort enrollment
std, standard deviation; BMI, body mass index; eGFR, estimated glomerular filtration rate.

Supplementary Table 9. Demographic and health characteristics of the COVID-19 and historical cohorts by care setting of the acute infection after weighting

Baseline Characteristics	Non-hospitalized (N=131,915)	Hospitalized (N=16,764)	Admitted to intensive care (N=5,389)	Historical control (N=5,809,908)	Absolute standardized difference		
					Non-hospitalized and Historical control	Hospitalized and Historical control	Admitted to intensive care and Historical control
**Age, mean (std), yr**	62.58 (16.31)	63.56 (15.40)	62.80 (16.14)	63.19 (16.38)	0.04	0.02	0.02
**Race, no. (%)**							
White	100,530 (76.21)	12,258 (73.12)	3,982 (73.89)	4,471,305 (76.96)	0.02	0.09	0.07
Black	25,010 (18.96)	3,644 (21.74)	1,063 (19.72)	1,062,923 (18.30)	0.02	0.09	0.04
Other	6,375 (4.83)	862 (5.14)	345 (6.40)	275,680 (4.75)	0.00	0.02	0.07
**Sex, no. (%)**							
Male	118,793 (90.05)	15,354 (91.59)	4,954 (91.94)	5,251,634 (90.39)	0.01	0.04	0.05
Female	13,122 (9.95)	1,410 (8.41)	435 (8.06)	558,274 (9.61)	0.01	0.04	0.05
**BMI category, no. (%), kg/m^2**							
Underweight/ Normal	24,825 (18.82)	3,099 (18.49)	929 (17.23)	1,095,458 (18.86)	0.00	0.01	0.04
Overweight	48,640 (36.87)	5,767 (34.40)	1,899 (35.24)	2,311,007 (39.78)	0.06	0.11	0.09
Obese	58,450 (44.31)	7,898 (47.11)	2,561 (47.53)	2,403,385 (41.37)	0.06	0.12	0.12
**Smoking status, no. (%)**							
Never	60,136 (45.59)	7,609 (45.39)	2,487 (46.14)	2,777,949 (47.81)	0.04	0.05	0.03
Former	41,442 (31.42)	5,409 (32.27)	1,655 (30.70)	1,756,568 (30.23)	0.03	0.04	0.01
Current	30,335 (23.00)	3,746 (22.34)	1,248 (23.15)	1,275,391 (21.95)	0.03	0.01	0.03
**Area Deprivation Index[a], mean (std)**	54.86 (19.00)	54.45 (19.17)	55.73 (19.27)	54.67 (19.07)	0.01	0.01	0.06
Clinical Characteristics							
**Outpatient encounter*, no. (%)**							
Zero	20,688 (15.68)	2,528 (15.08)	787 (14.60)	897,166 (15.44)	0.01	0.01	0.02
One	35,930 (27.24)	4,056 (24.19)	1,582 (29.35)	1,664,248 (28.65)	0.03	0.10	0.02
Two or more	75,296 (57.08)	10,180 (60.73)	3,021 (56.05)	3,248,494 (55.91)	0.02	0.10	0.00

Baseline Characteristics	Non-hospitalized (N=131,915)	Hospitalized (N=16,764)	Admitted to intensive care (N=5,389)	Historical control (N=5,809,908)	Absolute standardized difference		
					Non-hospitalized and Historical control	Hospitalized and Historical control	Admitted to intensive care and Historical control
**Long-term care, no. (%)**	1,320 (1.00)	303 (1.81)	102 (1.89)	45,492 (0.78)	0.02	0.09	0.10
**eGFR, mean (std), ml/min/1.73m^2**	79.10 (20.43)	77.82 (21.57)	78.02 (21.44)	78.90 (20.17)	0.01	0.05	0.04
**Systolic blood pressure, mean (std), mmHg**	132.55 (12.48)	133.45 (12.5)	133.31 (12.30)	132.63 (12.46)	0.01	0.07	0.06
**Diastolic blood pressure, mean (std), mmHg**	77.71 (7.73)	78.11 (7.67)	78.00 (7.68)	77.65 (7.74)	0.01	0.06	0.05
**Cancer, no. (%)**	8,020 (6.08)	1,196 (7.14)	360 (6.68)	325,994 (5.61)	0.02	0.06	0.04
**Chronic kidney disease, no. (%)**	21,634 (16.40)	3,189 (19.02)	998 (18.52)	938,068 (16.15)	0.01	0.08	0.06
**Chronic lung disease, no. (%)**	14,520 (11.01)	2,069 (12.34)	628 (11.65)	623,810 (10.74)	0.01	0.05	0.03
**Diabetes mellitus type 2, no. (%)**	29,764 (22.56)	4,391 (26.19)	1,418 (26.32)	1,291,136 (22.22)	0.01	0.09	0.10
**Hyperlipidemia, no. (%)**	66,091 (50.10)	8,656 (51.64)	2,782 (51.62)	2,838,547 (48.86)	0.02	0.06	0.06
**Hypertension, no. (%)**	35,320 (26.78)	4,942 (29.48)	1,561 (28.97)	1,524,636 (26.24)	0.01	0.07	0.06

[a]Area Deprivation Index is a measure of socioeconomic disadvantage, with a range from low to high disadvantage of 0 to 100.
*.Data collected within one year before cohort enrollment
std, standard deviation; BMI, body mass index; eGFR, estimated glomerular filtration rate.

Supplementary Table 10. Risks and 12-month burdens of post-acute COVID-19 neurologic outcomes compared to historical control

Outcome*	Hazard Ratio (95% CI)† COVID-19 vs Historical control	COVID-19 burden per 1000 persons at 12 months (95% CI) †	Historical control burden per 1000 persons at 12 months (95% CI) †	Absolute burden difference per 1000 persons at 12 months (95% CI)†
**Cerebrovascular disorders**	1.47 (1.40, 1.55)	13.82 (13.15, 14.52)	9.40 (9.35, 9.46)	4.41 (3.75, 5.11)
Ischemic stroke	1.43 (1.34, 1.53)	10.20 (9.55, 10.90)	7.15 (7.09, 7.22)	3.05 (2.39, 3.74)
TIA	1.50 (1.38, 1.62)	5.32 (4.92, 5.74)	3.56 (3.53, 3.59)	1.76 (1.36, 2.18)
Hemorrhagic stroke	1.87 (1.39, 2.52)	0.39 (0.29, 0.53)	0.21 (0.20, 0.22)	0.18 (0.08, 0.32)
Cerebral venous thrombosis	2.22 (1.07, 4.63)	0.08 (0.04, 0.18)	0.04 (0.03, 0.04)	0.05 (0.00, 0.14)
**Cognition and memory disorders**	1.74 (1.66, 1.82)	23.48 (22.40, 24.61)	13.57 (13.47, 13.66)	9.91 (8.84, 11.04)
Memory problems	1.71 (1.63, 1.79)	23.37 (22.29, 24.50)	13.74 (13.65, 13.84)	9.63 (8.55, 10.75)
Alzheimer’s disease	1.73 (1.53, 1.97)	3.26 (2.87, 3.70)	1.88 (1.85, 1.92)	1.38 (0.99, 1.82)
**Disorders of peripheral nerves**	1.31 (1.26, 1.36)	34.58 (33.38, 35.81)	26.49 (26.36, 26.62)	8.09 (6.89, 9.32)
Peripheral neuropathy	1.32 (1.27, 1.38)	22.49 (21.52, 23.50)	17.04 (16.94, 17.15)	5.45 (4.48, 6.46)
Paresthesia	1.38 (1.31, 1.46)	11.98 (11.36, 12.64)	8.69 (8.62, 8.76)	3.29 (2.67, 3.95)
Dysautonomia	1.14 (1.06, 1.22)	6.96 (6.48, 7.48)	6.12 (6.05, 6.18)	0.85 (0.37, 1.37)
Bell’s palsy	1.40 (1.17, 1.67)	0.97 (0.82, 1.16)	0.70 (0.68, 0.72)	0.28 (0.12, 0.47)
**Episodic disorders**	1.40 (1.33, 1.47)	19.56 (18.60, 20.57)	14.05 (13.95, 14.14)	5.52 (4.56, 6.53)
Migraine disorders	1.30 (1.23, 1.38)	12.00 (11.32, 12.72)	9.22 (9.15, 9.30)	2.78 (2.10, 3.50)
Epilepsy and seizures	1.62 (1.45, 1.81)	5.25 (4.70, 5.86)	3.23 (3.19, 3.28)	2.01 (1.47, 2.63)
Headache disorders	1.61 (1.49, 1.73)	5.69 (5.29, 6.12)	3.55 (3.50, 3.60)	2.14 (1.74, 2.57)
**Extrapyramidal and movement disorders**	1.44 (1.36, 1.52)	13.64 (12.90, 14.43)	9.49 (9.41, 9.56)	4.16 (3.41, 4.94)
Abnormal involuntary movements	1.49 (1.40, 1.59)	9.81 (9.21, 10.45)	6.60 (6.54, 6.66)	3.21 (2.61, 3.85)
Tremor	1.41 (1.28, 1.55)	4.08 (3.71, 4.49)	2.90 (2.86, 2.94)	1.18 (0.81, 1.59)
Parkinson-like disease	1.47 (1.26, 1.72)	2.68 (2.29, 3.13)	1.82 (1.79, 1.85)	0.86 (0.47, 1.31)
Dystonia	1.55 (1.28, 1.88)	1.10 (0.91, 1.34)	0.71 (0.69, 0.73)	0.39 (0.20, 0.63)
Myoclonus	1.43 (1.14, 1.81)	0.47 (0.37, 0.59)	0.33 (0.31, 0.34)	0.14 (0.04, 0.26)
**Mental health disorders**	1.33 (1.29, 1.37)	86.25 (83.64, 88.94)	65.57 (65.33, 65.82)	20.68 (18.07, 23.37)
Major depressive disorders	1.28 (1.23, 1.32)	58.18 (56.33, 60.09)	45.89 (45.70, 46.07)	12.29 (10.45, 14.20)
Stress and adjustment disorders	1.31 (1.27, 1.36)	52.02 (50.35, 53.75)	39.88 (39.71, 40.06)	12.14 (10.47, 13.87)

Outcome*	Hazard Ratio (95% CI)† COVID-19 vs Historical control	COVID-19 burden per 1000 persons at 12 months (95% CI)†	Historical control burden per 1000 persons at 12 months (95% CI)†	Absolute burden difference per 1000 persons at 12 months (95% CI)†
Anxiety disorders	1.33 (1.28, 1.37)	46.29 (44.79, 47.85)	35.04 (34.89, 35.20)	11.25 (9.75, 12.80)
Psychotic disorders	1.27 (1.12, 1.44)	3.03 (2.68, 3.44)	2.39 (2.35, 2.43)	0.64 (0.29, 1.05)
**Musculoskeletal disorders**	1.37 (1.34, 1.40)	136.32 (133.45, 139.25)	101.35 (101.07, 101.64)	34.97 (32.10, 37.90)
Joint pain	1.25 (1.22, 1.28)	113.43 (110.79, 116.13)	91.73 (91.46, 92.00)	21.71 (19.07, 24.41)
Myalgia	1.93 (1.87, 2.00)	35.52 (34.30, 36.78)	18.53 (18.43, 18.64)	16.98 (15.76, 18.24)
Myopathy	3.18 (2.64, 3.83)	1.11 (0.92, 1.33)	0.35 (0.33, 0.36)	0.76 (0.57, 0.98)
**Sensory disorders**	1.11 (1.08, 1.14)	88.07 (85.90, 90.30)	79.46 (79.20, 79.71)	8.62 (6.44, 10.85)
Hearing abnormalities and tinnitus	1.07 (1.04, 1.10)	68.84 (67.02, 70.71)	64.56 (64.34, 64.78)	4.28 (2.46, 6.16)
Vision abnormalities	1.21 (1.16, 1.26)	24.65 (23.60, 25.73)	20.47 (20.35, 20.58)	4.18 (3.14, 5.27)
Loss of smell	5.66 (4.81, 6.65)	1.42 (1.21, 1.67)	0.25 (0.24, 0.26)	1.17 (0.96, 1.42)
Loss of taste	3.09 (2.10, 4.55)	0.20 (0.14, 0.30)	0.07 (0.06, 0.07)	0.14 (0.07, 0.23)
**Other neurologic or related disorders**	1.45 (1.39, 1.51)	23.68 (22.71, 24.68)	16.38 (16.28, 16.48)	7.30 (6.33, 8.30)
Dizziness	1.43 (1.37, 1.49)	22.06 (21.14, 23.02)	15.47 (15.38, 15.57)	6.58 (5.66, 7.55)
Somnolence	1.60 (1.26, 2.04)	1.40 (1.10, 1.78)	0.87 (0.85, 0.90)	0.52 (0.23, 0.90)
Guillain-Barre syndrome	2.39 (1.54, 3.69)	0.20 (0.13, 0.31)	0.08 (0.08, 0.09)	0.12 (0.05, 0.22)
Encephalitis or encephalopathy	1.89 (1.21, 2.94)	0.16 (0.10, 0.25)	0.09 (0.08, 0.09)	0.08 (0.02, 0.17)
Transverse myelitis	2.63 (1.13, 6.17)	0.05 (0.02, 0.13)	0.02 (0.02, 0.02)	0.03 (0.00, 0.11)
**Any neurologic outcome**	1.31 (1.27, 1.35)	265.88 (258.73, 273.20)	210.18 (209.61, 210.76)	55.7 (48.54, 63.02)

*. Outcomes were ascertained from day 30 after the initial positive COVID-19 test result until end of follow up
†. Adjustment through inverse probability weighting using predefined and algorithmically selected high-dimensional variables.
CI, confidence interval; TIA, transient ischemic attack.

Supplementary Table 11. Subgroup analyses of the risks of incident post-acute COVID-19 composite neurologic outcomes compared to historical control

Risk Factors	COVID-19 vs Historical Control – Hazard Ratio (95%CI)*									
	Cerebro-vascular disorders	Cognition and memory disorders	Disorders of peripheral nerves	Episodic disorders	Extra-pyramidal and movement disorders	Mental health disorders	Musculo-skeletal disorders	Sensory disorders	Other neurologic or related disorders	Any neurologic outcome
**Age**										
≤65	1.49 (1.39, 1.60)	1.71 (1.61, 1.81)	1.30 (1.24, 1.37)	1.59 (1.46, 1.74)	1.50 (1.39, 1.61)	1.43 (1.36, 1.50)	1.50 (1.45, 1.55)	1.09 (1.05, 1.13)	1.44 (1.35, 1.53)	1.31 (1.25, 1.37)
>65	1.44 (1.28, 1.61)	1.50 (1.36, 1.65)	1.26 (1.19, 1.33)	1.27 (1.20, 1.35)	1.33 (1.21, 1.47)	1.22 (1.16, 1.28)	1.20 (1.16, 1.24)	1.13 (1.08, 1.17)	1.38 (1.30, 1.47)	1.27 (1.21, 1.33)
**Race**										
White	1.53 (1.42, 1.64)	1.7 (1.61, 1.80)	1.32 (1.26, 1.38)	1.39 (1.31, 1.48)	1.44 (1.35, 1.54)	1.31 (1.25, 1.36)	1.38 (1.34, 1.42)	1.12 (1.09, 1.16)	1.46 (1.40, 1.54)	1.30 (1.25, 1.35)
Black	1.57 (1.38, 1.79)	1.64 (1.49, 1.81)	1.17 (1.09, 1.26)	1.34 (1.22, 1.47)	1.39 (1.23, 1.58)	1.25 (1.17, 1.33)	1.18 (1.13, 1.24)	1.09 (1.02, 1.17)	1.32 (1.21, 1.44)	1.28 (1.19, 1.38)
**Sex**										
Male	1.48 (1.39, 1.57)	1.71 (1.63, 1.80)	1.32 (1.27, 1.37)	1.43 (1.35, 1.51)	1.43 (1.34, 1.52)	1.34 (1.29, 1.38)	1.39 (1.35, 1.42)	1.10 (1.07, 1.14)	1.46 (1.40, 1.53)	1.32 (1.28, 1.37)
Female	1.36 (1.06, 1.75)	1.77 (1.48, 2.12)	1.19 (1.08, 1.30)	1.21 (1.09, 1.35)	1.50 (1.26, 1.79)	1.11 (1.01, 1.22)	1.08 (1.00, 1.17)	1.17 (1.08, 1.28)	1.24 (1.10, 1.39)	1.17 (1.04, 1.30)
**Obesity**										
No	1.59 (1.46, 1.73)	1.73 (1.62, 1.85)	1.35 (1.28, 1.42)	1.38 (1.29, 1.48)	1.42 (1.31, 1.55)	1.37 (1.30, 1.43)	1.44 (1.39, 1.49)	1.14 (1.10, 1.19)	1.49 (1.40, 1.58)	1.37 (1.30, 1.43)
Yes	1.50 (1.39, 1.62)	1.65 (1.54, 1.76)	1.24 (1.18, 1.30)	1.34 (1.27, 1.43)	1.46 (1.34, 1.58)	1.25 (1.20, 1.31)	1.23 (1.20, 1.27)	1.07 (1.03, 1.11)	1.37 (1.28, 1.45)	1.25 (1.20, 1.30)
**Smoking**										
No/former	1.50 (1.42, 1.58)	1.65 (1.58, 1.71)	1.31 (1.27, 1.35)	1.43 (1.39, 1.48)	1.41 (1.34, 1.48)	1.34 (1.32, 1.37)	1.36 (1.33, 1.38)	0.99 (0.97, 1.01)	1.48 (1.41, 1.55)	1.29 (1.27, 1.30)
Yes	1.51 (1.32, 1.73)	1.81 (1.62, 2.04)	1.26 (1.16, 1.37)	1.34 (1.20, 1.50)	1.39 (1.22, 1.58)	1.39 (1.29, 1.50)	1.28 (1.21, 1.36)	1.18 (1.10, 1.26)	1.34 (1.21, 1.49)	1.40 (1.29, 1.52)

Risk Factors	COVID-19 vs Historical Control – Hazard Ratio (95%CI)*									
	Cerebro-vascular disorders	Cognition and memory disorders	Disorders of peripheral nerves	Episodic disorders	Extra-pyramidal and movement disorders	Mental health disorders	Musculo-skeletal disorders	Sensory disorders	Other neurologic or related disorders	Any neurologic outcome
**ADI**										
≤Median	1.59 (1.46, 1.74)	1.71 (1.60, 1.83)	1.28 (1.22, 1.35)	1.39 (1.30, 1.49)	1.48 (1.36, 1.61)	1.33 (1.27, 1.39)	1.35 (1.30, 1.40)	1.05 (1.01, 1.10)	1.44 (1.35, 1.53)	1.28 (1.22, 1.34)
>Median	1.52 (1.40, 1.65)	1.64 (1.53, 1.76)	1.28 (1.21, 1.35)	1.35 (1.25, 1.46)	1.42 (1.30, 1.55)	1.29 (1.23, 1.35)	1.33 (1.28, 1.37)	1.14 (1.10, 1.18)	1.43 (1.33, 1.53)	1.32 (1.26, 1.38)
**Diabetes**										
No	1.62 (1.49, 1.76)	1.74 (1.63, 1.85)	1.31 (1.25, 1.38)	1.34 (1.26, 1.42)	1.47 (1.36, 1.58)	1.30 (1.25, 1.35)	1.35 (1.31, 1.39)	1.12 (1.08, 1.16)	1.45 (1.38, 1.53)	1.30 (1.25, 1.36)
Yes	1.36 (1.26, 1.46)	1.49 (1.39, 1.59)	1.24 (1.17, 1.31)	1.50 (1.38, 1.64)	1.34 (1.23, 1.46)	1.33 (1.26, 1.40)	1.32 (1.27, 1.37)	1.08 (1.03, 1.13)	1.35 (1.26, 1.45)	1.25 (1.18, 1.32)
**Chronic kidney disease**										
No	1.55 (1.44, 1.66)	1.68 (1.58, 1.78)	1.30 (1.24, 1.35)	1.36 (1.28, 1.44)	1.44 (1.34, 1.54)	1.29 (1.24, 1.34)	1.33 (1.29, 1.36)	1.11 (1.07, 1.14)	1.45 (1.38, 1.52)	1.29 (1.24, 1.34)
Yes	1.41 (1.28, 1.55)	1.59 (1.46, 1.72)	1.21 (1.12, 1.30)	1.56 (1.40, 1.75)	1.41 (1.25, 1.59)	1.42 (1.32, 1.52)	1.43 (1.36, 1.50)	1.11 (1.05, 1.17)	1.35 (1.22, 1.50)	1.34 (1.25, 1.44)
**Hyperlipidemia**										
No	1.88 (1.67, 2.12)	2.03 (1.85, 2.23)	1.35 (1.27, 1.44)	1.31 (1.22, 1.41)	1.62 (1.46, 1.80)	1.37 (1.30, 1.45)	1.43 (1.37, 1.49)	1.06 (1.01, 1.11)	1.51 (1.39, 1.64)	1.34 (1.27, 1.41)
Yes	1.36 (1.28, 1.45)	1.46 (1.39, 1.55)	1.22 (1.17, 1.28)	1.39 (1.30, 1.49)	1.32 (1.23, 1.40)	1.25 (1.20, 1.31)	1.26 (1.22, 1.30)	1.11 (1.08, 1.15)	1.33 (1.27, 1.39)	1.25 (1.20, 1.31)
**Hypertension**										
No	1.56 (1.45, 1.69)	1.68 (1.58, 1.78)	1.28 (1.23, 1.34)	1.34 (1.27, 1.42)	1.44 (1.35, 1.54)	1.27 (1.22, 1.32)	1.34 (1.30, 1.37)	1.10 (1.06, 1.13)	1.41 (1.34, 1.48)	1.29 (1.24, 1.34)
Yes	1.46 (1.33, 1.61)	1.71 (1.55, 1.87)	1.29 (1.21, 1.38)	1.44 (1.30, 1.60)	1.45 (1.26, 1.67)	1.39 (1.30, 1.48)	1.34 (1.28, 1.40)	1.12 (1.06, 1.18)	1.48 (1.35, 1.62)	1.30 (1.22, 1.37)
**Immune dysfunction**										
No	1.55 (1.45, 1.65)	1.73 (1.64, 1.83)	1.29 (1.24, 1.34)	1.37 (1.29, 1.45)	1.44 (1.35, 1.53)	1.29 (1.24, 1.33)	1.35 (1.31, 1.38)	1.11 (1.08, 1.14)	1.44 (1.38, 1.51)	1.31 (1.26, 1.35)
Yes	1.33 (1.18, 1.51)	1.44 (1.29, 1.60)	1.29 (1.18, 1.41)	1.48 (1.30, 1.69)	1.35 (1.17, 1.55)	1.35 (1.24, 1.48)	1.32 (1.24, 1.40)	1.06 (0.99, 1.14)	1.24 (1.12, 1.37)	1.20 (1.09, 1.32)

*. Adjustment through inverse probability weighting using predefined and algorithmically selected high-dimensional variables.
†. Obesity was defined based on baseline BMI>30kg/m2
‡. Chronic kidney disease was defined based on baseline outpatient estimated Glomerular Filtration Rate< 60 mL/min/1.73 m2
CI, confidence interval; ADI, area deprivation index.

Supplementary Table 12. Risks and 12-month burdens of post-acute COVID-19 neurologic outcomes by care setting of the acute infection compared to historical control

Outcome*	Care setting†	Hazard ratio (95% CI)‡ COVID-19 vs Historical control	COVID-19 burden per 1000 persons at 12 months (95% CI)‡	Historical control burden per 1000 persons at 12 months (95% CI)‡	Burden difference per 1000 persons at 12 months (95% CI)‡
**Cerebrovascular**	Non-hospitalized	1.30 (1.23, 1.38)	12.37 (11.68, 13.10)	9.51 (9.43, 9.59)	2.86 (2.17, 3.59)
	Hospitalized	2.86 (2.52, 3.24)	26.92 (23.79, 30.45)		17.41 (14.28, 20.94)
	Admitted to intensive care	4.07 (3.29, 5.03)	38.13 (30.93, 46.96)		28.62 (21.42, 37.45)
Ischemic stroke	Non-hospitalized	1.21 (1.13, 1.30)	8.54 (7.99, 9.14)	7.05 (6.98, 7.12)	1.49 (0.93, 2.09)
	Hospitalized	3.03 (2.63, 3.48)	21.18 (18.43, 24.34)		14.13 (11.38, 17.29)
	Admitted to intensive care	4.09 (3.21, 5.22)	28.56 (22.48, 36.24)		21.5 (15.43, 29.19)
TIA	Non-hospitalized	1.41 (1.29, 1.54)	5.01 (4.59, 5.48)	3.56 (3.52, 3.61)	1.45 (1.02, 1.92)
	Hospitalized	2.36 (1.92, 2.89)	8.38 (6.84, 10.28)		4.82 (3.28, 6.71)
	Admitted to intensive care	2.99 (2.10, 4.27)	10.62 (7.45, 15.11)		7.05 (3.89, 11.55)
Hemorrhagic stroke	Non-hospitalized	1.32 (0.90, 1.93)	0.27 (0.19, 0.40)	0.21 (0.19, 0.22)	0.07 (-0.02, 0.19)
	Hospitalized	3.55 (2.03, 6.22)	0.73 (0.42, 1.28)		0.53 (0.21, 1.07)
	Admitted to intensive care	15.46 (6.30, 37.94)	3.18 (1.30, 7.79)		2.98 (1.09, 7.58)
Cerebral venous thrombosis	Non-hospitalized	0.74 (0.40, 1.38)	0.05 (0.03, 0.09)	0.07 (0.06, 0.07)	-0.02 (-0.04, 0.03)
	Hospitalized	7.13 (3.04, 16.74)	0.47 (0.20, 1.10)		0.40 (0.13, 1.04)
	Admitted to intensive care	21.16 (7.88, 56.81)	1.39 (0.52, 3.74)		1.33 (0.45, 3.68)
**Cognition and memory**	Non-hospitalized	1.36 (1.29, 1.42)	18.36 (17.51, 19.24)	13.55 (13.46, 13.65)	4.80 (3.96, 5.69)
	Hospitalized	3.62 (3.28, 4.00)	48.21 (43.75, 53.11)		34.65 (30.19, 39.55)
	Admitted to intensive care	4.83 (4.03, 5.79)	63.82 (53.53, 76.00)		50.26 (39.97, 62.44)
Memory problems	Non-hospitalized	1.36 (1.30, 1.43)	18.18 (17.34, 19.05)	13.37 (13.28, 13.47)	4.80 (3.97, 5.68)
	Hospitalized	3.61 (3.26, 3.98)	47.39 (42.99, 52.23)		34.01 (29.61, 38.85)
	Admitted to intensive care	4.70 (3.92, 5.63)	61.35 (51.47, 73.04)		47.97 (38.10, 59.66)
Alzheimer's disease	Non-hospitalized	1.25 (1.09, 1.44)	2.33 (2.03, 2.68)	1.86 (1.82, 1.89)	0.47 (0.17, 0.82)
	Hospitalized	3.33 (2.61, 4.23)	6.17 (4.85, 7.84)		4.31 (2.99, 5.99)
	Admitted to intensive care	4.42 (2.79, 7.00)	8.18 (5.17, 12.94)		6.32 (3.31, 11.08)

Outcome*	Care setting[†]	Hazard ratio (95% CI)[‡] COVID-19 vs Historical control	COVID-19 burden per 1000 persons at 12 months (95% CI)[‡]	Historical control burden per 1000 persons at 12 months (95% CI)[‡]	Burden difference per 1000 persons at 12 months (95% CI)[‡]
**Disorders of peripheral nerves**	Non-hospitalized	1.27 (1.23, 1.31)	33.28 (32.23, 34.36)	26.39 (26.26, 26.52)	6.89 (5.84, 7.98)
	Hospitalized	2.05 (1.87, 2.26)	53.41 (48.66, 58.61)		27.02 (22.27, 32.22)
	Admitted to intensive care	2.20 (1.84, 2.62)	57.07 (48.11, 67.64)		30.69 (21.73, 41.25)
Peripheral neuropathy	Non-hospitalized	1.23 (1.18, 1.28)	20.82 (20.01, 21.66)	16.96 (16.86, 17.07)	3.86 (3.05, 4.70)
	Hospitalized	1.98 (1.76, 2.22)	33.26 (29.69, 37.25)		16.29 (12.72, 20.28)
	Admitted to intensive care	2.13 (1.73, 2.62)	35.72 (29.13, 43.76)		18.75 (12.16, 26.80)
Paresthesia	Non-hospitalized	1.43 (1.36, 1.50)	12.35 (11.75, 12.99)	8.66 (8.59, 8.73)	3.69 (3.09, 4.33)
	Hospitalized	2.01 (1.70, 2.36)	17.30 (14.70, 20.35)		8.64 (6.04, 11.69)
	Admitted to intensive care	2.12 (1.61, 2.78)	18.24 (13.92, 23.89)		9.58 (5.26, 15.23)
Dysautonomia	Non-hospitalized	1.08 (1.00, 1.16)	6.55 (6.11, 7.04)	6.09 (6.02, 6.15)	0.47 (0.02, 0.95)
	Hospitalized	1.94 (1.61, 2.32)	11.74 (9.79, 14.09)		5.66 (3.70, 8.00)
	Admitted to intensive care	2.03 (1.40, 2.94)	12.30 (8.48, 17.82)		6.21 (2.40, 11.73)
Bell's palsy	Non-hospitalized	1.44 (1.21, 1.72)	1.00 (0.84, 1.19)	0.69 (0.67, 0.71)	0.31 (0.14, 0.50)
	Hospitalized	2.73 (1.76, 4.23)	1.89 (1.22, 2.93)		1.20 (0.53, 2.24)
	Admitted to intensive care	2.96 (1.66, 5.28)	2.05 (1.15, 3.66)		1.36 (0.46, 2.97)
**Episodic disorders**	Non-hospitalized	1.39 (1.34, 1.45)	19.33 (18.55, 20.14)	13.92 (13.82, 14.01)	5.41 (4.63, 6.23)
	Hospitalized	2.11 (1.85, 2.42)	29.16 (25.54, 33.29)		15.24 (11.62, 19.37)
	Admitted to intensive care	2.72 (2.10, 3.51)	37.37 (29.05, 48.01)		23.45 (15.13, 34.10)
Migraine	Non-hospitalized	1.28 (1.16, 1.41)	4.03 (3.65, 4.44)	3.15 (3.11, 3.20)	0.88 (0.50, 1.29)
	Hospitalized	2.97 (2.42, 3.65)	9.33 (7.60, 11.45)		6.18 (4.45, 8.30)
	Admitted to intensive care	4.83 (3.53, 6.61)	15.13 (11.09, 20.63)		11.97 (7.93, 17.47)
Epilepsy and seizure	Non-hospitalized	1.35 (1.28, 1.42)	12.36 (11.77, 12.98)	9.19 (9.11, 9.26)	3.17 (2.58, 3.80)
	Hospitalized	1.78 (1.47, 2.15)	16.28 (13.48, 19.66)		7.10 (4.30, 10.47)
	Admitted to intensive care	1.65 (1.12, 2.45)	15.15 (10.27, 22.32)		5.96 (1.08, 13.13)
Headache disorders	Non-hospitalized	1.67 (1.56, 1.79)	5.90 (5.51, 6.31)	3.53 (3.48, 3.57)	2.37 (1.99, 2.78)
	Hospitalized	2.08 (1.67, 2.59)	7.31 (5.87, 9.10)		3.79 (2.35, 5.57)
	Admitted to intensive care	3.46 (2.13, 5.63)	12.15 (7.49, 19.70)		8.63 (3.96, 16.17)
**Extrapyramidal and movement disorders**	Non-hospitalized	1.33 (1.26, 1.40)	12.46 (11.81, 13.15)	9.40 (9.32, 9.48)	3.06 (2.41, 3.75)
	Hospitalized	2.37 (2.07, 2.72)	22.15 (19.37, 25.32)		12.75 (9.97, 15.92)
	Admitted to intensive care	2.09 (1.61, 2.71)	19.52 (15.07, 25.26)		10.12 (5.68, 15.86)

Outcome*	Care setting†	Hazard ratio (95% CI)‡ COVID-19 vs Historical control	COVID-19 burden per 1000 persons at 12 months (95% CI)‡	Historical control burden per 1000 persons at 12 months (95% CI)‡	Burden difference per 1000 persons at 12 months (95% CI)‡
Abnormal involuntary movements	Non-hospitalized	1.41 (1.33, 1.50)	9.23 (8.68, 9.81)	6.53 (6.47, 6.60)	2.69 (2.14, 3.28)
	Hospitalized	2.36 (2.03, 2.75)	15.37 (13.21, 17.88)		8.84 (6.68, 11.35)
	Admitted to intensive care	1.97 (1.45, 2.69)	12.83 (9.43, 17.46)		6.30 (2.90, 10.92)
Tremor	Non-hospitalized	1.30 (1.18, 1.43)	3.76 (3.41, 4.14)	2.89 (2.85, 2.93)	0.87 (0.52, 1.25)
	Hospitalized	2.07 (1.61, 2.66)	5.98 (4.65, 7.68)		3.09 (1.76, 4.79)
	Admitted to intensive care	1.74 (1.15, 2.64)	5.04 (3.33, 7.61)		2.15 (0.44, 4.72)
Parkinson-like disease	Non-hospitalized	1.10 (0.95, 1.27)	1.99 (1.73, 2.30)	1.82 (1.78, 1.85)	0.18 (-0.09, 0.48)
	Hospitalized	2.29 (1.70, 3.10)	4.16 (3.08, 5.61)		2.34 (1.26, 3.80)
	Admitted to intensive care	1.85 (0.98, 3.50)	3.36 (1.78, 6.35)		1.54 (-0.04, 4.53)
Dystonia	Non-hospitalized	1.37 (1.14, 1.65)	0.96 (0.80, 1.15)	0.70 (0.68, 0.72)	0.26 (0.10, 0.45)
	Hospitalized	2.24 (1.42, 3.53)	1.56 (0.99, 2.46)		0.86 (0.29, 1.76)
	Admitted to intensive care	2.13 (1.08, 4.22)	1.48 (0.75, 2.93)		0.79 (0.05, 2.23)
Myoclonus	Non-hospitalized	1.12 (0.81, 1.55)	0.35 (0.25, 0.49)	0.31 (0.30, 0.33)	0.04 (-0.06, 0.17)
	Hospitalized	2.75 (1.72, 4.41)	0.86 (0.54, 1.38)		0.55 (0.23, 1.07)
	Admitted to intensive care	4.15 (2.08, 8.26)	1.30 (0.65, 2.59)		0.99 (0.34, 2.28)
**Mental health disorders**	Non-hospitalized	1.26 (1.22, 1.30)	81.40 (79.18, 83.68)	65.22 (64.98, 65.46)	16.18 (13.96, 18.46)
	Hospitalized	2.24 (2.05, 2.44)	140.16 (129.30, 151.85)		74.94 (64.08, 86.63)
	Admitted to intensive care	2.75 (2.30, 3.28)	169.34 (143.92, 198.71)		104.12 (78.69, 133.49)
Major depressive disorders	Non-hospitalized	1.18 (1.14, 1.21)	53.34 (51.81, 54.91)	45.49 (45.30, 45.67)	7.85 (6.32, 9.42)
	Hospitalized	2.16 (1.98, 2.36)	95.69 (87.92, 104.12)		50.21 (42.43, 58.64)
	Admitted to intensive care	2.34 (1.93, 2.83)	103.20 (86.10, 123.45)		57.71 (40.61, 77.97)
Stress and adjustment disorders	Non-hospitalized	1.28 (1.25, 1.32)	50.51 (49.01, 52.05)	39.56 (39.38, 39.73)	10.95 (9.45, 12.49)
	Hospitalized	2.08 (1.89, 2.29)	80.46 (73.41, 88.14)		40.90 (33.86, 48.59)
	Admitted to intensive care	2.09 (1.70, 2.57)	80.91 (66.26, 98.62)		41.36 (26.71, 59.07)
Anxiety disorders	Non-hospitalized	1.27 (1.23, 1.31)	43.93 (42.65, 45.26)	34.69 (34.53, 34.84)	9.25 (7.96, 10.57)
	Hospitalized	2.25 (2.05, 2.47)	76.42 (69.96, 83.46)		41.74 (35.27, 48.77)
	Admitted to intensive care	2.44 (2.03, 2.93)	82.48 (69.06, 98.35)		47.79 (34.38, 63.67)
Psychotic disorders	Non-hospitalized	1.01 (0.89, 1.15)	2.35 (2.08, 2.67)	2.32 (2.29, 2.36)	0.03 (-0.25, 0.34)
	Hospitalized	3.78 (2.92, 4.88)	8.75 (6.77, 11.30)		6.42 (4.44, 8.98)
	Admitted to intensive care	3.30 (1.84, 5.91)	7.64 (4.27, 13.66)		5.32 (1.95, 11.33)

Outcome*	Care setting[†]	Hazard ratio (95% CI)[‡] COVID-19 vs Historical control	COVID-19 burden per 1000 persons at 12 months (95% CI)[‡]	Historical control burden per 1000 persons at 12 months (95% CI)[‡]	Burden difference per 1000 persons at 12 months (95% CI)[‡]
**Musculoskeletal**	Non-hospitalized	1.35 (1.32, 1.38)	134.06 (131.45, 136.72)	100.98 (100.70, 101.27)	33.08 (30.46, 35.74)
	Hospitalized	2.19 (2.05, 2.34)	208.02 (196.09, 220.57)		107.04 (95.11, 119.59)
	Admitted to intensive care	2.61 (2.28, 2.99)	242.77 (215.43, 272.94)		141.79 (114.44, 171.96)
Joint pain	Non-hospitalized	1.29 (1.26, 1.32)	116.65 (114.27, 119.08)	91.47 (91.20, 91.74)	25.18 (22.79, 27.61)
	Hospitalized	1.61 (1.49, 1.74)	143.01 (133.28, 153.39)		51.54 (41.80, 61.92)
	Admitted to intensive care	1.58 (1.34, 1.86)	140.5 (120.53, 163.45)		49.02 (29.06, 71.98)
Myalgia	Non-hospitalized	1.57 (1.52, 1.63)	28.38 (27.42, 29.38)	18.15 (18.04, 18.26)	10.23 (9.27, 11.23)
	Hospitalized	4.63 (4.30, 4.98)	81.32 (75.79, 87.23)		63.17 (57.64, 69.08)
	Admitted to intensive care	6.66 (5.79, 7.66)	114.88 (100.7, 130.92)		96.74 (82.56, 112.78)
Myopathy	Non-hospitalized	1.82 (1.46, 2.27)	0.62 (0.50, 0.77)	0.34 (0.33, 0.35)	0.28 (0.16, 0.43)
	Hospitalized	7.69 (5.49, 10.78)	2.61 (1.86, 3.65)		2.27 (1.52, 3.31)
	Admitted to intensive care	42.46 (30.16, 59.78)	14.32 (10.19, 20.10)		13.98 (9.86, 19.76)
**Sensory disorders**	Non-hospitalized	1.10 (1.07, 1.12)	86.64 (84.68, 88.65)	79.31 (79.06, 79.56)	7.33 (5.37, 9.35)
	Hospitalized	1.27 (1.18, 1.37)	99.76 (92.72, 107.31)		20.45 (13.40, 28.00)
	Admitted to intensive care	1.33 (1.13, 1.58)	104.43 (89.19, 122.1)		25.12 (9.88, 42.79)
Hearing abnormalities and tinnitus	Non-hospitalized	1.06 (1.03, 1.09)	68.32 (66.61, 70.07)	64.45 (64.22, 64.67)	3.88 (2.16, 5.63)
	Hospitalized	1.11 (1.02, 1.21)	71.43 (65.72, 77.61)		6.98 (1.27, 13.16)
	Admitted to intensive care	1.21 (1.00, 1.46)	77.36 (64.60, 92.52)		12.92 (0.15, 28.08)
Vision abnormalities	Non-hospitalized	1.19 (1.14, 1.23)	24.17 (23.25, 25.12)	20.41 (20.29, 20.52)	3.76 (2.84, 4.72)
	Hospitalized	1.60 (1.43, 1.79)	32.38 (28.99, 36.15)		11.97 (8.59, 15.74)
	Admitted to intensive care	1.64 (1.33, 2.03)	33.32 (27.00, 41.07)		12.91 (6.60, 20.67)
Loss of smell	Non-hospitalized	5.56 (4.79, 6.45)	1.40 (1.20, 1.62)	0.25 (0.24, 0.26)	1.14 (0.95, 1.37)
	Hospitalized	6.21 (3.59, 10.73)	1.56 (0.90, 2.69)		1.31 (0.65, 2.44)
	Admitted to intensive care	10.54 (5.54, 20.08)	2.65 (1.39, 5.04)		2.38 (1.13, 4.77)
Loss of taste	Non-hospitalized	2.83 (1.86, 4.31)	0.18 (0.12, 0.28)	0.07 (0.06, 0.07)	0.12 (0.06, 0.22)
	Hospitalized	6.54 (2.52, 16.98)	0.43 (0.16, 1.11)		0.36 (0.10, 1.04)
	Admitted to intensive care	13.75 (3.46, 54.60)	0.90 (0.23, 3.56)		0.83 (0.16, 3.49)
**Other neurologic or related disorders**	Non-hospitalized	1.4 (1.34, 1.45)	22.64 (21.79, 23.53)	16.28 (16.17, 16.38)	6.37 (5.51, 7.25)
	Hospitalized	2.38 (2.15, 2.64)	38.33 (34.61, 42.44)		22.05 (18.33, 26.16)
	Admitted to intensive care	2.58 (2.09, 3.19)	41.51 (33.70, 51.08)		25.23 (17.42, 34.80)

Outcome*	Care setting[†]	Hazard ratio (95% CI)[‡] COVID-19 vs Historical control	COVID-19 burden per 1000 persons at 12 months (95% CI)[‡]	Historical control burden per 1000 persons at 12 months (95% CI)[‡]	Burden difference per 1000 persons at 12 months (95% CI)[‡]
Dizziness	Non-hospitalized	1.39 (1.33, 1.44)	21.27 (20.45, 22.13)	15.39 (15.29, 15.49)	5.88 (5.06, 6.74)
	Hospitalized	2.38 (2.14, 2.65)	36.23 (32.61, 40.25)		20.84 (17.22, 24.86)
	Admitted to intensive care	2.53 (2.02, 3.17)	38.52 (30.90, 47.98)		23.13 (15.51, 32.59)
Somnolence	Non-hospitalized	1.33 (1.13, 1.56)	1.13 (0.96, 1.33)	0.85 (0.83, 0.88)	0.28 (0.11, 0.48)
	Hospitalized	1.58 (1.03, 2.43)	1.35 (0.88, 2.08)		0.50 (0.02, 1.22)
	Admitted to intensive care	2.23 (1.30, 3.84)	1.91 (1.11, 3.28)		1.05 (0.25, 2.42)
Guillain-Barre syndrome	Non-hospitalized	2.06 (1.26, 3.37)	0.17 (0.10, 0.28)	0.08 (0.07, 0.09)	0.09 (0.02, 0.19)
	Hospitalized	7.10 (2.54, 19.88)	0.58 (0.21, 1.63)		0.50 (0.13, 1.55)
	Admitted to intensive care	9.43 (4.12, 21.59)	0.77 (0.34, 1.77)		0.69 (0.26, 1.69)
Encephalitis or encephalopathy	Non-hospitalized	2.01 (1.22, 3.29)	0.16 (0.10, 0.26)	0.08 (0.07, 0.09)	0.08 (0.02, 0.18)
	Hospitalized	4.78 (2.38, 9.59)	0.38 (0.19, 0.75)		0.30 (0.11, 0.68)
	Admitted to intensive care	6.65 (2.63, 16.81)	0.52 (0.21, 1.32)		0.44 (0.13, 1.24)
Transverse myelitis	Non-hospitalized	1.55 (0.59, 4.11)	0.04 (0.02, 0.11)	0.03 (0.02, 0.03)	0.02 (-0.01, 0.08)
	Hospitalized	3.90 (1.37, 11.06)	0.11 (0.04, 0.30)		0.08 (0.01, 0.27)
	Admitted to intensive care	5.17 (1.21, 22.02)	0.14 (0.03, 0.60)		0.11 (0.01, 0.57)
**Any neurologic outcome**	Non-hospitalized	1.24 (1.20, 1.28)	252.70 (245.54, 260.03)	209.79 (209.22, 210.37)	42.91 (35.74, 50.24)
	Hospitalized	2.64 (2.36, 2.96)	463.47 (426.75, 501.78)		253.68 (216.96, 291.98)
	Admitted to intensive care	3.68 (2.78, 4.88)	579.94 (480.34, 683.14)		370.14 (270.54, 473.35)

*. Outcomes were ascertained from day 30 after the initial positive COVID-19 test result until end of follow up
†. Based on care received within the first 30 days after a positive COVID-19 test result.
‡. Adjustment through inverse probability weighting using predefined variables and algorithmically selected high-dimensional variables.
CI, confidence interval; TIA, transient ischemic attack.

Supplementary Table 13a. Sensitivity analysis for any neurologic outcome compared to the contemporary control

Analysis	COVID-19 vs Contemporary Control - Hazard ratio (95% CI)*									
	Cerebro-vascular disorders	Cognition and memory disorders	Disorders of peripheral nerves	Episodic disorders	Extra-pyramidal and movement disorders	Mental health disorders	Musculo-skeletal disorders	Sensory disorders	Other neurologic or related disorders	Any neurologic outcome
Did not use any high dimensional variables	1.56 (1.48, 1.63)	1.68 (1.61, 1.74)	1.36 (1.32, 1.40)	1.39 (1.33, 1.44)	1.40 (1.33, 1.46)	1.46 (1.43, 1.50)	1.52 (1.49, 1.55)	1.23 (1.21, 1.26)	1.50 (1.45, 1.55)	1.48 (1.44, 1.52)
Doubly robust	1.19 (1.12, 1.25)	1.50 (1.42, 1.58)	1.17 (1.12, 1.22)	1.18 (1.12, 1.24)	1.23 (1.16, 1.32)	1.32 (1.28, 1.37)	1.28 (1.25, 1.32)	1.19 (1.16, 1.23)	1.21 (1.15, 1.28)	1.47 (1.43, 1.50)

*. Outcomes were ascertained from day 30 after the initial positive COVID-19 test result until end of follow up.
CI, confidence interval.

Supplementary Table 13b. Sensitivity analysis for any neurologic outcome compared to the historical control

Analysis	COVID-19 vs Historical Control - Hazard ratio (95% CI)*									
	Cerebro-vascular disorders	Cognition and memory disorders	Disorders of peripheral nerves	Episodic disorders	Extra-pyramidal and movement disorders	Mental health disorders	Musculo-skeletal disorders	Sensory disorders	Other neurologic or related disorders	Any neurologic outcome
Did not use any high dimensional variables	1.50 (1.43, 1.58)	1.67 (1.61, 1.74)	1.36 (1.32, 1.40)	1.49 (1.44, 1.55)	1.45 (1.38, 1.52)	1.39 (1.35, 1.42)	1.46 (1.43, 1.49)	1.11 (1.08, 1.13)	1.51 (1.46, 1.57)	1.38 (1.35, 1.42)
Doubly robust	1.25 (1.31, 1.18)	1.43 (1.35, 1.51)	1.14 (1.09, 1.19)	1.24 (1.18, 1.31)	1.24 (1.17, 1.32)	1.24 (1.20, 1.28)	1.21 (1.18, 1.24)	1.05 (1.02, 1.08)	1.20 (1.13, 1.26)	1.36 (1.33, 1.40)

*. Outcomes were ascertained from day 30 after the initial positive COVID-19 test result until end of follow up.
CI, confidence interval.

Supplementary Table 14a. Sensitivity analysis for any neurologic outcome by care setting of the acute infection compared to the contemporary control

Analysis	Care setting†	COVID-19 vs Contemporary Control – Hazard ratio (95% CI)									
		Cerebro-vascular disorders*	Cognition and memory disorders*	Disorders of peripheral nerves*	Episodic disorders*	Extra-pyramidal and movement disorders*	Mental health disorders*	Musculo-skeletal disorders*	Sensory disorders*	Other neurologic or related disorders*	Any neurologic outcome*
Did not use any high dimensional variables	Non-hospitalized	1.37 (1.29, 1.45)	1.38 (1.32, 1.45)	1.29 (1.24, 1.33)	1.31 (1.25, 1.36)	1.29 (1.23, 1.37)	1.34 (1.30, 1.38)	1.42 (1.39, 1.45)	1.23 (1.20, 1.26)	1.40 (1.34, 1.45)	1.40 (1.36, 1.43)
	Hospitalized	3.00 (2.64, 3.40)	3.69 (3.34, 4.07)	2.09 (1.90, 2.29)	1.98 (1.73, 2.27)	2.31 (2.02, 2.65)	2.38 (2.19, 2.60)	2.30 (2.15, 2.46)	1.43 (1.32, 1.54)	2.38 (2.15, 2.64)	2.37 (2.16, 2.61)
	Admitted to intensive care	4.27 (3.45, 5.28)	4.92 (4.11, 5.90)	2.23 (1.87, 2.66)	2.55 (1.98, 3.30)	2.03 (1.57, 2.64)	2.93 (2.45, 3.50)	2.74 (2.39, 3.15)	1.50 (1.27, 1.77)	2.59 (2.09, 3.20)	3.08 (2.51, 3.77)
Doubly robust	Non-hospitalized	1.28 (1.22, 1.34)	1.34 (1.29, 1.39)	1.18 (1.15, 1.21)	1.21 (1.17, 1.25)	1.22 (1.17, 1.28)	1.22 (1.2, 1.25)	1.26 (1.23, 1.28)	1.22 (1.19, 1.24)	1.28 (1.24, 1.32)	1.31 (1.28, 1.34)
	Hospitalized	1.89 (1.74, 2.06)	2.27 (2.12, 2.43)	1.47 (1.38, 1.57)	1.59 (1.46, 1.74)	1.56 (1.43, 1.71)	1.88 (1.77, 1.99)	1.76 (1.68, 1.84)	1.26 (1.2, 1.33)	1.67 (1.56, 1.79)	1.82 (1.71, 1.95)
	Admitted to intensive care	2.64 (2.32, 2.99)	2.89 (2.59, 3.22)	1.77 (1.59, 1.97)	2.39 (2.08, 2.74)	1.72 (1.47, 2.01)	2.36 (2.14, 2.59)	2.09 (1.93, 2.27)	1.3 (1.19, 1.43)	1.75 (1.55, 1.98)	2.22 (1.97, 2.50)

*. Outcomes were ascertained from day 30 after the initial positive COVID-19 test result until end of follow up.
†. Based on care received within the first 30 days after a positive COVID-19 test result.
CI, confidence interval.

Supplementary Table 14b. Sensitivity analysis for any neurologic outcome by care setting of the acute infection compared to the historical control

Analysis	Care setting†	COVID-19 vs Historical Control – Hazard ratio (95% CI)									
		Cerebro-vascular disorders*	Cognition and memory disorders*	Disorders of peripheral nerves*	Episodic disorders*	Extra-pyramidal and movement disorders*	Mental health disorders*	Musculo-skeletal disorders*	Sensory disorders*	Other neurologic or related disorders*	Any neurologic outcome*
Did not use any high dimensional variables	Non-hospitalized	1.32 (1.25, 1.40)	1.38 (1.32, 1.45)	1.28 (1.24, 1.32)	1.41 (1.35, 1.47)	1.35 (1.28, 1.42)	1.27 (1.24, 1.31)	1.37 (1.34, 1.40)	1.10 (1.07, 1.13)	1.41 (1.36, 1.47)	1.30 (1.27, 1.34)
	Hospitalized	2.90 (2.56, 3.28)	3.69 (3.34, 4.07)	2.08 (1.89, 2.28)	2.14 (1.87, 2.45)	2.41 (2.10, 2.76)	2.26 (2.08, 2.47)	2.21 (2.07, 2.37)	1.28 (1.18, 1.38)	2.41 (2.17, 2.68)	2.21 (2.01, 2.43)
	Admitted to intensive care	4.13 (3.34, 5.11)	4.93 (4.11, 5.90)	2.22 (1.87, 2.65)	2.75 (2.13, 3.56)	2.12 (1.63, 2.75)	2.78 (2.33, 3.32)	2.64 (2.30, 3.03)	1.34 (1.14, 1.58)	2.62 (2.12, 3.24)	2.87 (2.34, 3.51)
Doubly robust	Non-hospitalized	1.28 (1.21, 1.36)	1.35 (1.29, 1.42)	1.24 (1.20, 1.28)	1.35 (1.29, 1.40)	1.31 (1.24, 1.38)	1.22 (1.19, 1.26)	1.32 (1.29, 1.34)	1.11 (1.08, 1.13)	1.36 (1.30, 1.41)	1.15 (1.11, 1.19)
	Hospitalized	2.77 (2.41, 3.19)	3.60 (3.23, 4.02)	1.92 (1.73, 2.12)	2.00 (1.72, 2.33)	2.24 (1.94, 2.59)	2.23 (2.01, 2.48)	2.08 (1.93, 2.24)	1.27 (1.16, 1.39)	2.23 (2.00, 2.50)	2.05 (1.28, 3.29)
	Admitted to intensive care	4.07 (3.30, 5.02)	4.96 (4.14, 5.94)	2.15 (1.81, 2.56)	2.57 (1.98, 3.35)	2.08 (1.61, 2.69)	2.59 (2.13, 3.14)	2.46 (2.16, 2.81)	1.37 (1.16, 1.61)	2.53 (2.05, 3.12)	2.17 (1.75, 2.69)

*. Outcomes were ascertained from day 30 after the initial positive COVID-19 test result until end of follow up.
†. Based on care received within the first 30 days after a positive COVID-19 test result.
CI, confidence interval.

Supplementary Table 15. Positive and negative outcome controls

Outcome*	Hazard Ratio (95% CI)	
	**COVID-19 vs contemporary control (as the reference)**	**COVID-19 vs historical control (as the reference)**
**Positive outcome control**		
Fatigue	1.63 (1.58, 1.68)	2.03 (1.97, 2.10)
**Negative outcome control**		
Lichen simplex chronicus	0.97 (0.86, 1.11)	1.09 (0.96, 1.24)
Polycythemia vera	0.99 (0.77, 1.28)	1.08 (0.84, 1.39)
Solar urticaria	1.05 (0.88, 1.25)	0.97 (0.81, 1.15)

*. Outcomes were ascertained from day 30 after the initial positive COVID-19 test result until end of follow up

†. Adjustment through inverse probability weighting using predefined and algorithmically selected high-dimensional variables.

CI, confidence interval.

Supplementary Table 16. Negative exposure control: risks and 12-month burdens of neurologic outcomes of those vaccinated for influenza on even-numbered days compared to those vaccinated on odd-numbered days

Outcome*	Hazard Ratio (95% CI)†	Influenza vaccination on even days burden per 1000 persons at 12 months (95% CI) †	Influenza vaccination on odd days burden per 1000 persons at 12 months (95% CI) †	Absolute burden difference per 1000 persons at 12 months (95% CI) †
	Influenza vaccination on even days vs odd days			
**Cerebrovascular disorders**	1.00 (0.94, 1.05)	13.75 (13.03, 14.51)	13.80 (13.49, 14.10)	-0.04 (-0.76, 0.72)
Ischemic stroke	1.04 (0.98, 1.11)	10.44 (9.81, 11.10)	10.01 (9.75, 10.27)	0.43 (-0.20, 1.09)
TIA	0.95 (0.88, 1.04)	5.31 (4.87, 5.78)	5.56 (5.37, 5.75)	-0.25 (-0.69, 0.22)
Hemorrhagic stroke	1.21 (0.84, 1.73)	0.31 (0.21, 0.44)	0.25 (0.21, 0.29)	0.05 (-0.04, 0.18)
Cerebral venous thrombosis	0.88 (0.48, 1.62)	0.10 (0.05, 0.19)	0.11 (0.09, 0.14)	-0.01 (-0.06, 0.07)
**Cognition and memory disorders**	1.00 (0.96, 1.05)	19.41 (18.55, 20.32)	19.37 (19.01, 19.73)	0.04 (-0.82, 0.95)
Memory problems	1.01 (0.97, 1.06)	19.44 (18.58, 20.35)	19.21 (18.86, 19.57)	0.23 (-0.64, 1.13)
Alzheimer’s disease	0.96 (0.84, 1.09)	2.20 (1.92, 2.51)	2.29 (2.17, 2.41)	-0.10 (-0.37, 0.22)
**Disorders of peripheral nerves**	0.99 (0.96, 1.03)	39.30 (38.03, 40.61)	39.51 (38.99, 40.03)	-0.21 (-1.48, 1.10)
Peripheral neuropathy	1.01 (0.97, 1.05)	26.43 (25.42, 27.49)	26.18 (25.76, 26.60)	0.26 (-0.76, 1.31)
Paresthesia	0.96 (0.91, 1.01)	12.85 (12.16, 13.58)	13.38 (13.09, 13.68)	-0.54 (-1.23, 0.19)
Dysautonomia	0.98 (0.92, 1.05)	8.34 (7.78, 8.93)	8.50 (8.26, 8.73)	-0.16 (-0.71, 0.43)
Bell’s palsy	0.90 (0.73, 1.12)	0.85 (0.69, 1.05)	0.94 (0.86, 1.02)	-0.09 (-0.26, 0.11)
**Episodic disorders**	0.97 (0.92, 1.02)	16.98 (16.17, 17.83)	17.51 (17.16, 17.85)	-0.53 (-1.34, 0.33)
Migraine disorders	0.99 (0.93, 1.05)	10.42 (9.79, 11.08)	10.55 (10.28, 10.81)	-0.13 (-0.76, 0.54)
Epilepsy and seizures	0.97 (0.87, 1.07)	3.98 (3.60, 4.39)	4.11 (3.95, 4.28)	-0.14 (-0.51, 0.28)
Headache disorders	0.95 (0.88, 1.04)	5.42 (4.98, 5.90)	5.68 (5.48, 5.87)	-0.26 (-0.70, 0.22)
**Extrapyramidal and movement disorders**	0.99 (0.94, 1.04)	14.98 (14.22, 15.77)	15.19 (14.87, 15.51)	-0.21 (-0.97, 0.58)
Abnormal involuntary movements	1.01 (0.95, 1.07)	11.38 (10.73, 12.07)	11.28 (11.01, 11.55)	0.10 (-0.55, 0.79)
Tremor	1.02 (0.93, 1.12)	4.95 (4.53, 5.41)	4.85 (4.67, 5.03)	0.10 (-0.32, 0.56)
Parkinson-like disease	1.05 (0.93, 1.19)	2.61 (2.31, 2.95)	2.48 (2.36, 2.61)	0.12 (-0.18, 0.47)
Dystonia	0.89 (0.73, 1.08)	1.02 (0.84, 1.24)	1.15 (1.06, 1.23)	-0.13 (-0.31, 0.09)
Myoclonus	0.99 (0.76, 1.27)	0.59 (0.46, 0.76)	0.60 (0.54, 0.66)	-0.01 (-0.14, 0.16)
**Mental health disorders**	1.00 (0.97, 1.03)	73.92 (71.81, 76.10)	74.17 (73.30, 75.04)	-0.25 (-2.37, 1.93)
Major depressive disorders	1.01 (0.98, 1.05)	50.12 (48.56, 51.73)	49.47 (48.83, 50.11)	0.65 (-0.91, 2.26)
Stress and adjustment disorders	0.99 (0.96, 1.03)	43.77 (42.31, 45.27)	44.02 (43.42, 44.62)	-0.25 (-1.71, 1.25)

Outcome*	Hazard Ratio (95% CI)†	Influenza vaccination on even days burden per 1000 persons at 12 months (95% CI) †	Influenza vaccination on odd days burden per 1000 persons at 12 months (95% CI) †	Absolute burden difference per 1000 persons at 12 months (95% CI) †
	Influenza vaccination on even days vs odd days			
Anxiety disorders	1.01 (0.98, 1.05)	40.55 (39.22, 41.92)	40.14 (39.60, 40.69)	0.41 (-0.92, 1.77)
Psychotic disorders	0.99 (0.87, 1.12)	2.32 (2.04, 2.64)	2.35 (2.23, 2.48)	-0.03 (-0.31, 0.29)
**Musculoskeletal disorders**	0.99 (0.97, 1.01)	143.85 (141.03, 146.71)	145.33 (144.17, 146.49)	-1.48 (-4.30, 1.38)
Joint pain	1.00 (0.97, 1.02)	126.51 (123.91, 129.17)	126.93 (125.85, 128.00)	-0.41 (-3.02, 2.25)
Myalgia	1.00 (0.96, 1.03)	32.47 (31.35, 33.63)	32.63 (32.17, 33.10)	-0.16 (-1.28, 1.00)
Myopathy	1.06 (0.84, 1.34)	0.72 (0.57, 0.90)	0.67 (0.61, 0.74)	0.04 (-0.11, 0.23)
**Sensory disorders**	1.02 (1.00, 1.05)	100.11 (97.88, 102.40)	98.01 (97.10, 98.91)	2.11 (-0.13, 4.39)
Vision abnormalities	1.02 (0.99, 1.04)	77.49 (75.58, 79.44)	76.30 (75.52, 77.08)	1.19 (-0.72, 3.15)
Hearing abnormalities and tinnitus	1.01 (0.98, 1.05)	28.96 (27.89, 30.08)	28.57 (28.13, 29.01)	0.40 (-0.68, 1.51)
Loss of smell	1.17 (0.91, 1.49)	0.65 (0.50, 0.83)	0.55 (0.49, 0.61)	0.09 (-0.05, 0.27)
Loss of taste	1.05 (0.62, 1.80)	0.14 (0.08, 0.24)	0.13 (0.10, 0.16)	0.01 (-0.05, 0.11)
**Other neurologic or related disorders**	1.01 (0.97, 1.05)	26.76 (25.74, 27.82)	26.44 (26.02, 26.86)	0.32 (-0.70, 1.38)
Dizziness	1.02 (0.98, 1.06)	25.74 (24.74, 26.78)	25.28 (24.87, 25.69)	0.46 (-0.54, 1.50)
Somnolence	0.96 (0.80, 1.16)	1.10 (0.91, 1.33)	1.14 (1.06, 1.23)	-0.04 (-0.23, 0.18)
Guillain-Barre syndrome	0.97 (0.48, 1.99)	0.07 (0.04, 0.15)	0.07 (0.05, 0.10)	0.00 (-0.04, 0.07)
Encephalitis or encephalopathy	0.88 (0.50, 1.56)	0.12 (0.07, 0.22)	0.14 (0.11, 0.17)	-0.02 (-0.07, 0.08)
Transverse myelitis	0.32 (0.08, 1.35)	0.02 (0.00, 0.07)	0.05 (0.03, 0.07)	-0.03 (-0.05, 0.02)
**Any neurologic outcome**	0.99 (0.96, 1.01)	271.07 (265.26, 276.99)	274.32 (271.93, 276.71)	-3.25 (-9.07, 2.67)

*. Outcomes were ascertained from day 30 after the initial positive COVID-19 test result until end of follow up

†. Adjustment through inverse probability weighting using predefined and algorithmically selected high-dimensional variables.

CI, confidence interval; TIA, transient ischemic attack.
